# How People With a Bipolar Disorder Diagnosis Talk About Personal Recovery in Peer Online Support Forums: Corpus Framework Analysis Using the POETIC Framework

**DOI:** 10.2196/46544

**Published:** 2023-11-08

**Authors:** Glorianna Jagfeld, Fiona Lobban, Chloe Humphreys, Paul Rayson, Steven Huntley Jones

**Affiliations:** 1 Division of Health Research, Spectrum Centre for Mental Health Research, Lancaster University, Lancaster, United Kingdom; 2 UCREL Research Centre, School of Computing and Communications, Lancaster University, Lancaster, United Kingdom; 3 Faculty of Arts and Social Sciences, Department of Linguistics and English Language, Lancaster University, Lancaster, United Kingdom

**Keywords:** bipolar disorder, personal recovery, peer online support forums, natural language processing, corpus linguistics, social media, online support, recovery

## Abstract

**Background:**

Personal recovery is of particular value in bipolar disorder, where symptoms often persist despite treatment. We previously defined the POETIC (Purpose and Meaning, Optimism and Hope, Empowerment, Tensions, Identity, Connectedness) framework for personal recovery in bipolar disorder. So far, personal recovery has only been studied in researcher-constructed environments (eg, interviews and focus groups). Support forum posts can serve as a complementary naturalistic data resource to understand the lived experience of personal recovery.

**Objective:**

This study aimed to answer the question “What can online support forum posts reveal about the experience of personal recovery in bipolar disorder in relation to the POETIC framework?”

**Methods:**

By integrating natural language processing, corpus linguistics, and health research methods, this study analyzed public, bipolar disorder support forum posts relevant to the lived experience of personal recovery. By comparing 4462 personal recovery–relevant posts by 1982 users to 25,197 posts not relevant to personal recovery, we identified 130 significantly overused key lemmas. Key lemmas were coded according to the POETIC framework.

**Results:**

Personal recovery–related discussions primarily focused on 3 domains: “Purpose and meaning” (particularly reproductive decisions and work), “Connectedness” (romantic relationships and social support), and “Empowerment” (self-management and personal responsibility). This study confirmed the validity of the POETIC framework to capture personal recovery experiences shared on the web and highlighted new aspects beyond previous studies using interviews and focus groups.

**Conclusions:**

This study is the first to analyze naturalistic data on personal recovery in bipolar disorder. By indicating the key areas that people focus on in personal recovery when posting freely and the language they use, this study provides helpful starting points for formal and informal carers to understand the concerns of people diagnosed with a bipolar disorder and to consider how to best offer support.

## Introduction

Bipolar disorder (BD) is a severe mental health (MH) problem characterized by recurring episodes of depressed and elevated mood [1]. Its lifetime prevalence ranges from 0.1% to 2.6% internationally [2]. BD is associated with lower quality of life [3] and high suicide risk [4]. Therefore, fostering recovery and living well with BD are important societal tasks.

MH care agendas increasingly focus on enhancing personal recovery (PR), defined as “a way of living a satisfying, hopeful life even with the limitations caused by the illness” [5]. This contrasts with a previously narrower focus on reducing symptoms (clinical recovery). PR might be of particular value in BD [6], where symptoms often persist despite treatment, but has been underresearched to date [7]. Jagfeld et al [8] (hereafter the POETIC review) recently synthesized 12 qualitative studies to develop the first conceptual framework for PR in BD. The POETIC (Purpose and Meaning, Optimism and Hope, Empowerment, Tensions, Identity, Connectedness) framework, based on the CHIME (Connectedness, Hope and Optimism, Identity, Meaning and Purpose, Empowerment) framework [9], comprises the following processes: “Purpose and meaning,” “Optimism and hope,” “Empowerment,” “Tensions,” “Identity,” and “Connectedness” (see Table 1).

**Table 1.** The POETIC^a^ framework [8]: lived experience of personal recovery in bipolar disorder.

First-level domains	Second-level categories
Purpose and meaning	Meaning of mental illness experiences Paid or voluntary work Quality of life Meaningful life and social roles
Optimism and hope	Belief in possibility of recovery Positive thinking and valuing success Hope-inspiring relationships Having dreams and aspirations
Empowerment	Self-management and personal responsibility Controversial role of medication Control over life
Tensions	Balancing acceptance with ambitions Openness enables support but also stigmatization Ambivalence around (hypo)mania
Identity	Rebuilding positive sense of self Overcoming stigma Dimensions of identity
Connectedness	Support from others Relationships Peer support and support groups Being part of the community

a POETIC: Purpose and Meaning, Optimism and Hope, Empowerment, Tensions, Identify, Connectedness.

Current research on PR in BD has several limitations. First, it is mainly based on qualitative studies with few participants [10] and expert opinions, lacking quantitative evidence from larger samples [11]. Second, data collection is limited to structured settings (semistructured interviews, focus groups, and structured measures), which are not naturalistic and are subject to either interviewer bias [12] or constrained responses in structured measures. Third, recruitment is biased toward people who want to talk about PR and are in contact with services or researchers [8].

Naturalistic data collection, where “participants are not aware that they are being studied” [13], overcomes many of these limitations. Online forum posts are a source of naturalistic data, which can offer potential insights into “an experience as it is lived rather than as it is enacted in the researcher constructed environment” [14]. Some natural language processing (NLP) studies have analyzed large numbers of BD online forum posts via automatic quantitative methods such as content analysis [15] or emotion analysis [16,17] to identify forum topics or language differences between people with different or no MH diagnoses. Qualitative studies have applied conversation analysis [18], thematic analysis [19], grounded theory [20], and content analysis [21] to BD online forum posts. Such studies offer rich nuanced accounts of web-based discussions on BD but include only few, often handpicked, posts.

Corpus linguistics [22] provides a mix of quantitative and qualitative methods informed by linguistic theory for analyzing large amounts of text data with depth and richness that can overcome some of the shortcomings of previous NLP and qualitative studies. Semino et al [23] analyzed interviews and online forum posts of patients with cancer and their carers to learn about their lived experience and the metaphors they use for dealing with cancer. Hunt and Brookes [24] applied a combination of corpus linguistics and discourse analysis [25] to MH forum posts. Two corpus-linguistics studies have focused on BD specifically: Abdo et al [26] studied linguistic types of judgments, and McDonald and Woodward-Kron [27] studied forum users’ roles and identities.

A systematic review strongly recommended considering web-based content from individuals with lived experience in PR research [10], which has not yet been done. Therefore, the main aim of this paper was to gain further insights into the experience of PR in BD from online forum posts via a combination of NLP, corpus linguistics, and qualitative health research methods. Furthermore, the POETIC framework, synthesized from data collected via interviews or focus groups, has not been applied to new data yet. Hence, the secondary aim of this paper was to validate the framework by exploring to what extent it captures experiences shared on the web. The research question covering both aims is “What can online support forum posts reveal about the experience of PR in BD in relation to the POETIC framework?”

## Methods

### Data Source

This study analyzed posts from the international web-based discussion platform Reddit [28], which hosts subforums (subreddits) for various topics, including BD. Several reasons motivated the choice of this site: Reddit is one of the most visited internet sites worldwide with an international user base [29]; in contrast to other online support communities, everyone can read all public posts without a user account; and Reddit allows data analysis by third parties.

**Figure 1.**
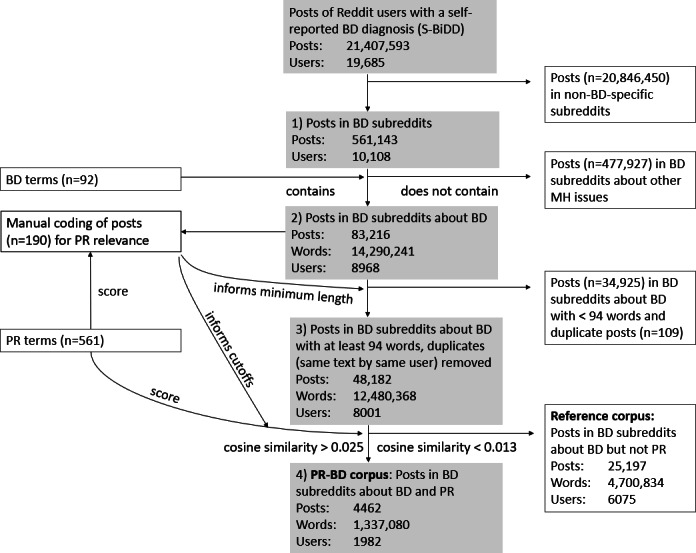
Flowchart of the 4 steps to create the PR-BD corpus and reference corpus. BD: bipolar disorder; MH: mental health; PR: personal recovery; S-BiDD: self-reported bipolar disorder diagnosis.

Reddit users with a self-reported BD diagnosis (S-BiDD) were automatically identified by matching phrases such as “I was diagnosed with bipolar” in all posts between January 2005 (the inception of Reddit) and March 2019 (see Jagfeld et al [30]). All posts of the identified users form the S-BiDD data set. Naturalistic data collection required subsequent filtering for content relevant for PR in BD, as an exploratory study revealed that the posts in the S-BiDD data set covered many other topics (see Report S1 in Multimedia Appendix 1). Figure 1 displays a flow chart for construction of the PR-BD corpus. In linguistics, a corpus is a sampled collection of texts representing a particular language variety [31]. The basis for the corpus was only posts in BD subreddits [32] (fourth level=“bipolar”), because a second exploratory study found that references to “recovery” and associated word forms were almost exclusively in relation to BD in BD subreddits (see Report S2 in Multimedia Appendix 1). Furthermore, only posts mentioning BD [33] were selected because only two-thirds (66%) of MH-related “recovery” mentions in BD subreddits referred to BD.

To select PR-relevant posts, a list of PR terms (comprising both single words and multiword phrases; n=562) [34] was compiled using corpus-linguistics methods (Document S2 in Multimedia Appendix 1). BD subreddit posts that mentioned BD were ranked according to their similarity with the PR terms list via term frequency–inverse document frequency–weighted cosine similarity, a standard information retrieval approach [35,36] (see Document S3 in Multimedia Appendix 1). To determine the cosine similarity cutoff, GJ coded whether 90 posts pertained to PR in BD using a preliminary codebook based on the second exploratory study and the POETIC codebook. SHJ audited the coding. To select the 90 posts, 10 posts were randomly sampled from every 10% quantile of the cosine similarity scores, taking only 10 posts from the first 2 quantiles that all scored 0. Following this, a minimum length of 94 words was set, as 5 posts shorter than this length lacked context to decide on their PR relevance (see Figure S5 in Multimedia Appendix 1). The codebook was refined to its final version (Document S4 in Multimedia Appendix 1). GJ and CH blindly coded 120 additional posts, again randomly sampled from each quantile of the cosine scores.

### Ethical Considerations

The Lancaster University Faculty of Health and Medicine research ethics committee approved this research in May 2019 (reference FHMREC18066), which follows ethics guidelines for internet-mediated research [37]. It was infeasible to seek individual informed consent from the large number of included forum users, but quotes were paraphrased to protect users’ anonymity (see Document S5 in Multimedia Appendix 1). We recognize that some people may object to the use of web-based posts as research data without individual consent (eg, [38]). Users generally post to share information or seek support and do not directly provide their content for research. However, we believe that on balance, the benefits of this research to better understand PR makes it worthwhile while acknowledging these potential objections.

The authors assert that all procedures contributing to this work comply with the ethical standards of the relevant national and institutional committees on human experimentation and with the Helsinki Declaration of 1975, as revised in 2008.

### Involvement of People With Lived Experience

In all, 4 volunteers with lived experience of BD, who use online forums, and were recruited via People in Research [39] provided input on the study design, results, and subsequent plans in individual web-based meetings. All 4 volunteers—1 man and 3 women—were UK-based and in their 30s to 40s; additionally, 3 (75%) reported a bipolar II disorder diagnosis, 1 (25%) did not further specify their BD diagnosis, and at least 2 (50%) had a migrant background. Importantly, all volunteers were very supportive of the project, and none raised ethical concerns. After study completion, the volunteers were reinvited to provide feedback on our interpretations of the results.

### Reflexivity

Reflexivity is important to highlight how subjectivity may have impacted on research findings [40]. The research team embraces a PR approach in BD. GJ, FL, and SHJ previously developed the POETIC framework for PR in BD. They anticipated that it would capture many aspects shared on the web, but data analysis would reveal new aspects and deeper insights into the experience of PR in everyday life.

### Corpus Framework Analysis

Data analysis drew on methods from corpus linguistics [22] and qualitative framework analysis [41], which we call corpus framework analysis. Quantitative corpus-linguistics methods derive frequency lists of the words in the corpus; identify keywords that occur statistically significantly more frequently in the corpus compared to other language samples; and find collocations, that is, words a target word co-occurs with more frequently than by chance. The main qualitative method is to analyze the context of specific words or phrases in so called concordances. Key lemmas in the PR-BD corpus were identified by comparing it to a reference corpus of posts with low similarity to the PR terms list via #LancsBox (version 6.0; Lancaster University) [42]. A lemma is the dictionary form of a word; for example, “recovering” and “recovered” are word forms of the lemma “recover.” To focus on the most salient topics of the PR-BD corpus, key lemmas overused at least twice at a significance level of *P*<.0001 [43] and used by at least 5% of users were analyzed. See Document S6 in Multimedia Appendix 1 for methodological details.

The key lemmas were coded into the POETIC framework via concordance analysis. First, overall impressions of all concordances were noted after sorting them according to the lemma, left, and right context (20 words each) in #LancsBox. Subsequently, 30 randomly sampled concordances for each key lemma were coded into the second-level POETIC categories (see the codebook in Appendix B of Jagfeld et al [8]). The coders read the full post if the 40 words did not provide enough context and noted impressions for every key lemma again. Finally, concordances that did not fit into an existing POETIC category were coded inductively. GJ coded all key lemmas, and SHJ and FL audited 6 key lemmas each.

Finally, new key lemmas that were not in the PR terms list and absent PR terms [44] were analyzed. Absence was defined as 0 frequency in the PR-BD corpus or a lower relative frequency than in the reference corpus. Additionally, collocations were analyzed via the #LancsBox *GraphColl* tool for some key lemmas. To do so, content words (noun, verb, adjective, and adverb) within a context of 5 words left and right of the target term and a minimum collocation frequency of 5 were ranked according to cubed mutual information [24].

## Results

The S-BiDD data set [45] contains 21,407,595 posts by 19,685 users (available for noncommercial research after signing a data usage agreement). The programming code is publicly available [46].

### Coding the PR Relevance of Posts and Constructing the PR-BD Corpus

Following a blind trial of coding the PR relevance of 20 posts and a subsequent discussion, GJ and CH achieved moderate agreement (Cohen κ=0.51; 77/100, 77% observed agreement) in coding the remaining 100 posts (see Table S20 in Multimedia Appendix 1). The team resolved all disagreements. In total, 66 (31%) of the 210 posts were coded as PR relevant (see Table S21 in Multimedia Appendix 1). Based on this, the PR-BD corpus comprises posts with a PR score above 0.025 to balance precision in selecting PR-relevant posts and corpus size (see Table S22 in Multimedia Appendix 1). The PR-BD corpus has 4462 posts with 1,337,080 words by 1982 users. The reference corpus of posts with a PR score below 0.013 (see Table S23 in Multimedia Appendix 1) comprises 25,197 posts with 4,700,834 words by 6075 users.

### Concordance Analysis With the POETIC Framework

**Figure 2.**
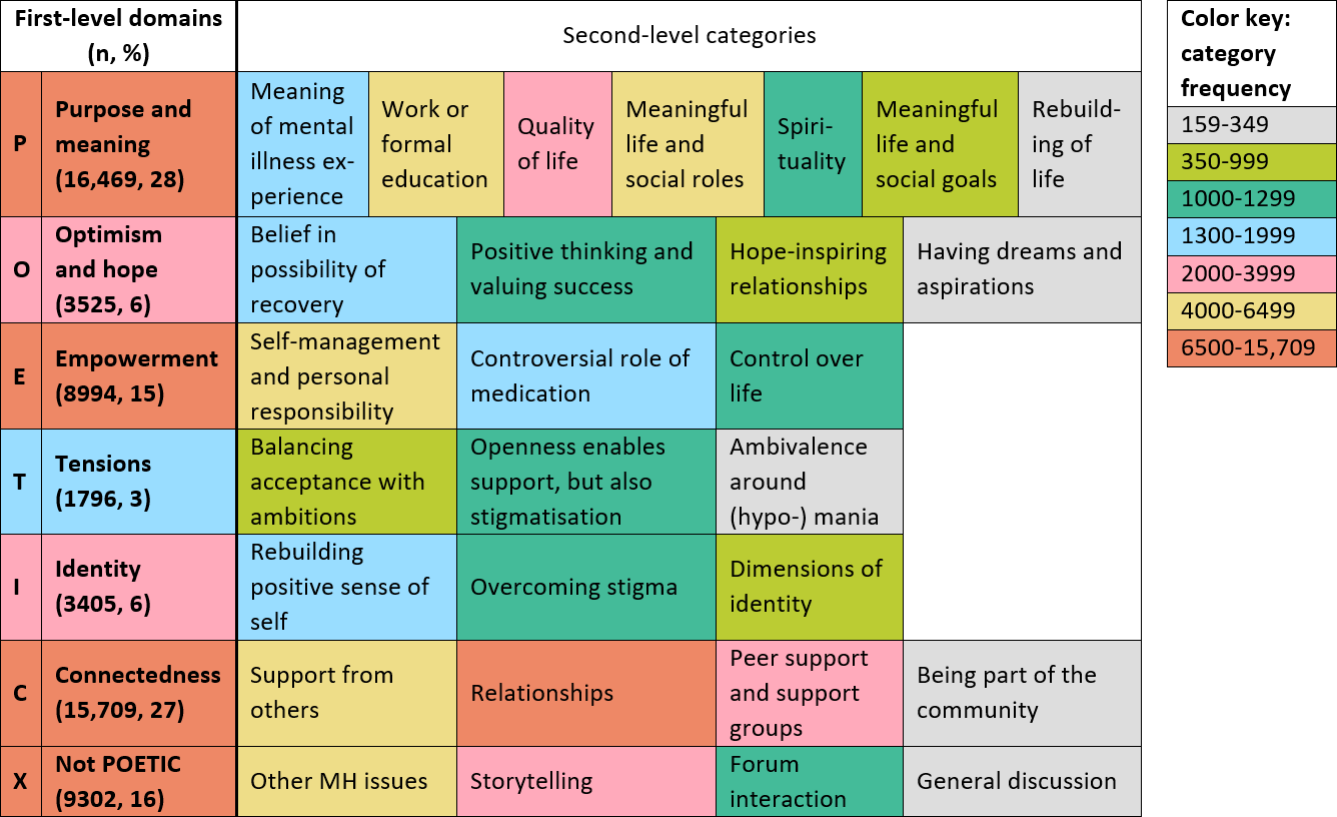
Frequency of POETIC domains and categories and new categories. MH: mental health; POETIC: Purpose and Meaning, Optimism and Hope, Empowerment, Tensions, Identity, Connectedness.

In all, 130 lemmas met the prespecified keyness criteria. Figure 2 shows the domain and category frequencies, extrapolated from the 30 concordance lines coded for each key lemma and color coded according to Tol’s [47] light scheme for color-blindness accessibility. Table S28 in Multimedia Appendix 1 lists the key lemmas coded into each category, and Table S29 in Multimedia Appendix 1 lists the categories that each key lemma was coded into. Overall, the POETIC framework captured the experiences in the PR-BD corpus very well: there was evidence for all categories. Only 16% (9303/59,199) of key lemma instances fell into the new “Not POETIC” domain rather than the existing framework. The text below briefly reviews each domain with key lemmas in italics, highlighting the differences between the original framework and the web-based data. Tables S30 and S31 in Multimedia Appendix 1 provide illustrative quotes for all categories.

### Purpose and Meaning

“Purpose and meaning” was the most frequent domain and contained the most salient topic differences between the POETIC review and web-based data. Although participants in the POETIC review mainly discussed the meaningful life and social role of being a *parent*, web-based discussions focused on reproductive decisions. Participants discussed perceived *risks* they might be *responsible* for, for example, the *possibility* for their *child* to *develop* MH issues that affected their *decision* or *choice* to *bear* and *raise kids*. In the “Work” category, extended to include formal education, many discussions focused on *struggles* around *studying* and *graduating college*. No participant in the POETIC review reported financial or housing issues, whereas several web-based users complained about a low *quality* of *life* due to *money* problems, causing homelessness or inability to afford treatment. Spirituality was discussed more frequently and richer than in the POETIC review. Users often wondered whether to regard their experiences as truly spiritual or rather as (hypo)manic symptoms.

### Optimism and Hope

Reddit users differed in their “Belief” in the possibility of *recovery*. The mainstream opinion on Reddit was that BD is a “chronic condition that cannot be *cured*, only *managed.*” Users questioned whether feeling fully *recovered* was not just a temporary experience caused by (hypo)mania. In the “*Positive* thinking and valuing *success*” category, many users were *grateful* for aspects of their BD experiences; for example, *challenges* provide *opportunities* for *growth* and demonstrating *strength*.

### Empowerment

As in the POETIC review, “Self-management and personal *responsibility*” was the most frequent and richest category. Forum users generally considered (taking *steps* towards) *maintaining* a *healthy lifestyle* (including *routines* or *schedules*, *diet*, *exercise*, and *coping skills*) as an individual’s *responsibility* to reach *recovery*. In contrast, experiencing MH symptoms or feeling stuck in their recovery was regarded outside of someone’s responsibility if they followed professional or mainstream forum advice. The “Controversial role of medication” category included concerns about drug effects on the *baby* during pregnancy or nursing and alternative non–evidence-based treatments such as the keto *diet* or cannabis, which were not present in the POETIC review.

### Tensions

Experiences coded in the “Tensions” domain were similar to the POETIC review. Several participants shared feeling more comfortable to discuss “Ambivalence” around (hypo)mania on the web. Some asked if there was a *possibility* to *enjoy* increased *motivation* and *confidence* to make *progress* in their goals without the hypomania getting out of control.

### Identity

Some participants shared rich *success* stories in the “Rebuilding positive sense of self” category, in which they moved away from *shame* and *guilt* by *forgiving* themselves for past behaviors and toward *accepting* themselves, whereas others were struggling with this process. *Shame* associated with *stigma* in the *society* was another focus of discussions, and some participants shared creative ways for overcoming stigma.

### Connectedness

Regarding “Connectedness,” users mainly discussed relationships and *support* from others. Although there were positive accounts, participants often discussed *struggles* with romantic *relationships* or *marriage* and *friendships* and complained about issues with *professional* and *family support,* similar to the POETIC review. However, the web-based accounts, particularly those of relationship and family problems, appeared more candid, for example, with them also discussing *sexual* issues, *trauma,* and *shame*.

### Not POETIC

Inductive coding of the 645 concordance lines that did not fit into the POETIC framework revealed that they were unrelated to individuals’ PR or lived experience. Most quotes discussed other MH issues without PR relevance (symptoms, genetics and heredity, treatment, diagnosis, societal issues, and scientific research), followed by storytelling of their own or others’ situation without PR relevance; direct interactions between forum users, for example, giving advice or congratulating; and discussions of non-MH issues.

### New PR Terms

Although 99 (76%) of 130 key lemmas were PR terms, 31 (24%) key lemmas were new. Of these 31 lemmas, 15 (48%) conveyed similar meanings to PR terms; for example, *brother* likened other family members in the PR terms list such as son or nephew. Another 7 (23%) new key lemmas introduced aspects not covered by PR terms. For example, *baby*, *raise*, and *bear* were related to reproductive decision-making; *childhood* was related to making sense of MH issues via early traumatic experiences; and *environment* was related to a focus on structural or societal circumstances rather than the individual (see Table S32 in Multimedia Appendix 1).

### Absent PR Terms

Only 13% (n=54) of the 416 unique PR terms (after removing spelling and phraseological variants) were absent: 46 (11%) did not appear in the PR-BD corpus and 8 (2%) were underused compared to the reference corpus. The underused PR terms referred to symptoms (*high mood*, *mania*, *manic*, and *sleep*) or medical MH professionals (*doctor*, *pdoc* [psychiatrist], and *psychiatrist*; see Table S33 in Multimedia Appendix 1). These terms were relevant for some PR domains but also were strongly associated with clinical recovery. All PR terms missing in the PR-BD corpus were also missing in the reference corpus. They were mostly complex phrases, for example, *brush yourself off*, and none indicated aspects that were not covered by other key lemmas (see Table S34 in Multimedia Appendix 1).

### Feedback From People With Lived Experience

Two volunteers who had commented on the first exploratory study provided feedback on the main study results. Overall, they valued the results and agreed with our findings but indicated limitations of the data, as reflected in the *Discussion* section. One volunteer argued that categorizations of experiences can be problematic for masking individual differences. Conversely, the other volunteer had found it particularly helpful to align some of her behaviors with CHIME categories because this gave her a sense of being on the right track.

## Discussion

This study analyzed Reddit posts of people with a BD diagnosis via corpus framework analysis to learn about the lived experience of PR in BD and validate the POETIC framework.

### Key Findings in Relationship to Previous Work

The primary study aim was to provide new insights on PR in BD. Indeed, the web-based data contained candid, in-the-moment experiences that traditional qualitative data collection is unlikely to retrieve. For example, 1 user posted about their experiences in a current manic episode on 2 subsequent days: “Yesterday I posted here about the realization that I’ve entered a manic episode.” Other users shared things on the web that they had not shared elsewhere: ‘Talking about this part of my inner world to a psychiatrist would require a lot of trust for me.” The users had different interpretations of elated mood as signs of recovery, spiritual experiences, helpful motivational boosts, or dangerous MH symptoms to avoid. Quantitative [48,49] and qualitative [50,51] evidence shows that web-based anonymity affords personal self-disclosures and discussions of sensitive and stigmatized issues.

The results show that 3 POETIC domains were featured the most in Reddit discussions: “Purpose and meaning” (particularly reproductive decision making, work, and formal education), “Connectedness” (romantic relationships and social support), and “Empowerment” (self-management and personal responsibility). In line with a recent quantitative review [52], the concerns raised on Reddit pointed to a wide range of social and occupational functioning among people with a BD diagnosis: some were not working or leaving their house and therefore sought support on the web, whereas others asked for specific advice to further improve their already functional lifestyle. The popularity of the “Self-management and personal responsibility” category agrees with recent quantitative findings. A review by Mezes et al [53] found positive associations between PR and psychological characteristics focusing on control and personal agency, and a longitudinal study identified positive impacts of adaptive coping and balanced risk-taking on PR [54].

Importantly, the analysis highlighted PR issues that exclusively or more frequently came up on the web. This might be due to differences in sample demographics and data collection methods between this study and those included in the POETIC review. Users in the S-BiDD data set were younger than those in the studies included in the POETIC review: the S-BiDD data set users had a mean age of 32 years versus 45 years in the POETIC review, 30% (5866/19,685) versus 17% (18/163) of participants were aged between 18-29 years, and 7% (1299/19,685) versus 34% (36/163) were aged between 50-64 years [8,30]. This might explain why perspectives on transitioning into adulthood with BD, challenges of college education, and reproductive decision-making exclusively surfaced in the web-based data. Sahota and Sankar [20] summarized their qualitative analysis of discussions of genetic risk and reproductive decision-making in 2 BD subreddits as centering around the manageability of parenting a child for people with a BD diagnosis, which aligns well with the experiences found in this study.

Moreover, users in the S-BiDD data set were overwhelmingly from the United States [30], whereas all POETIC review studies stemmed from countries that provide at least a basic level of free public MH care and social security (the United Kingdom, Norway, Australia, Canada, China, Spain, and Turkey). This may explain why existential financial issues such as (threat of) homelessness and the inability to afford treatment surfaced only in the web-based data. Since health insurance in the United States (except for Medicare for those aged 65+ y) is either employer provided or privately paid, individuals who cannot work due to their MH issues lose their insurance and in turn access to professional support, often causing MH issues to exacerbate, for example, by abruptly stopping medication. One Reddit user described this as a “vicious cycle.” It also appears plausible that Reddit users stem from a different socioeconomic group than the participants recruited into the POETIC review studies.

The secondary aim of this study was to validate the POETIC framework. Results confirmed that the framework usefully captured PR experiences shared on the web. Web-based users discussed all second-level POETIC categories, and only 645 of the 3900 analyzed concordance lines could not be accommodated in the framework, demonstrating its comprehensiveness.

### Strengths and Limitations

Three aspects of this study constitute both strengths and limitations. First, using online forums as a data source provided rich, candid, and in-the moment experiences. However, there is limited background and demographic information on the online forum users (but see Jagfeld et al [30] for an analysis of these properties in the users in the S-BiDD data set), and they are not representative of the general population with a BD diagnosis. One user in the PR-BD corpus posted “My hunch is that r/bipolarreddit overrepresents those who are struggling, who, understandably, may be more pessimistic about everything.” One volunteer shared his experience that discussions on Reddit MH forums mainly followed a mainstream opinion and that deviant opinions were ignored or suppressed. McDonald and Woodward-Kron [27] support this with corpus-linguistics evidence that BD forum users over time shifted from advice seeking to giving and used more medicalized language. Similarly, Vayreda and Antaki [18] showed that established BD forum users urged new members to seek a formal diagnosis and reinforced a biomedical view of BD. Our Reddit study provides one lens on the lived experience of some people that can complement studies of other MH forums and other sources, such as one-on-one interviews.

Second, the list of PR terms facilitated focusing on the concept of interest among the wealth of data, yet it arguably biased the data selection. Nevertheless, 52% (16/31) of the key lemmas that were not PR terms contributed new PR aspects. Moreover, explicitly stating our expectations of PR aspects via the terms list enabled us to identify absent aspects in the data.

Third, corpus-linguistics methods, particularly the coding of key lemmas, allowed the analysis of more data than traditional qualitative methods. However, single words probably more readily capture topic-like (eg, “Relationships”) rather than theme-like (eg, “Balancing acceptance with ambitions”) categories. Therefore, the relative category frequencies should be interpreted with some caution.

### Research Implications

This study has at least 4 research implications. First, it demonstrates the usefulness of analyzing online forum posts to tap into authentic and candid accounts of lived experience of MH issues. Second, this study serves as the first validation of the POETIC framework. Ideally, this encourages other researchers to apply it in their research. Third, the combination of corpus linguistics and qualitative framework analysis allowed the analysis of large amounts of data. Hence, corpus framework analysis may also be useful for future studies of text data, such as therapy transcripts (eg, [55]). Lastly, the S-BiDD data set and derived corpora are available for future research, for example, on other aspects of the lived experience of BD.

### Clinical Implications

This study identifies the key issues relevant to PR in BD shared by people with lived experience on the web and extends previous knowledge from interviews and focus groups. These findings, including the quotes in Tables S30 and S31 in Multimedia Appendix 1, are a rich resource for understanding more about the experience of PR in BD for individuals living with BD, their loved ones and informal carers, and MH professionals. This is also relevant for recent initiatives to educate MH professionals on the lived experience of severe MH issues, such as the current “Understanding psychosis and BD” training for the UK National Health Service [56]. Subsequently, issues identified in this study may provide helpful starting points for therapists to collaboratively consider them with their clients, for example, in recovery-focused therapy [57,58].

Individuals discussed issues on the web that they considered contentious and personal and were not comfortable sharing offline, such as sexuality, spirituality, and (hypo)mania. Recovery-focused therapies that are free to work with whatever model the clients bring for their BD experiences [58] may be particularly suitable to create a therapeutic environment where clients feel comfortable to discuss such sensitive issues. Moreover, Jones et al [59] showed that recovery-focused therapy reduces the positive self-appraisal of hypomanic experiences.

Reproductive decision-making surfaced as a major issue for young adults living with BD, and dedicated counseling on this topic may be advisable. Although understanding genetic vulnerability and risk data in MH is challenging, there is evidence that genetic counseling can offer effective support [60].

### Conclusions

This study analyzed 4462 Reddit posts by 1982 people with an S-BiDD within the POETIC framework [8] for PR in BD. It is the first to analyze online forum data on PR. This study confirmed the validity of the POETIC framework to also capture PR experiences shared on the web and highlighted new aspects in PR that did not come up in previous studies using interviews and focus groups. It also demonstrated the utility of integrating corpus linguistics and qualitative framework analysis to identify key themes within large text data sets. By indicating the key areas that people focus on when posting freely, this study provides rich insights into the lived experience of PR in BD for formal and informal carers of people with a BD diagnosis.

## Supplementary material

10.2196/46544 Multimedia Appendix 1 Supplementary reports and documents.

## Abbreviations

BD: bipolar disorder
CHIME: Connectedness, Hope and Optimism, Identity, Meaning and Purpose, Empowerment
MH: mental health
NLP: natural language processing
POETIC: Purpose and Meaning, Optimism and Hope, Empowerment, Tensions, Identity, Connectedness
PR: personal recovery
S-BiDD: self-reported bipolar disorder diagnosis
