# Supplementary material for: How People With a Bipolar Disorder Diagnosis Talk About Personal Recovery in Peer Online Support Forums: Corpus Framework Analysis Using the POETIC Framework
Source: JMIR Med Inform. 2023 Nov 8;11:e46544. doi: 10.2196/46544 (PMC10662676; doi:10.2196/46544)
Supplement: Multimedia Appendix 1 [file medinform-v11-e46544-s001.docx]

Report S1: Exploratory study 1: Main topics in BD subreddits

- 1. Summary

Exploratory study 1 investigated “What are the main topics that people with a self-reported bipolar disorder (BD) diagnosis talk about in BD online support forums?”. It consisted of an automatic content analysis of a 20M word corpus built from randomly sampled S-BiDD dataset posts in BD subreddits, followed by manual coding. The resulting topics in order of frequency were mental health (MH) symptoms, Professional treatment, Personal narratives, Recovery and self-management, and Recurrences and routines. MH symptoms and Professional treatment were ten times more frequent than all other topics.

- 1. Motivation

Content selection is necessary when working with non-reactive data as not all Reddit posts by people with a BD diagnosis state something about their PR experiences. However, understanding what issues beyond PR people with a BD diagnosis discuss on Reddit, helps to contextualise discussions about PR on this platform. In addition, a broad-scale content analysis of the online posts can determine how prevalent or ‘niche’ the topic is on the platform. Therefore, the first exploratory study investigated ‘What are the main topics and their relative frequency that people with a self-reported BD diagnosis talk about in BD subreddits?’

- 1. Methods

Figure S1 shows a flow chart of the corpus construction and analysis detailed in the following.


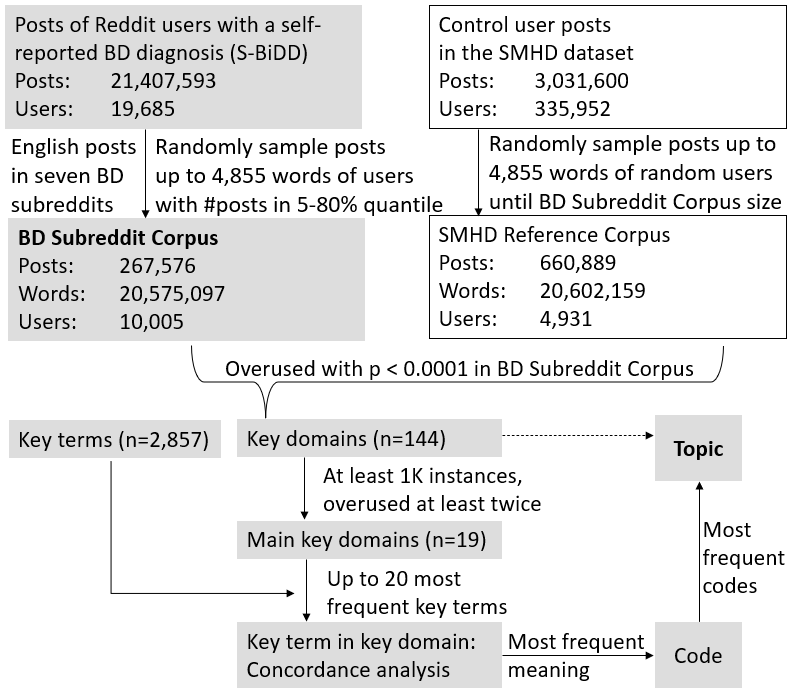


Figure S1 Corpus construction and analysis flow chart

- - 1. Corpus construction

The BD Subreddit Corpus constructed for this exploratory study consists of 20M words representative for English posts in seven BD subreddits^[[1]](#footnote-1)^ by people with a self-reported diagnosis of BD from randomly sampled dataset^[[2]](#footnote-2)^ posts. Precisely, the 5% of identified user accounts with fewest words in BD subreddits were removed (less than 76 words, 5% quantile) and posts of the 20% of user accounts who had posted most were downsampled until 4,855 words was reached or exceeded (80% quantile). Only English posts were analysed as 97% of the dataset posts are in this language as determined via the python-based automatic language detection system langid.py [1]. Wordcount was determined by tokenising posts with NLTK’s (version 3.4.1) TwitterTokenizer [2].

To determine key terms (words or phrases) and key semantic domains (see next subsection), a reference corpus was constructed from Reddit posts of the control users in the SMHD dataset by Cohan et al. [3]. These users never mentioned MH-related terms on Reddit or posted in MH-related subreddits. Up to 4,855 words of randomly sampled SMHD control users with at least 76 words were included until the SMHD Reference Corpus reached the same number of words as the BD Subreddit Corpus.

- - 1. Automatic content analysis via key semantic domains

The UCREL Semantic Analysis System (USAS) [4], an natural language processing tool for automatic content analysis, served to annotate words and phrases in the corpus with semantic domains. USAS assigns semantic domains to words and multi-word units using a manually created lexicon [5] and word sense disambiguation rules [4]. USAS requires input that is word-segmented (tokenised) and tagged with parts of speech by CLAWS [6]. USAS domains are hierarchically organised with 21 top-level domains, e.g., ‘Food and farming’, ‘Psychological actions, states, and processes’ (see Table S1) and 232 second- and third-level domains. Key terms (words and multiword phrases as identified by USAS) and key domains were identified by calculating which terms and USAS domains were statistically significantly overused^[[3]](#footnote-3)^ in the BD Subreddit Corpus compared to the SMHD Reference corpus.

Table S1 USAS top-level domains

| A: general and abstract terms | I: money and commerce in industry | Q: language and communication |
| --- | --- | --- |
| B: the body and the individual | K: entertainment, sports and games | S: social actions, states and processes |
| C: arts and crafts | L: life and living things | T: time |
| E: emotion | M: movement, location, travel and transport | W: world and environment |
| F: food and farming | N: numbers and measurement | X: psychological actions, states and processes |
| G: government and public | O: substances, materials, objects and equipment | Y: science and technology |
| H: architecture, housing and the home | P: education | Z: names and grammar |

- - 1. Manual analysis of key terms in key semantic domains

Since the USAS domain hierarchy intends to capture a general interpretation of the world, it can lack specificity for analysing corpora from a specialised domain [7]. Therefore, main key domains^[[4]](#footnote-4)^ were analysed in more detail following a standard corpus linguistic approach of keyword concordance analysis [7,8]. The contexts of the up to 20 most frequent key terms in each key domain were explored via the SketchEngine^[[5]](#footnote-5)^ concordance view. Based on this, codes were inductively assigned to reflect the explicit/surface meaning of the key terms in context and subsequently grouped into broader inductive domain-specific topics, following a conventional content analysis approach [9]. Each key term was assigned to one code based on its most frequent meaning and each USAS domain was assigned to one topic aggregated from the most frequent codes of the key terms tagged with this domain. This was difficult in some cases where key terms or words tagged with the same USAS domain referred to different aspects of living with BD, such as ‘mental health’ appearing predominantly in the context of ‘mental health symptoms’ but also in ‘mental health services’ (referring to treatment). Therefore, the topic frequencies represent approximations rather than exact values.

- 1. Results
     1. Corpus statistics

The BD Subreddit Corpus comprises 267,576 posts with a total of 20,575,097 words by 10,005 users. The SMHD Reference Corpus consists of 660,889 posts with a total of 20,602,159 words by 4,931 users (see Table S2). There are two striking differences: First, the vocabulary size in the reference corpus is almost three times as large as in the BD Subreddit Corpus. This is probably due to the more diverse range of topics expected in the reference corpus compared to the BD Subreddit Corpus that is restricted to a narrow set of subreddits. Second, posts in the BD Subreddit Corpus have more than twice the number of words than in the SMHD Reference Corpus. This aligns with Cohan *et al.* [3] who reported that the posts of all user groups with MH diagnoses, including BD were substantially longer compared to the posts of undiagnosed control users from which the SMHD Reference Corpus was sampled.

Table S2 BD Subreddit Corpus and SMHD Reference Corpus statistics. Vocabulary contains words and multi-word units as determined by USAS. Word counts based on NLTK tokenisation (SD = standard deviation)

| Corpus | Users | Vocabulary (hapax legomena) | Words | Posts | Mean +/- SD words/user | Mean +/- SD posts/user | Mean +/-SD words/post |
| --- | --- | --- | --- | --- | --- | --- | --- |
| BD sub-reddit corpus | 10,005 | 156,285 (58%) | 20,575,097 | 267,576 | 2,056 +/- 1,839 | 27 +/- 30 | 77 +/- 114 |
| SMHD Reference Corpus | 4,931 | 404,506 (66%) | 20,602,159 | 660,889 | 4,178 +/- 1,166 | 134 +/- 73 | 31 +/- 33 |

Figure S2 shows the number of words and Figure S3 the number of posts per user in the BD subreddit and SMHD Reference Corpus. The peaks in the number of users with around 5K words in both corpora are due to the sampling of posts for each user until 4,855 words were reached. Other than that, the data follow exponential distributions. This is in line with the 90-9-1 principle in online support forums which states that only 1% of superusers produce the majority of the forum content, while a further 9% of users supply the rest and the remaining 90% of users only observe the content without actively participating [10,11].


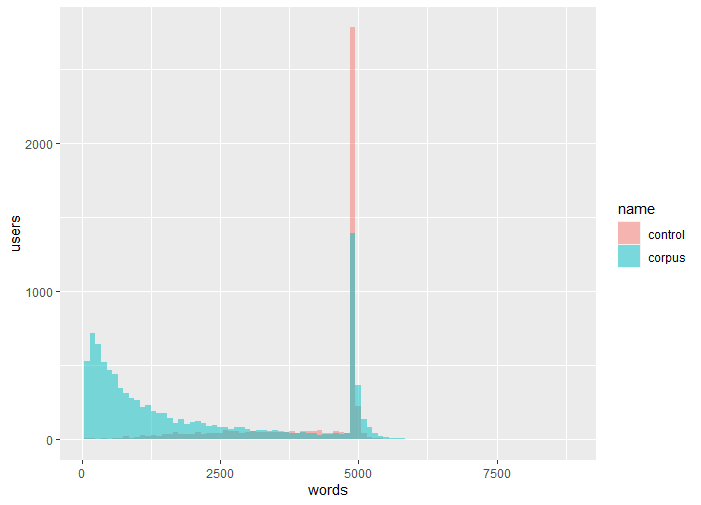


Figure S2 Words per user in the BD Subreddit Corpus (blue) and SMHD Reference Corpus (red)


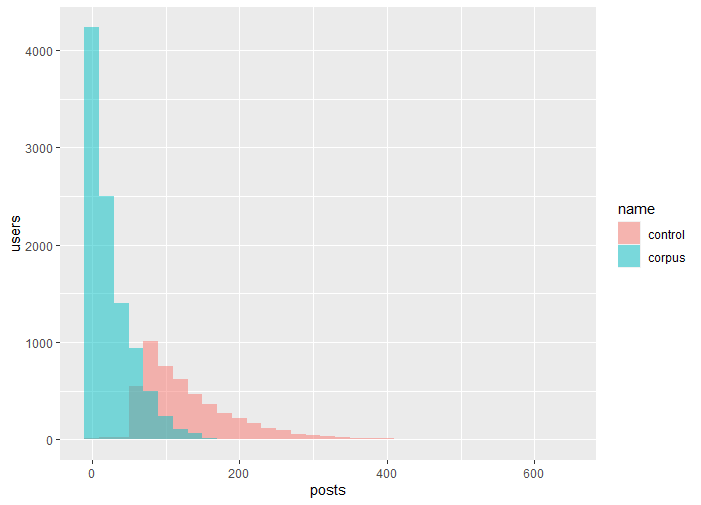


Figure S3 Number of posts per user in the BD Subreddit Corpus (blue) and SMHD Reference Corpus (red)

- - 1. Analysis of key terms in key semantic domains

Comparison of the BD Subreddit Corpus with the SMHD Reference Corpus yielded 3,201 key terms^[[6]](#footnote-6)^ and 141 USAS key domains^[[7]](#footnote-7)^ that were significantly overused in the BD Subreddit Corpus. Manual analysis of the most frequent key terms in the 19 main key domains resulted in five BD-related topics: MH symptoms, professional treatment, recovery and self-management, personal narratives, and recurrences and routines (see Table S3).

Table S3 BD-specific topic grouping and manually analysed key terms in the 19 main key domains in the BD Subreddit Corpus

| USAS domain | Frequency | Most frequent overused key terms (up to 20) |
| --- | --- | --- |
| **1. MH symptoms (420,697 mentions)** | | |
| **1.1. BD-specific (306,854 mentions)** | | |
| **1.1.1 Mania (181,432 mentions)** | | |
| B2- Disease | 154833 | mania (14110), disorder (11915), symptoms (10542), side effects (7338), crazy (5836), illness (5354), mental illness (4700), pain (4230), hurt (3809), sick (3741), adhd (3716), psychotic (3049), psychosis (2975), hypo (2255), side effect (1970), hallucinations (1894), paranoid (1880), disease (1815), paranoia (1759), patients (1549) |
| X5.2++ Interest/  energy/ excitedness | 24684 | manic (23,061), obsessive (588), addict (237), obsessively (195), fixated (88), on the manic side (29), manics (20) |
| A1.3- Lack of caution | 1915 | impulsive (925), reckless (410), rash (196), impulsively (175) |
| **1.1.2 Depression (77,356 mentions)** | | |
| E4.1- Sad | 77356 | depression (24772), depressed (14809), depressive (6885), sad (3827), cry (2334), upset (1953), crying (1790), suffering (1389), in a state (1227), regret (1160),miserable (1111), trauma (851), cried (849), sadness (767), embarrassed (642), suffered (599), embarrassing (592), depressions (524) |
| **1.1.3 Extreme mood and mood swings (48,066 mentions)** | | |
| E1 Emotional actions, states, processes | 35880 | mood (13462), moods (4007), feel (3889), emotions (3658), emotional (2863), mood swings (1672), moody (353), compassion (304), vibes, affective (199), tone (197), temper (178), self-control (151) |
| A6.3+ Comparing: variety | 8856 | mixed (3722), combination (1620), spectrum (785), erratic (274), combinations (235) |
| N5.1+++ Entirety: maximum | 3330 | extreme (2705), extremes (330), as much as possible (264) |
| **1.2 Other MH issues (78,968 mentions)** | | |
| E6- Apprehension/  confidence | 49169 | anxiety (13863), care (3869), stress (3545), worried (3177), anxious (3177), worry (2876), trouble (2404), PTSD (1746), concerned (1353), nervous (1232), stressful (1180), concern (831), concerns (798), worrying (546), caring (529), worries (323), dysphoric (319) |
| E5- Trepidation/  courage/ surprise | 29799 | psych (5645), scared (4118), fear (2482), scary (2205), afraid (2124), terrified (1445), panic (1166), terrifying (657), freaking out (598), scares (544), freaked out (472), freak out (471), fears (424), alarm (362), dread (306) |
| **1.3 General (25,350 mentions)** | | |
| A5.1-- Evaluation: bad | 9525 | worse (9100), exacerbated (130), exacerbate (119), exacerbates (67), exacerbating (55) |
| B2 Health and disease | 9114 | mental health (5674), health (2647), Medicaid (160), wellness (158), asymptomatic (29) |
| X1 Psychological actions/states/ processes | 6711 | mind (4831), psychological (419), sanity (326), state of mind (314), anhedonia (146), psychologically (91) |
| **2. Professional treatment (208,346 mentions)** | | |
| B3 Medicines and medical treatment | 208346 | diagnosed (20740), doctor (17524), medication (17498), psychiatrist (12949), diagnosis (11955), therapist (9910), therapy (8953), hospital (6837), treatment (6450), medications (5657), drugs (5362), doctors (4539), drug (4193), prescribed (3900), medical (3245), medicine (3169), pills (3002), medicated (2507), DR (2352), antidepressants (2190) |
| **3. Personal narratives (34,925 mentions)** | | |
| L1+ Life and living things | 34925 | life (31481), alive (1473), lives (1297) |
| **4. Recovery and self-management (34,583 mentions)** | | |
| A2.1- Affect: modify/change | 14216 | stable (7866), stability (1876), stabilize (600), stabilized (515), stabilizing (266), left alone (125), rut (104), stabilizes (72), stabilise (46) |
| X5.1+ Attention | 10295 | focus (4057), attention (1316), focused (811), focusing (601), Mindfulness (600), concentrate (576), concentration (454), mindful (292), concentrating (133), vigilant (119), acutely (47) |
| B2+ Health | 7753 | healthy (3620), recovery (908), recover (502), recovering (342), well being (184), recovered (184), well-being (103), snap out of it (124), wellbeing (103), healthily (56) |
| X9.2 Ability: success | 2319 | cope (1888), coped (66) |
| **5. Recurrences and routines (24,818 mentions)** | | |
| N6 Frequency/rate of recurrence | 24818 | sometimes (15822), at times (1417), on and off (598), n't ever (556), twice a day (520), once a week (449), once a day (275), once a month (270), off and on (145) |

While mentions of core BD symptoms (mania, depression, and extreme moods) were frequent key terms in the BD Subreddit Corpus, other frequent key terms referred to other MH concerns, mainly anxiety, addiction, and attention-deficit hyperactivity disorder (ADHD). The professional treatment topic consisted of the single USAS domain B3: “Medicines/medical treatment”. Based on the key term frequencies, medication(s)/meds (n=57,981 mentions) prescribed by a doctor (n=17,524) or psychiatrist (n=12,949) appear as the mostly discussed treatment, compared to far less mentions of (psycho-)therapy/therapies (n=9,246) offered by a therapist (n=9,910). The keyword “life” often appeared in personal narratives (‘for my whole life’).

Notably, posts in the BD Subreddit Corpus mentioned recovery and self-management far less frequently than MH symptoms and professional treatment. To illustrate this, the USAS domain B2: “Health and disease” distinguishes ill-health (B2-) and good health (B2+). B2- (n=154,833) contributed to the MH symptoms topic with terms such as mania, disorder, and symptoms. The twenty times less frequent B2+ domain (n=7,753) contributed to the Recovery and self-management topic with terms such as recovery and wellbeing. Temporal terms on frequency or rate of recurrence indicated routines, e.g., medication or self-care regimes.

- 1. Discussion

Via an automatic content analysis with subsequent manual analysis of key words, phrases, and content domains, this study found that people with a self-reported BD diagnosis most frequently talk about MH symptoms and professional treatment in BD subreddits. About ten times less frequently, they discuss their personal narratives, recovery and self-management, and recurrences and routines.

- - 1. Involvement of people with lived experience

After completion of this exploratory study, five volunteers with lived experience of BD and using online support forums recruited via PeopleInResearch were interviewed in June-July 2021. They confirmed the finding that the focus in BD online support forums is on symptoms and professional treatment, although one volunteer was surprised by how few mentions there were of positive experiences and recovery. All volunteers were familiar with the term recovery in relation to BD, but they had their personal preferences about how to talk about living with BD and recovery, using a variety of other terms, e.g., “living a normal life”, “feeling better”, “improvement”. All volunteers were very supportive of the research, and none raised ethical concerns.

- - 1. Limitations

While the automatic content analysis approach taken in this study allowed to analyse as many as 20M of words, two limitations of the USAS tool need to be pointed out. First, like virtually any automatic tool, USAS can make mistakes. USAS achieved 91% precision on general topic conversation transcripts [4], but this does not necessarily translate to online posts in the specific MH domain. However, the concordance analysis of key terms in the main key domains did not reveal frequent miscategorisations.

Second, since USAS is based on static dictionaries, it cannot assign a semantic domain to words that are not in its dictionaries. In the BD Subreddit Corpus, USAS could not assign a semantic domain to only 1.87% of term instances. GJ examined the 66 untagged terms with a frequency of at least 500 instances (accounting for 37.64% of untagged tokens) and proposed suitable USAS categories. USAS developers Paul Rayson and Andrew Wilson checked these assignments, which can be used to extend the USAS dictionary^[[8]](#footnote-8)^. The analysis revealed that most of them were psychotropic drug brand names (e.g., Lamictal, Seroquel, Latuda). Other frequently untagged instances were medication dosages that were not properly segmented by the tokeniser (e.g., 100mg). Few instances were diagnosis abbreviations, e.g., bp2, BPD, and Reddit-specific terms such as Reddit, subreddit. Most of the examined untagged words would fall into the USAS domains B3 (Medical treatment) and B2- (Disease), which were the two most frequent domains in the BD Subreddit Corpus already. Therefore, it can be confidently concluded that incomplete coverage of the USAS tool did not distort the results of this study and that re-tagging the corpora with complete coverage would likely not change the conclusions.

1. Report S2: Exploratory study 2: Usage of *recover* terms by people with a BD diagnosis
   1. Summary

Exploratory study 2 examined how Reddit users with a self-reported BD diagnosis use *recover* terms. Three coders coded the meaning of 377 instances of the eight most frequent word forms of recovery (i.e., recovery, recover, … = *recover* terms) in the *recover* corpus consisting of 57K S-BiDD dataset posts with at least one *recover* term. Overall, 43% of the instances denoted MH recovery, 23% recovery from physical health issues, 14% recovery from difficult life events, and 20% other meanings. Of the MH recovery instances, 66% described clinical recovery, whereas only 17% clearly denoted PR. Only 7.4% *recover* term instances related to BD, another 7.4% to PR, and only 1.3% to PR in BD. This indicated that Reddit users with a self-reported BD diagnosis seem to more readily associate topics such as alcohol addiction or recovery from physical health issues with ‘recovery’, rather than PR in BD. Also, Reddit users with a BD diagnosis almost exclusively used *recover* terms in relation to BD in BD subreddits.

- 1. Motivation

Although the research literature and MH systems widely adopt the term ‘recovery’ [e.g., 12–14], Michalak et al. [15] and McCabe et al. [16], among others, criticise the term as ambiguous between a clinical recovery vs. PR understanding. Qualitative studies have evidenced diverse understandings of the term by people with lived experience of severe MH issues [16], and BD specifically [15].

Michalak et al. [15] identify “a lack of precision about what the term recovery represents and entails” as a challenge in clinician-client communication and conclude that a “one size fits all” approach may not be linguistically appropriate for describing outcomes in people with mental illness” [15]. To complement and extend the existing qualitative evidence, the second study will for the first time yield quantitative evidence on the question ‘With what meanings do Reddit users with a self-reported BD diagnosis use *recover* terms (i.e., recovery, (to) recover, recoverable)?’.

- 1. Methods
     1. Corpus construction

Table S4 displays the three steps that led to the *recover* corpus. Initially, English^[[9]](#footnote-9)^ posts that contained at least one term instance (token) matching *recover* (e.g., recovery, recovered, irrecoverable) were selected from the S-BiDD dataset^[[10]](#footnote-10)^. Although *recover* appeared frequently as part of proper names, these deserve a separate study as the present research question focuses on lexical uses. Therefore, proper names (subreddit names, e.g., opiatesrecovery, edrecovery, user names and product names, e.g., MoistureCover, RecoverIT) were manually removed. Also, the matched term “precoverage” that did not have *recover* as stem was removed. This led to a whitelist of 98 *recover* content terms^[[11]](#footnote-11)^ to select posts in the second step. Finally, the number of posts for one user who had posted disproportionally many posts (n=3,997) (see Figure S4) was downsampled to the number of posts of the user with the second must number of posts (n=540) posts to create the *recover* corpus.

For manual coding, a random sample of 0.5% of the posts in the *recover* corpus was selected. Since only 15 *recover* term instances in the random sample appeared in BD-specific subreddits, 35 randomly selected instances from BD-specific subreddits were added. Additional randomly selected instances were added from the corpus for *recover* terms (see Section 2.3.2) that had less than five instances in the initial 0.5% sample.

Table S4 *recover* corpus creation

| Posts | Users | Subreddits | Sentences | Tokens | Unique *recover* terms |
| --- | --- | --- | --- | --- | --- |
| 1. All English S-BiDD dataset posts with at least one token that matches *recover* | | | | | |
| 61,553 | 9,119 | 4,125 | 795,911 | 12,786,617 | 241 |
| 1. After selecting only posts with at least one *recover* content term | | | | | |
| 60,716 | 9,098 | 4,097 | 787,893 | 12,668,640 | 150 |
| 1. *recover* corpus: after downsampling user with disproportionally many posts | | | | | |
| 57,259 | 9,098 | 4,087 | 755,220 | 12,190,378 | 145 |
| Posts from *recover* corpus for coding of *recover* instances | | | | | |
| 332 | 302 | 180 | 4,738 | 77,163 | 9 |


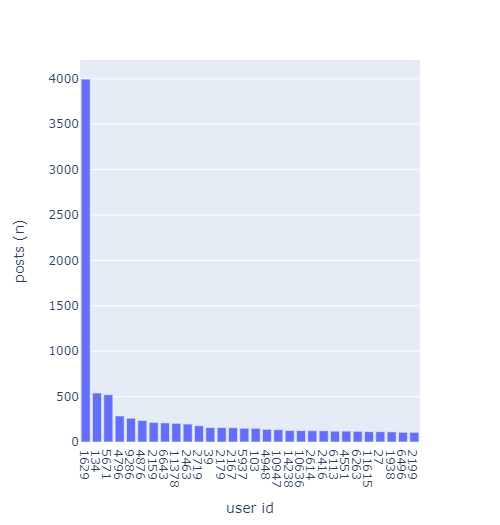

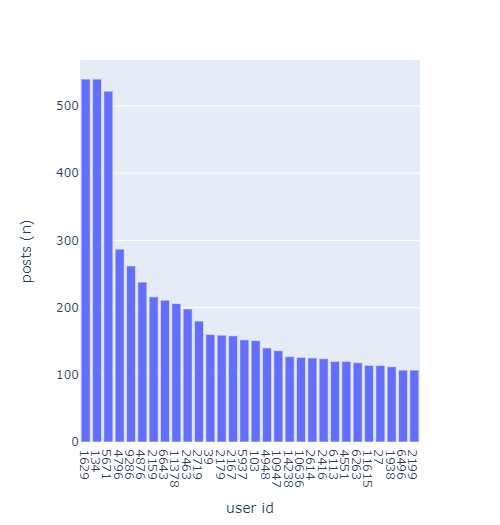


Figure S4 Number of posts that contain a *recover* content term for top 30 users before downsampling posts of user with id 1,626 (left) and afterwards (right)

- - 1. Manual coding

Eight *recover* terms that corresponded to standard English terms^[[12]](#footnote-12)^ and appeared at least 100 times in the corpus (see Table S6) were coded for their meaning. The codebook (see Document S1) consisted of the nine main categories shown in Table S10, including MH recovery, recovery from physical health issues, recovery from difficult live situations, and retrieving things. The study team agreed on these categories based on Oxford dictionary meanings of the *recover* terms, clinical definitions and operationalisations of personal and clinical recovery (i.e. the Questionnaire about the process of recovery [17]), and initial explorations of the data. For instances identified as denoting MH recovery coders additionally distinguished between PR and clinical recovery, the MH condition in relation to which the recover term was used, and whether the recovery was from a discrete MH episode or seen as long-term.

The coding spreadsheet presented each *recover* term instance and its sentence and post context. GJ coded all 377 instances. In a pilot to gauge feasibility of the task, PR coded 62 instances and SJ 29 of these. Subsequently, PR and SJ each coded half of the remaining 315 instances, such that at least two coders coded every instance. After agreement calculation, GJ considered the thread context from reddit.com (if available) if there was not enough information in the post alone. The coding team resolved all disagreements in discussion.

- 1. Results

Table S5 displays the top 10 subreddits in the S-BiDD dataset that contain most posts with a *recover* content token. Two are BD-specific (Bipolar, BipolarReddit), three are related to other MH issues (BPD, depression, raisedbynarcissists), two are about substance abuse (stopdrinking, OpiatesRecovery), and two are not MH-specific (AskReddit, relationships, Fitness).

Table S5 Top 10 subreddits with most posts in the *recover* corpus

| Subreddit | Posts in *recover* corpus (n) | *recover* corpus posts out of all subreddit posts (%) |
| --- | --- | --- |
| AskReddit | 5208 | 0.18 |
| bipolar | 2688 | 0.68 |
| stopdrinking | 2639 | 3.27 |
| BipolarReddit | 1293 | 0.93 |
| BPD | 1029 | 2.01 |
| relationships | 964 | 0.56 |
| OpiatesRecovery | 804 | 9.12 |
| raisedbynarcissists | 778 | 1.11 |
| depression | 654 | 0.89 |
| Fitness | 583 | 1.16 |

The *recover* corpus comprises 57,535 posts by 9,101 users in 4093 subreddits (758,037 sentences with 12,229,902 tokens). It contains 162 unique terms that match *recover*, of which 14 are standard English such as recovery, recovers, the remaining ones correspond to proper names that appeared in posts who also contained whitelisted *recover*content terms, e.g., redditorsinrecovery, spelling mistakes, e.g., recoverd, recoverying, or word segmentation issues, e.g., recovery](suburladdress (see Table S6).

Table S6 *recover* terms in the *recover* corpus with at least three instances (boldfaced terms are standard English terms), top eight terms selected for coding

| Rank | Term | Frequency | Rank | Term | Frequency |
| --- | --- | --- | --- | --- | --- |
| **1** | **recovery** | **32431** | 16 | recoverd | 10 |
| **2** | **recover** | **15460** | **17** | **unrecovered** | **8** |
| **3** | **recovering** | **10389** | 18 | edrecovery | 8 |
| **4** | **recovered** | **7171** | **19** | **recoverer** | **7** |
| **5** | **recovers** | **822** | **20** | **recoverability** | **6** |
| **6** | **recoveries** | **264** | 21 | soberrecovery.com | 5 |
| **7** | **recoverable** | **194** | 22 | recoverying | 5 |
| **8** | **unrecoverable** | **124** | 23 | recovery/ | 5 |
| 9 | recovery- | 34 | 24 | recover- | 5 |
| 10 | redditorsinrecovery | 31 | 25 | recover-- | 4 |
| **11** | **irrecoverable** | **23** | 26 | celebraterecovery | 4 |
| 12 | recovery](suburladdress | 19 | **27** | **recoverers** | **4** |
| 13 | opiatesrecovery | 17 | 28 | recovery-- | 3 |
| 14 | smartrecovery.org | 13 | 29 | dinosaursinrecovery | 3 |
| **15** | **irrecoverably** | **11** |  |  |  |

- - 1. Coding the meanings of *recover* terms

As Table S7 shows, both coder pairs achieved moderate to good agreement in coding the overall meaning of *recover* term instances (top left), and even good to very good agreement when only considering the instances where both coders assigned a definite code (not ‘Cannot determine’) (bottom left). In deciding whether *recover* term instances pertained to a PR or clinical recovery notion coders only reached a fair level of agreement even when only considering instances where they decided for one code or the other. Conversely, determining the MH issue for MH recovery instances seemed relatively uncontroversial and both coder pairs achieved good to very good agreement. In coding whether the *recover* term pertained to long-term recovery vs. recovery from an episode, one annotator pair achieved good agreement, while the other only agreed poorly.

Discussions of disagreements showed that particularly for ‘Recovery type’ the consistently low agreement for both coder pairs in the blind coding was due to two factors: First, inferring this information about the subjective state of the post author for an isolated instance of a *recover* term often lacked context. Some posts were very short and there was no opportunity to ask the author. This lack of context gave rise to diverging judgments based on the individual reading of the coder with their personal backgrounds. Importantly, most of the disagreements could be resolved in discussion. The team could not agree on the recovery type for only 16.7% of the MH recovery *recover* term instances (see ’Cannot determine’ in Table S10).

Table S7 Gwet’s AC_1_ (Gwet, 2014) and its standard error (SE), Cohen’s kappa (k) and observed agreement (P_0_) (see Table S8 for definitions) for the coding of the meaning of recover terms; benchmarking of AC_1_ and k (annotations and colour coding of cells) according to Altman’s (1991) scale (see Table S9)

|  | All | | | | Both coders agree on MH recovery | | | | | | | | | | | |
| --- | --- | --- | --- | --- | --- | --- | --- | --- | --- | --- | --- | --- | --- | --- | --- | --- |
|  | Meaning | | | | Recovery type | | | | MH issue | | | | Duration | | | |
| Coders | n | AC_1_, SE | k | P_0_ | n | AC_1_, SE | k | P_0_ | n | AC_1_, SE | k | P_0_ | n | AC_1_, SE | k | P_0_ |
| GJ + PR | 220 | 0.820, 0.028** | 0.78** | 0.836 | 85 | 0.335, 0.083 | 0.17 | 0.518 | 85 | 0.807, 0.045** | 0.77** | 0.824 | 85 | 0.268, 0.083 | 0.02^+^ | 0.459 |
| GJ + SJ | 186 | 0.623, 0.039* | 0.53* | 0.661 | 77 | 0.363, 0.086 | 0.09 | 0.519 | 77 | 0.840, 0.045** | 0.81*** | 0.857 | 77 | 0.703, 0.065* | 0.28^+^ | 0.753 |
|  | Both coders did not code ‘Cannot determine’ | | | | Both coders agree on MH recovery and did not code ‘Cannot determine’ | | | | | | | | | | | |
| GJ + PR | 203 | 0.885, 0.024*** | 0.85*** | 89.7 | 61 | 0.52, 0.11^+^ | 0.37^+^ | 72.1 | 71 | 0.89, 0.04*** | 0.86*** | 90.1 | 66 | 0.28, 0.13 | 0.03 | 57.6 |
| GJ + SJ | 159 | 0.746, 0.038** | 0.65** | 77.4 | 61 | 0.43 0.12^+^ | 0.18 | 65.6 | 63 | 0.928, 0.035*** | 0.91*** | 93.7 | 68 | 0.787, 0.068** | 0.34^+^ | 83.8 |

Table S8 Inter-rater agreement measures^[[13]](#footnote-13)^

| Name | Equation assumes two coders: coder 1, coder 2 total coded cases: N number of classes: z  Frequency that coder 1 assigned class i and coder 2 assigned class j: $f_{i, j}$  Frequency of coder 1 assigning class i: ${f_{i\cdot}= \sum_{j=1}^{z} f_{i,j}}$  Frequency of coder 2 assigning class i: $f_{\cdot i}= \sum_{j=1}^{z} f_{j,i}$ | Comment |
| --- | --- | --- |
| Raw agreement (P_0_) | $P_{0}= \frac{\sum_{i=1}^{z} f_{i, i}}{N}$ , $P_{0}\in[0,1]$ | Not corrected for chance agreement $P_{e}$, P_0_ *100 = % of agreed cases |
| Cohen’s kappa (k) [18] | $k= \frac{P_{0}- P_{e}}{1- P_{e}}$*,* $P_{e}= \frac{\sum_{i=1}^{z} f_{i\cdot}*f_{\cdot i}}{N^{2}}$  $k\in[-1,1]$ | Inaccurate for skewed and imbalanced label distributions [19] |
| Gwet’s AC_1_ [20] | ${AC}_{1}= \frac{P_{0}- P_{e}}{1- P_{e}}$  $P_{e}= \frac{\sum_{i=1}^{z} \pi_{i}* (1- \pi_{i})}{z-1}, \pi_{i}= \frac{f_{i\cdot}+f_{\cdot i}}{2}$  ${AC}_{1}\in[-1,1]$ | Recommended as most reliable inter-rater agreement measure for categories in discourse analysis by [21] and as more reliable than kappa in clinical research by [19] |

Table S9 Benchmarking of k and AC_1_ according to Altman’s (1991) scale. The k value directly maps to the agreement strength level where k ≥ lower value of the respective range. For AC_1_ and its standard error, the cumulative probability is calculated for each agreement strength level that the value falls into that category. The agreement strength level is selected as the most likely range to which the estimate belongs, when the cumulative probability crosses a certain threshold (Dettori and Norvell, 2020, p. 500). Following Gwet [22] this threshold was set to 95%.

| k/probability that the true agreement strength level of AC_1_, SE is the one associated with the value or one that is better is above 0.95 | Agreement strength level |
| --- | --- |
| 0.8-1 | Very good*** |
| 0.6-0.8 | Good** |
| 0.4-0.6 | Moderate* |
| 0.2-0.4 | Fair^+^ |
| <0.2 | Poor |

- - 1. Distribution of the meanings of *recover* terms

Table S10 shows the percentages of coded *recover* term instances in each of the categories overall and according to subreddit type (BD-specific, non-BD MH-focused, non-MH-related^[[14]](#footnote-14)^). As could be expected, Reddit users with a BD diagnosis more frequently refer to MH recovery with *recover* terms in subreddits specific to BD (80.0%) or other MH issues (84.0%) than in subreddits not related to MH issues (28.9%), where a physical health meaning is equally likely (28.5%). Still, even in MH specific subreddits, 16%-20% of *recover* term instances bear a non-MH-related meaning (recovery from physical health issues or difficult life events, or retrieving things). For MH recovery instances, a clinical recovery denotion is much more frequent (66.0%) compared to PR (17.3%), with little differences between subreddits with and without MH focus.

Table S10 Meanings of *recover* terms in Reddit posts of people with a self-reported BD diagnosis

| Category | Description | % total (n=377) | % BD subreddits (n=50) | % MH subreddits (n=50) | % non-MH subreddits (n=277) |
| --- | --- | --- | --- | --- | --- |
| **MH recovery** | Recovery from/in MH issues | **43.0** | **80.0** | **84.0** | **28.9** |
| - Clinical recovery (% MH recovery) | Measured by discrete, predefined, clinician-observed outcomes (e.g., symptoms + functioning) | 66.0 | 75.0 | 73.8 | 57.5 |
| - PR (% MH recovery) | Living a satisfying, hopeful, contributing life even with limitations of MH issues; self-defined, individual, process | **17.3** | **12.5** | **11.9** | **14.3** |
| - Cannot determine (% MH recovery) | Cannot distinguish between clinical recovery and PR | 16.7 | 12.5 | 14.3 | 20.0 |
| Physical health | Return to normal health/strength by overcoming a physical health issue (e.g., childbirth, surgery) | 22.8 | 12.0 | 2.0 | 28.5 |
| Life event | Overcoming a difficult life event/situation that did not cause a diagnosable MH issue (e.g., breakup, job loss) | 14.1 | 6.0 | 10.0 | 16.3 |
| Animal/plant | Animals or plants regaining health or strength | 2.7 | 0.0 | 0.0 | 3.6 |
| Inanimate | Inanimate agent (e.g., economy, PC operating system) regaining prosperity/resuming normal functioning | 3.7 | 0.0 | 0.0 | 5.1 |
| Retrieve | Regain possession or control of something stolen or lost | 8.0 | 0.0 | 4.0 | 10.1 |
| Energy | Remove/extract an energy source or industrial chemical for use, reuse, or waste treatment (e.g., sulphur recovery) | 0.3 | 0.0 | 0.0 | 0.4 |
| Proper noun | The *recover* term is (part of) a proper noun, e.g., Refuge Recovery | 1.9 | 0.0 | 0.0 | 2.5 |
| Other | Any other meaning or cannot determine meaning~~.~~ | 3.5 | 2.0 | 0.0 | 4.7 |

As Table S11 shows, Reddit users with a self-reported BD diagnosis are most likely to use *recover* term mentions in a MH recovery sense in relation to addiction (40.1%), predominantly alcohol. Only 17.3% of the instances refer to BD. As could be expected, a higher share (67.5%) of the instances refer to BD in BD subreddits. Interestingly, people with a BD diagnosis never used *recover* terms in relation to BD in non-BD-specific MH subreddits, but mainly in relation to addiction or eating disorders. More than a third of the MH recovery instances in BD subreddits were in relation to a specific MH episode, while almost all instances in other subreddits referred to longer term recovery.

Table S11 MH issues and duration of *recover* term instances with a MH recovery meaning

| Diagnosis | % all subreddits (n=162) | % BD subreddits (n=40) | MH subreddits (n=42) | non BD/MH subreddits (n=80) |
| --- | --- | --- | --- | --- |
| Addiction | 40.1 | 15.0 | 38.1 | 53.8 |
| - Alcohol (% of ADD) | 53.8 | 33.3 | 87.5 | 44.2 |
| - Other drugs (% of ADD) | 24.6 | 33.3 | 12.5 | 27.9 |
| - Other (multiple, sex) (% of ADD) | 21.5 | 33.3 | 0.0 | 27.9 |
| **BD** | **17.3** | **67.5** | **0.0** | **1.3** |
| Eating disorders | 14.2 | 0.0 | 28.6 | 13.8 |
| Major depressive disorder | 5.6 | 0.0 | 11.9 | 5.0 |
| Borderline personality disorder | 3.1 | 0.0 | 9.5 | 1.3 |
| Other (anxiety disorder, PTSD, psychotic disorder, self-harm, multiple, cannot determine) | 19.8 | 17.5 | 11.9 | 25.0 |
| **“Duration” of the MH recovery (whether from a discrete episode or long-term)** | | | | |
| Episode | 13.0 | 35.0 | 0.0 | 8.8 |
| Long-term | 74.1 | 52.5 | 90.5 | 76.3 |
| Cannot determine | 13.0 | 12.5 | 9.5 | 15.0 |

- 1. Discussion and limitations

The second exploratory study sought to determine with what meanings Reddit users with a self-reported BD diagnosis use *recover* terms. Overall, only 7.4% (n=28) *recover* term instances were in relation to BD and another 7.4% in relation to PR, and only 1.3% (n=5) in relation to PR in BD. This indicates that Reddit users with a self-reported BD diagnosis may more readily associate other topics with *recover* terms, such as alcohol addiction or recovery from physical health issues. The coding also showed that Reddit users with a BD diagnosis almost exclusively use *recover* terms in relation to BD in BD subreddits, but mostly in a clinical recovery sense. Therefore, the main study only considered posts in BD subreddits, but employed additional search terms and exclusion terms to select posts relevant to PR in BD.

There are two limitations of this study. First, the coders were part of the study team and therefore might have been implicitly biased in their coding by expectations of the outcome. However, this study was exploratory, so no hypotheses were tested or confirmed. Second, the interrater agreement was rather low for some subtasks, particularly for determining the underlying recovery notion of a *recover* term instance used in relation to MH issues. Importantly, the agreement rose to a fair level when omitting instances where at least one of the coders could not determine the recovery notion. Moreover, for all but 17% of the instances the team could agree on the recovery notion in the subsequent discussion. This indicates that inferring the recovery notion for a single *recover* term instance from the limited context of a post is difficult and not always possible.

Document S1: Codebook for meanings of *recover* terms

For each instance of a *recover* term, you are provided with its immediate context (the sentence in which it appears), the full post in which it appeared and the subreddit in which it was posted. See Table S12 for the coding sheet outline.

Table S12 Coding sheet outline

| Post ID | Sub- reddit | Term | Word number in sentence | Sentence | Post | Meaning | MH issue | Duration | Com- ment |
| --- | --- | --- | --- | --- | --- | --- | --- | --- | --- |
| 1 | Rabbits | recov-ered | 16 | I was trying to get her to move on all her four legs when she recovered. | [full post text – not shown here] | Animal/ plant |  |  |  |
| 2 | bipolar | recover | 23 | I’m currently recovering from my latest manic episode. | [full post text – not shown here] | Clinical recovery | BD | episode |  |

Based on only the information present in the coding sheet, please code the **meaning** of the *recover* term according to the codebook in Table S13 using the boldfaced codes in the first column. If there is more than one *recover* term in the post, please only code the meaning for the highlighted instance based on the sentence and the number of the term in the sentence.

If the *recover* term is used in relation to MH (**MH recovery**), please use the additional guidance in Table S14─S16 to distinguish **Clinical recovery and PR**. Please also specify all **MH issues** that the *recover* term is used in relation to according to Table S17.

For **Clinical recovery**, please specify whether the *recover* term is used in relation to a specific episode or for longer term recovery (**duration**) according to Table S18.

Please document the reasons for your decisions in the **comment** column.

Table S13 Codes for meanings of recovery, recover, recoverable, irrecoverable

| Code | Description | Example quotes (paraphrased excerpts from real posts or dictionary entries) | Code source |
| --- | --- | --- | --- |
| **Recovery in or from MH issues** | | | |
| **Clinical recovery** Clinical + socio-functional recovery in SMHIs | Clinical recovery consists of discrete outcomes that clinicians can observe objectively, such as current depressive, psychotic, or manic symptoms, vocational and social functioning, and service use and treatment aspects (e.g., time since last hospital admission, taking medication or being in psychotherapy) [23,24]; Can apply to an individual episode/relapse. | 1. r/Random_Acts_of_Amazon I was diagnosed with bipolar, PTSD and Anorexia a year ago. My boyfriend cheated on me with his ex partner on our anniversary. I self harmed every day, multiple times a day. Now, I am properly medicated for my mental health issues. I am still going through recovery for my eating disorder due to a recent relapse. For our 2 year anniversary, my boyfriend gave me a promise ring. I have also been free of self harm for 6 months! I am happy as my world has turned around!  2. r/neuroscience Can the brain recover from depression and does this change its structure? | Clinical / theoretical |
| **PR** PR in SMHIs | Recovery is a deeply personal, unique process of changing one’s attitudes, values, feelings, goals, skills and/or roles. It is a way of living a satisfying, hopeful and contributing life even with the limitations caused by the illness. Recovery involves the development of new meaning and purpose in one’s life as one grows beyond the catastrophic effects of mental illness” [25]. Broader scope than an individual episode/relapse. | 1. r/depression 4 years ago I was at my lowest and lost everything, but somehow I've managed to build a new life and I'm again considered 'successful'. The open wound of depression in my brain has healed to a scar. I don't need therapy or medication but I feel like I can't just erase that part of me. Does anyone have a recovery-from-depression stories from which I could learn how to deal with this?  2. I committed suicide and died but then was brought back in to a coma. This was such a slow and painful psychological recovery but now it’s been about 3 years and this has certainly shaped my life to enjoy everything in life and to make me one of the happiest people in the world. | Clinical / theoretical |
| **MH recovery** | Recovery in or from MH issues. Only assign if cannot distinguish between clinical recovery and PR | 1. I switched from college education to drug business for a few months. Luckily, I was able to recover and get my priorities straight and graduate, but for a time, I felt as though I'd given up my entire life to my dependence on the substances.  2. The first time I couldn’t continue working due to mental health issues, I took a medical leave. The second time, I just resigned. I have yet to recover. | Clinical/theoretical |
| **Regaining health or strength** | | | |
| **Life event**  Human recovering from difficult life event/situation | Overcoming a difficult life event/situation that didn’t cause a SMHI | 1. I’m still sort of recovering from the break up.  2. Recovering cookie-addict 😉 | Data |
| **Physical health** Human Clinical recovery from physical health issues | Return to normal state of health and strength by overcoming a physical health issue | 1. When I give my body rest and healthy food, it seems to recover quicker.  2. r/Casualima: How long was recovery after ECT treatment before you were back to yourself? | Clinical / theoretical |
| **Animal/plant** Recovery of animals, plants | Animals or plants regaining health or strenth | After the road accident, the family dog Barko is back at home and he looks all set to make a full recovery. | Theoretical |
| **Inanimate**  Recovery of inanimate objects or concepts | Economy, market, operating system (recovery mode) regaining prosperity/resuming normal functioning **Note: Only assign Inanimate if not Retrieve.** | 1. Make sure the device is turned off, then turn it on again. Upon startup, hold Cmd+R to boot into recovery mode. 2. r/SamandTolki: How did their marriage recover, or did it ever truly recover? 3. His reputation never recovered after lost his title. | Dictionary |
| **Other meanings** | | | |
| **Retrieve**  Retrieve / find again | The action or process of regaining possession or control of something stolen or lost, includes data/file recovery Test: You can replace “recover*” in the sentence with “retrieve*” or “regain*”. | 1. r/bipolar: I was hoping so much I could recover a small sliver of my sanity.  2. I have seen reps at my Apple Store actually refer customers out to third - party data recovery services. **Note: Retrieve takes precedence over ‘Inanimate’, even if the actor who is retrieving/regaining sth [here the service] is inanimate.** | Data / dictionary |
| **Energy**  recover energy/substances | The process of removing or extracting an energy source or industrial chemical for use, reuse, or waste treatment. (‘sulphur recovery’) | 1. While silver recovery systems remove most of the chemical, other dangerous solvents are used to clean film-developing machinery.  2. The draft document deals with waste minimisation, recycling and energy recovery. (https://www.lexico.com/en/definition/recovery) | Dictionary |
| **Proper noun** | The *recover* term forms part of a proper noun | Have you heard of Refuge Recovery? They don’t meet in person very often but the’ve also got phone meetings in addition. | Data |
| **Other**  Other/Unclear | Any other meaning not captured by the codes above or cannot determine meaning due to lack of context or meaning is ambiguous between codes; please specify |  |  |

Table S14 Criteria to distinguish clinical recovery and PR based on Slade (2009, p. 35)

| **Clinical recovery** | **Example** | **PR** | **Example** |
| --- | --- | --- | --- |
| Outcome or state, dichotomous | DSM-5/ICD-11 diagnosis specifiers for clinical recovery status: partial remission, full remission | Journey or process, on a continuous scale | The Bipolar Recovery Questionnaire (BRQ) measures self-reported responses on 10-cm visual analogue scales from strongly disagree (0) to strongly agree (100) |
| Observable, objective | One criterium for the reliability of scales for clinical recovery or socio-occupational functioning is how much independent clinicians agree when appraising the same individual [e.g., 26] | Subjective | See QPR questions in Table S15, where individuals self-define what “purpose” means in their lives (item 6) |
| Often rated by clinician (except for Patient reported outcome measures (PROMS)) |  | Self-reported |  |
| Definition invariant across individuals |  | individual, idiosyncratic meaning |  |

To enable a more structured distinction between clinical and PR, please check if the content in relation to the *recover* term in the post refers to aspects of any of the items of the Questionnaire about the process of recovery (QPR) listed in Table S15 and follow the decision guidance in Table S16. Assign only “MH recovery” in case you cannot determine whether the user uses *recover* in a clinical recovery or PR sense (please provide a comment in this case).

Table S15 Items in the Questionnaire of Personal Recovery [17]

| 1 | I feel better about myself |
| --- | --- |
| 2 | I feel able to take chances in life |
| 3 | I am able to develop positive relationships with other people |
| 4 | I feel part of society rather than isolated |
| 5 | I am able to assert myself |
| 6 | I feel that my life has a purpose |
| 7 | My experiences have changed me for the better |
| 8 | I have been able to come to terms with things that have happened to me in the past and move on with my life. |
| 9 | I am basically strongly motivated to get better. |
| 10 | I can recognise the positive things I have done. |
| 11 | I am able to understand myself better |
| 12 | I can take charge of my life. |
| 13 | I can actively engage with life |
| 14 | I can take control of aspects of my life |
| 15 | I can find the time to do the things I enjoy |

Table S16 Clinical recovery vs. PR code decision based on the QPR (all in relation to a specific post by a user)

| Item status | Example | Code |
| --- | --- | --- |
| User does not relate an aspect of any QPR item to recovery | 1. r/depression One meds combination made me feel SO GOOD Except I gained 20 pounds in a month. Nothing has worked as good as this combo. I can't imagine how I would act without meds, they're so important to recovery from depression.  2. As far as recovery goes, I'm on medication I hate but which works and I'm not in therapy. [recovery only seen as equivalent to treatment] | Clinical recovery (likely in absence of other evidence based on Table S14 criteria) |
| User indicates that they are not interested in at least one QPR item in relation to recovery / they indicate that at least one QPR item is not relevant to their view of recovery | I don’t cate about anything at this point in my life, I just want to recover from this crippling feeling of anxiety. [made this up as haven’t encountered example yet] | Clinical recovery (in absence of the user endorsing aspect(s) of at least one QPR item in relation to recovery) |
| User associates wish to do / hope to reach at least one QPR item with recovery | I’ve been very depressed for a long time but I really wish to recover and feel like my life has a purpose again. | PR |
| User indicates that they [or someone else] partly or fully feel / feel able to do / do / experience at least one QPR item and consider themselves [or this someone] in recovery | I committed suicide and died but then was brought back in to a coma. This was such a slow and painful psychological recovery but now it’s been about 3 years and this has certainly shaped my life to enjoy everything in life and to make me one of the happiest people in the world. [e.g. fulfils 10, 15] | PR |
| User indicates some aspects of QPR items in relation to recovery, but personal evaluation is unclear | r/BPD: CBT, DBT, meditation and mindfulness have helped me a lot. I've read about people in recovery from BPD. It isn’t impossible but it's damn hard work. [CBT, DBT, meditation and mindfulness may have positive effects for many QPR dimensions (e.g. QPR 11) but the author is not explicit in what ways they have helped them, could still be seen only in relation to BDP symptoms] | Unclear – label as “MH recovery” and provide comment |

Table S17 MH issues

| MH issue | Code | Examples/scope |
| --- | --- | --- |
| Addiction | ADD | Please specify type:  Alcohol -A Other drugs -D Sex -S Other (specify, e.g., gambling) |
| Anxiety disorder | AD | Social anxiety, panic disorder, generalised anxiety disorder (GAD), panic attacks |
| Attention deficit hyperactivity disorder | ADHD | ADHD |
| Autism spectrum disorder | ASD | Autism, Asperger syndrome |
| Bipolar disorder | BD | Any type (BD-1, BD-2, Bipolar spectrum disorder, cyclothymia) |
| Borderline personality disorder | BPD | Borderline |
| Eating disorder | ED | Anorexia, bulimia |
| Major depressive disorder | MDD | Depression, major depression, dysthymia |
| Obsessive-compulsive disorder | OCD | OCD, skin picking |
| Post-traumatic stress disorder | PTSD | PTSD |
| Psychotic disorder | PD | Schizophrenia, Schizoaffective disorder |
| Multiple | Multiple | MDD+AD, ED+MDD (comment) |
| Other | Other | Please specify (self-harm, suicidal ideation/attempt, other personality disorders, other MH diagnoses …) |

Note: depressive episodes can occur as part of both BD and MDD. Label instances of “recovery from depression” as concerning the MH issue BD if the user additionally mentions a BD diagnosis/BD symptoms (high mood, mixed mood) sin the post and as concerning MDD otherwise.

Table S18 Criteria to distinguish recovery from an episode vs. longer term recovery

| Type | Code | Example |
| --- | --- | --- |
| Recovery from an episode Mood episodes: depressed, (hypo-)manic Other episodes: psychotic | Episode | r/BipolarReddit: So far, I’ve had an 7 month break from depression, after 5 months of growing mania with fina eruption, and my 3 months of post-mania **recovery**  2. r/bipolar The recovery from mania is usually worse than recovering from depression. |
| Persistent symptom remission over a longer period of time, not mentioned in relation to a specific episode | Long-term | r/stopdrinking: In early recovery, I was more isolated. I became more willing to take risks and socialise with people (if alcohol was not the main focus) with more sober time under my belt. |
| Cannot determine whether meant in relation to an episode or long-term | Other |  |

Document S2: Construction of the PR terms list

Candidate PR terms were generated from four sources: First, articles included in the POETIC review (n=12, 75,553 words) were compared to excluded articles (n=80, 409,877 words) that fulfilled all inclusion criteria (qualitative study reporting only experiences of people with a BD diagnosis) except a PR focus. Keywords (log likelihood > 10.83, *P*<.0001) were identified by comparing the article texts of included vs. excluded studies via the corpus linguistic software tool Wmatrix v4 [27]. To do so, the full paper text without abstract, tables, figures, and appendices were converted from pdf to txt via Science Parse^[[15]](#footnote-15)^ with manual post-correction. Three excluded articles were only available as images and not included because an additional optical character recognition step was regarded as not time efficient.

Second, keywords for each POETIC domain were identified by comparing the quotes coded into each POETIC domain in the review to the quotes coded into all other domains (see Table S19). Third, keywords (log likelihood > 10.83, *P*<.0001) were identified in MH recovery posts from exploratory study 2 that only contained *recover* term instances coded as PR (n= 26, 6,368 words) by comparing them against posts that only contained *recover* term instances coded as clinical recovery (n=89, 13,790 words).

Table S19 Corpus sizes and keywords for quotes coded in each POETIC domain from articles included in the POETIC review,
LL = log likelihood

| Domain | Words | Words reference (coded in any other domain) | keywords (min freq 1, LL > 6.63 (*P*<.01) | Overused keywords (min freq 2, LL > 6.63) |
| --- | --- | --- | --- | --- |
| 1 Purpose and Meaning | 8237 | 33316 | 19 | 13 |
| 2 Optimism and hope | 2650 | 37021 | 25 | 19 |
| 3 Empowerment | 14968 | 26946 | 42 | 20 |
| 4 Tensions | 6490 | 33968 | 29 | 17 |
| 5 Identity | 5777 | 34724 | 22 | 16 |
| 6 Connectedness | 9221 | 32047 | 41 | 20 |

Third, the concordances of keywords that appeared in at least two included articles or PR posts by at least two users were manually examined in the corpus-linguistic software tool AntConc [28] to identify words and phrases indicative of PR. Using two different corpus linguistic tools was necessary because wmatrix does not allow to sort concordances of keywords according to their left and right context, while Antconc does not report the frequency of keywords in the reference corpus. All wordcounts are reported according to wmatrix.

Finally, the study team selected the terms indicative of PR from the candidates and extended the list with terms informed by their expert knowledge, and spelling and phraseological variants (e.g. self-esteem and self esteem, forgive yourself and forgive herself).

Document S3: Tf-idf weighted cosine similarity to score the PR relevance of Reddit posts

The following explanation of tf-idf weighted cosine similarity is according to [29].

Given a vocabulary^[[16]](#footnote-16)^ of size N with i representing one term of the vocabulary, a post p is represented by the vector x of length N according to its bag of words with one hot encoding:

$x_{i}= \left\{ \begin{aligned} 1 if vocabulary term i appears in p \\ 0 else \end{aligned} \right.$
Analogously, the PR terms are represented by the vector y, also of length N:

$y_{i}= \left\{ \begin{aligned} 1 if vocabulary term i appears in the PR terms list \\ 0 else \end{aligned} \right.$

The PR score of the post p is calculated as the cosine similarity between the tf-idf weighted vector representation $\bar{x}$ of the vector representation x and the tf-idf weighted vector representation $\bar{y}$ of the PR terms list vector representation y:

PR score (x) = $cosine\left( \bar{x},\bar{y} \right)= \frac{\sum_{i=1}^{N} \bar{x}_{i}*\bar{y}_{i}}{\sqrt{\sum_{i=1}^{N} \bar{x}_{i}^{2}}*\sqrt{\sum_{i=1}^{N} \bar{y}_{i}^{2}}}$

The tf-idf weighted vector representation $\bar{z}$ of a vector z and a particular post p is calculated by multiplying the vector entry for each vocabulary term i with its associated weight $w_{i, p}$: $\bar{z}=w_{i, p}* z_{i}$

The weights consist of the multiplication of the term frequency ${tf}_{i, p}$ of vocabulary term i and the inverse document frequency ${idf}_{i}$ of term i: $w_{i, p}= {tf}_{i, p}* {idf}_{i}$, where the frequency of term i that appears $count\left( i, p \right)$ times in a post p is defined as follows:

${tf}_{i,p}= \left\{ \begin{aligned} 1+ \log_{10} count(i, p) if count\left( i, p \right)>0 \\ 0 else \end{aligned} \right.$ and the inverse document frequency of term i that appears in ${df}_{i}$ posts out of a collection of M posts (traditionally called documents) is

${idf}_{i}= \log_{10} \frac{N}{{df}_{i}}$.


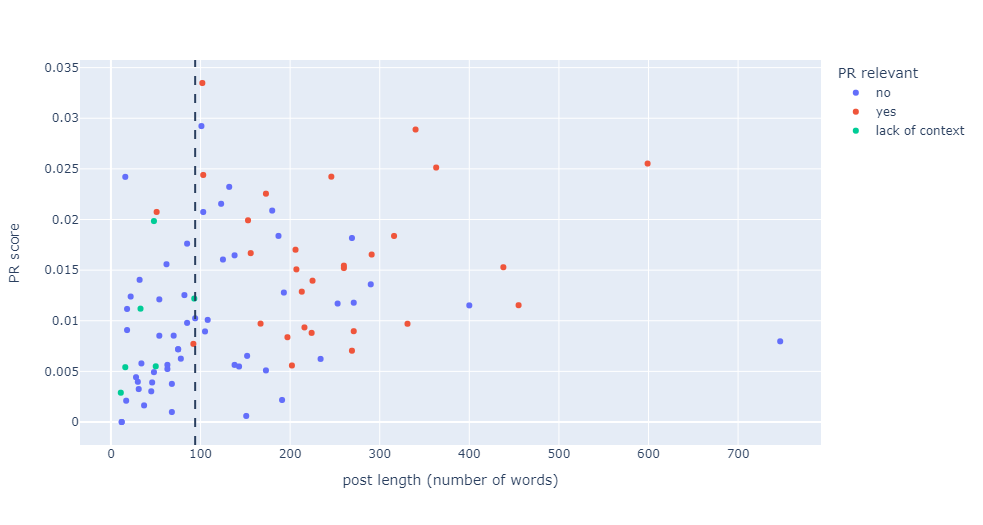


Figure S5 PR-relevance for 90 posts by length and cosine similarity score with the PR terms list, dashed line at 94 words is the maximum length of posts for which PR relevance could not be determined due to a lack of context; list of all 210 post ids with PR relevance codes^[[17]](#footnote-17)^

Table S20 Gwet’s AC_1_ (Gwet, 2014) and its standard error (SE), Cohen’s kappa (k) and observed agreement (P_0_) (see Table S8 for definitions) between GJ and CH for coding the PR relevant of posts; benchmarking of AC_1_ and k according to Altman’s (1991) scale: ^+^fair, *moderate, **good level of agreement (see Table S9)

|  | n | AC_1_, SE | k | P_0_ |
| --- | --- | --- | --- | --- |
| Pilot | 20 | 0.55^+^, 0.19 | 0.43* | 0.75 |
| Main | 100 | 0.57*, 0.08 | 0.51* | 0.77 |
| Main confident (both 3,4,5) | 62 | 0.77**, 0.08 | 0.71** | 0.87 |

Table S21 Coding results for PR relevance

| PR relevant | yes | no | Lack of context/cannot decide |
| --- | --- | --- | --- |
| 90 posts (GJ coded, SJ audited) | 29 | 55 | 6 |
| 120 posts (GJ + CH coded), >= 94 words | 37 | 78 | 5 |
| Total (% of 210) | 66 (31.4%) | 133 (63.3%) | 11 (5.2%) |

Table S22 Precision, recall and instances that were correctly included (true positives (TP)), incorrectly included (false positives (FP)), incorrectly excluded (false negatives (FN)), and corpus sizes for cutoffs of the PR terms similarity scores; selected cutoff boldfaced; wordcounts according to spacy tokenization – note that this differs from the corpus wordcount reported in the main text which is according to LancsBox / TreeTagger tokenisation

|  |  | Posts with at least 94 words (n= 167), coded as PR relevant (n= 64) or not (n= 103) | | | | | All posts (n= 83,216) | | |
| --- | --- | --- | --- | --- | --- | --- | --- | --- | --- |
| Quantile | Cutoff PR > x | Precision  TP/(TP+FP) | Recall TP/(TP+FN) | TP | FP | FN | posts | users | words |
|  | -1.000 | 0.38 | 1 | 64 | 103 | 0 | 48182 | 8001 | 12480368 |
| 1 | 0.005 | 0.42 | 1 | 64 | 89 | 0 | 42451 | 7607 | 11551321 |
| 2 | 0.007 | 0.45 | 0.98 | 63 | 78 | 1 | 38223 | 7278 | 10731293 |
| 3 | 0.009 | 0.46 | 0.88 | 56 | 65 | 8 | 33365 | 6834 | 9678076 |
| 4 | 0.011 | 0.48 | 0.78 | 50 | 55 | 14 | 27999 | 6274 | 8417562 |
| 5 | 0.013 | 0.51 | 0.69 | 44 | 43 | 20 | 22985 | 5653 | 7141678 |
| 6 | 0.015 | 0.51 | 0.62 | 40 | 38 | 24 | 18354 | 4991 | 5853576 |
| 7 | 0.017 | 0.48 | 0.45 | 29 | 32 | 35 | 14296 | 4296 | 4686913 |
| 8 | 0.02 | 0.5 | 0.33 | 21 | 21 | 43 | 9627 | 3336 | 3224916 |
| **9** | **0.025** | **0.59** | **0.2** | **13** | **9** | **51** | **4462** | **1982** | **1505419** |
|  | 0.027 | 0.5 | 0.11 | 7 | 7 | 57 | 3174 | 1556 | 1060175 |
|  | 0.029 | 0.55 | 0.09 | 6 | 5 | 58 | 2220 | 1176 | 740910 |
|  | 0.031 | 0.6 | 0.05 | 3 | 2 | 61 | 1534 | 878 | 497546 |
| **10** | 0.071 | N/A | 0.0 | 0 | 0 | 64 |  |  |  |

Table S23 Reference corpus sizes (not about PR in BD) for various cutoffs of the PR terms similarity scores; selected cutoff boldfaced; wordcounts according to tokenization via the python package spacy [30, version 3.0.6] – note that this differs from the corpus wordcount reported in the main text which is according to LancsBox/TreeTagger tokenisation

|  |  | Coded posts (n = 167) | | | | | All posts (n= 83,216) | | |
| --- | --- | --- | --- | --- | --- | --- | --- | --- | --- |
| Quantile | Cutoff PR < x | Precision | Recall | TP | FP | FN | posts | users | words |
| 1 | 0.005 | 1 | 0.14 | 14 | 0 | 89 | 5731 | 2629 | 929047 |
| 2 | 0.007 | 0.96 | 0.24 | 25 | 1 | 78 | 9959 | 3686 | 1749075 |
| 3 | 0.009 | 0.83 | 0.37 | 38 | 8 | 65 | 14817 | 4594 | 2802292 |
| 4 | 0.011 | 0.77 | 0.47 | 48 | 14 | 55 | 20183 | 5446 | 4062806 |
| **5** | **0.013** | **0.75** | **0.58** | **60** | **20** | **43** | **25197** | **6075** | **5338690** |
| 6 | 0.015 | 0.73 | 0.63 | 65 | 24 | 38 | 29828 | 6591 | 6626792 |
| 7 | 0.017 | 0.67 | 0.69 | 71 | 35 | 32 | 33886 | 6984 | 7793455 |
| 8 | 0.02 | 0.66 | 0.8 | 82 | 43 | 21 | 38555 | 7399 | 9255452 |
| 9 | 0.025 | 0.65 | 0.91 | 94 | 51 | 9 | 43720 | 7778 | 10974949 |
| 10 | 0.071 | 0.62 | 1 | 103 | 64 | 0 | 48182 | 8001 | 12480368 |

Document S4: Codebook to determine personal recovery relevance of Reddit posts

In this coding task, you will assess if Reddit posts are relevant for personal recovery (PR).

- 1. Coding task overview

Q1) Given a Reddit post, ask yourself, does the post indicate that their author (“user” in the following) can be regarded as being in PR, i.e., do they share experiences relevant to their PR in the post? Yes/No

Please consider Q1 carefully using the “PR guidelines” detailed below.

Q2)^[[18]](#footnote-18)^ Ask yourself: how confident am I about my answer to Q1? Give yourself a rating on a scale
from 1 to 5
1: not confident at all
2: slightly confident
3: somewhat confident
4: fairly confident
5: completely confident

For Q2, please go for your immediate reaction to the post – avoid over-thinking.

Table S24 shows the outline of the complete coding sheet.

To answer “yes” to “PR relevant” 1. BD and 2. Personal experience must be both “yes” and at least one of 3-5 must be “yes”.

To answer “no” to “PR relevant” either 1. BD or 2. Personal experience must be “no” or (if 1-2 are both “yes”) all of 3-5 must be “no”.

Table S24 Coding sheet outline

|  | Post | PR rele-vant | Confi-dence | 1. BD | 2. Personal experi-ence | 3. Living well with BD | 4. Personal understand-ding of BD | 5. Meaning-ful life |
| --- | --- | --- | --- | --- | --- | --- | --- | --- |
| Possible values | I feel … | yes/no | 1/2/3/ 4/5 | yes/no | yes/no | yes/no | yes/no | yes/no |

- 1. PR guidelines
     1. Definition of PR

Please use this definition of PR to guide your decision:
Recovery is a deeply personal, unique process of changing one’s attitudes, values, feelings, goals, skills and/or roles. It is a way of living a satisfying, hopeful and contributing life even with the limitations caused by the illness. Recovery involves the development of new meaning and purpose in one’s life as one grows beyond the catastrophic effects of mental illness” [25]

- - 1. Stages of PR

Please include only users from the “Contemplation” phase onwards (see Table S25).

Table S25 Stages of PR (Leamy et al., 2011, p. 449)


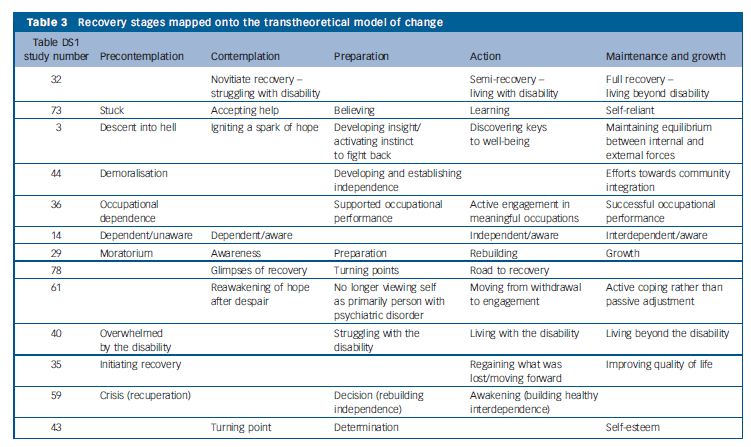


- - 1. Inclusion and exclusion criteria

Table S26 explains the inclusion and exclusion criteria, see Table S27 for example posts with explanations.

Yes: 1 + 2 + at least one of 3, 4, 5 are met
No: Everything else

Criteria 3-5 are based on the POETIC framework for PR in BD [31], see Figure S6 for an overview of the domains/categories.

Table S26 Inclusion and exclusion criteria for PR relevant

| **Rank** | **Criterium** | **Include (PR relevant = yes)** | **Exclude (PR relevant = no)** |
| --- | --- | --- | --- |
| 1 | BD | User shares experiences related to their BD diagnosis | User mainly focuses on experiences related to other issues / MH diagnoses |
| 2 | Personal experience | User shares their own personal experience | 1. User gives general advice to others, not speaking from their own experience 2. User makes a general comment 3. User asks question without providing own experience |
| 3 | (Hope for) living well with BD | User expresses that they (hope to) live well with BD (which might include experiencing symptoms) | Users express no hope in living well with BD, only talk about negative aspects and difficulties. According to [32] they could be seen as in the “Precontemplation” phase of the PR process, but here only include users who are at least in the “Contemplation” phase |
| 4 | Personal (non-medical) interpretation of BD | User expresses understanding their BD experiences in a personal framework, e.g. regarding mood as on a continuum and/or they express empowerment with respect to managing their moods and treatment choices | User expresses clinical understanding of their BD experiences, regard themselves as ‘ill’ and their experiences as fundamentally different from people without a BD diagnosis |
| 5 | Meaningful life vs. treating symptoms | User talks about (issues with) living a meaningful life, this can include, e.g., meaningful activities, live goals/roles, relationships, questioning/redefining their identity, negotiating tensions in living with BD | User only talks about / seeks advice in relation to treating symptoms. |

Table S27 Example posts for PR relevant inclusion and exclusion criteria

| Include | Exclude |
| --- | --- |
| 1. **Experiences in relation to BD** | **Experiences mainly not in relation to BD** |
| I honestly do NOT know how I would get through this without my husband, and he's the most supportive person I can imagine and then some. He even remembers things like the patterns of my moods so I can tell my doc, since he knows I can't remember crap. I know it's tough on him, but he's fantastic about trying to remember that when I'm irritable it *isn't* directed deliberately at him. He's also developed a great repertoire of tricks for cheering me up (when possible), easing my anxiety about things, and just trying to make things easier in general. Second to him, definitely my mother. But then, she's the one I inherited the bipolar from, so she more than understands. I feel incredibly lucky to have two such supportive people in my life. | I went down a very similar path with my ex, I have bipolar and PTSD from sexual assault, and after we graduated we were long distance. When we were together I always had no interest in it, I couldn't feel anything, and so I would drink to feel ok enough to do it. I wanted the intimacy, but couldn't deal with the physical part. I also felt like I 'had to do it' when he came to visit because it was so rare. Ultimately, he knows me well and saw pretty easily what I was doing, and we had a bunch of fights about it. He was extremely upset because he felt like I was 'drugging myself' to be with him, when he would have been fine and supportive without the sex, as much as he wanted it, because he was a respectable guy. We broke up but it's fine now, we talk occasional I can say from experience this did not work well for me. It stopped working and I drank/smoked more and more until it became a problem, I would drink a lot but some days because of my meds I would blackout after one drink. It scared me so I stopped drinking the day he broke up with me, I'm a year and a half sober now  Comment: Main focus is not BD but trauma-linked self-medication |
|  | I am a 2 fifth a night Addict diagnosed Bipolar.. and I don't Give a fuck I still get up next day and go to this place I call a job.. and I still function.. When that stops happening I'll quit. Comment: Main focus is on relationship to drinking (which indicates PR), but not BD |
| 1. **Share own personal experience** | **General advice/comment/question without own experience** |
| I was discharged by my therapist this week! BP2, diagnosed in Feb 2013. It was rough, you all know how it is. I knew I was getting depressed a lot but I had no idea how bad it was. A near suicide led me to talk to someone. Making that call was the hardest thing I have ever done. Today I feel great! No more racing negative thoughts. I still get depressed now and then but it's nothing like before. My therapist and I both believe I have the tools to make it through episodes on my own. I will still see my Pdoc regularly and I'll always be on meds. If I ever feel that it's getting too much to handle I can call the therapist anytime to schedule an appointment. It feels great knowing that I pulled through this!  Comment: indicates empowerment | This is probably going to sound weird, and I don't know anything about your situation so I'm speaking generally, but it's only worth being in a relationship if it's healthy for both people. If the non-BP person isn't getting what they need or the relationship is hurting them emotionally, then they have every right to leave. Relationships are completely voluntary, and if your SO isn't treating you well because of personality/attitude/whatever (bipolar aside), then that's not healthy. Why should BP guilt that person into staying? I'm sure it sounds terrible, but I guess it's important to remember that a non-bipolar partner has feelings and needs, too, and sometime that need involves being gone.  Comment: Giving general advice |
|  | I would be willing to do an interview as well. Bipolar I and currently in college. Comment: General comment |
|  | Intranasal ketamine is pretty well-established for treatment-resistant depression and seems to be useful in some cases for bipolar, but I'm looking for personal experiences with the treatment. Due to unrelated medical issues, I'm kind of running out of options for the bipolar, which is a real shame.  Comment: Asking a question without sharing own experiences |
|  | I apologize if my post seems mean spirited, but I have dealt with Bipolar stigma for eight years. The stigma against Bipolar Disorder is vicious and abhorrent. Your post simply furthers the stigma against people with Bipolar Disorder. I wish that you would rewrite your entire post in a manner that is less stigmatizing. Additionally, it would be good if you could explain exactly what you want from us. Do you just want to vent? Do you want feedback from people who have experienced similar circumstances? Do you want advice? The purpose of your post is completely unclear, and that is one of the reasons that it seems to be a troll post.  Comment: Asking a question without sharing details of own experiences |
| 1. **(Hope) for living well** | **Express no hope for living well with BD** |
| * working shifts before 8 am WILL make you cycle. Instead of telling people you have bipolar, tell them you have insomnia and the sleeping pills you take make you useless before then (75% true). * it's almost impossible to hear when I'm down but bipolar is about cycles. I will be down, I will be stable, I will be up. It will always change, which is good and bad. But I've gotten INFINITELY BETTER at recognizing where I am in the cycles and have treatment that works for me. * three years ago I was in the hospital and suicidal, now I'm moving to England to attend a top 10 university for a program I'm passionate about and have done things I never thought I would do, like hold down a job and travel on my own. **You never know what tomorrow will be like.**  Comment: Purpose and meaning: education/life goals, living well despite symptoms | One of the worst things about this... ...is that no matter what you do, what lifestyle change you make, or what medication you're on, you're still going to cycle. You will always be bipolar, you'll always be on the up and down. I recently started taking long walks and I feel great emotionally, but I still feel depression coming, and coming hard. Sorry if this seems morbid, just letting out how I feel.  Comment: Trying to live well but also lacking hope about this (Precontemplation phase) |
|  | Made so many enemies. Considering dropping out of school and moving away. Throughout my entire school life, I have made many enemies by just being stupid and manic. Dated and fucked all the wrong people. Then made enemies with them after bc I'm unstable. Then their friends hate me. And the new girls and past girls they dated hate me. Also I'm so confrontational when manic that I burned bridges with shitty friends but in the worst way possible. And today I got mistaken for my ex's new girlfriend (who hates me) at school by someone close to my ex. Not sure if they're fucking with me. Have been avoiding campus for 6 weeks and every time I've been there is an awkward interaction that literally sends me into a serious suicidal and irrational spiral. Almost killed myself a week ago after the last interaction. Feel stupid but kind of want to drop out. Maybe move away. I only have a year left but these 6 weeks have been UNBEARABLE. I think if I stay any longer I might just actually follow through and kill myself. Reaping the consequences of my actions suuuucks. Didn't realize my behavior is because I was bipolar until I got diagnosed recently. Now painfully self aware and ashamed. Has anyone experienced something like this before? It's so hard on me because I already want to kill myself on the daily anyway and now these stressors have exacerbated it by 100x.  Comment: This seems to be someone in a crisis about the consequences of mood driven behaviour, not PR because main options considered here seem to be suicide and leaving school, not living a meaningful life with BD |
| 1. **Personal understanding of their BD experiences** | **Understanding of their BD experiences only in frame of clinical diagnosis** |
| That sucks. I can't think of anyone in my life who doesn't know. Well, except Facebook, I'm not out on Facebook, but they're mainly people I don't really talk to, I just spy on them lol. I don't think most of the people I've told researched it like my bestie did, but they listen when I explain what it's like. I usually use the odometer analogy- most healthy people are between 60 &amp; 120. 60 is sad, it's like the day your mother died. Bipolar people can sometimes sit between 0 &amp; 60 - that's depression. Most people understand depression without a lengthy explanation. 120 is happy, like your wedding day or the birth of your first child. Bipolar people can go from 120 - 180. 180 you think you're god. In between 120 and 180 you can be hypersexual, hyperactive, talk a million miles an hour, start a thousand projects you're never going to finish, be a fitness fanatic, never sleep, come up with brilliant ideas, some actually brilliant, some clearly delusional, be charismatic, be the life of the party, indulge a little too much in the party and end up addicted to drugs and alcohol, and depending where you are on the spectrum, either kick ass at work by putting in 60 extra hours a week or get yourself fired for running into the office naked wielding a butter knife and screaming that the CIA are infiltrating the systems. There's a spectrum and not all bipolar people reach 180, so not everyone gets to the naked butter knife/ believing you're god stage. Personally, I have bipolar 1, so theoretically I can get there, but I haven't yet. People with bipolar 2 can only get to like 150 maybe.  Comment: User clearly seems to have a personal understanding that makes sense to them. They Contextualise their experience on a continuum – not binary (vs. what they have is some qualitative different illness to people without a BD diagnosis) | I'm very much in the same boat as you, even right down to being 30 right now and having had no idea what the heck was going on with me since adolescence and earlier even. I also think my bipolar is more in line with type II in terms of percentages of depression versus (hypo)mania, although I'm dxed type one simply because I have had two full blown manic episodes - although they were triggered by an antidepressant medication and quitting alcohol, and I don't think they would happen that way without a 'trigger'. At any rate, I agree, it's intense (yet sort of vindicating and a relief) to find out why I'm the way I am... not just a failure or someone not coping with the normal human condition, but rather, someone with a specific illness causing me to have extremes of moods and a baseline level of emotion that's mildly depressed at best.  Comment: User expresses clinical understanding of their BD symptoms, regards themselves as ill |
| 1. **Talk about meaningful life/activities** | **Talk about treating symptoms only** |
| Energy levels. Just a little background. I've been diagnosed as Bipolar 2 since I was 13 (now 20 (male)). I haven't had medication in a few years as I haven't had insurance and still don't. So more often than not I just have to deal with my moods and ride them out. I work as a valet at a pretty nice downtown hotel and am constantly running to get cars and talking to people who want to check in and valet with us. It's so draining. Today was one of those days where I just did not want to deal with any of the people at all but bit the bullet until I was off the clock. My girlfriend (20) wanted to go out tonight with a friend to go see a movie (she had planned this a week in advance) and I agreed at first. I got home today and just felt like shit. I really did not want to do anything. I just want to sit down all day and stare at the wall and listen to the hum of the air conditioner. I know it's a waste of time and counterproductive and could possibly further my depression during the day but I can't find the energy to care or to change it. How do you find the energy to get up and do things even when you don't want to? I know for work I do because I need the money. I feel bad for not going with my girlfriend but I'm really not up to it. And at least I'll be able to make it up to her another day where as with work, if I got fired I wouldn't. (I'm trying to make a point, but I'm very terrible at expressing it.) She told me she was mad at me for not wanting to go and she thinks I don't want to spend time with her. I do want to I just can't find it in me to go when I know I won't enjoy my night with them so why have me be a bother to them? She thinks me going out would make it better but I feel like I'd just want to be back at home, alone. So how do you guys do it? And how do your SO's, family, friends react to you when you are like this? I suppose this is kind of a rant but I just wanted some other opinions. Thank you guys.  Comment: User seems to struggle with relationships, connection, and work – PR in the sense of seeing the importance of these. | SSRI/SNRIs trigger rapid cycling for me. I was on Zoloft and Pristiq at different times. I would get hypomanic at first and then wind up all over the place. I had bad anxiety on Pristiq especially. My pdoc wants to keep me off all antidepressants due to my history of cycling, but that may not be the case for you. We are now exploring low dose Abilify with Lamictal. |
| I love this subreddit Hi everyone, I was formally diagnosed with BP2 at age 16. (27 now). I showed early signs and even ended up in anger management at age 6. Once diagnosed, I felt better Fast forward 10 years.. I'm on mood stabilizer (lamictal). During manic episodes, I cut myself off of it thinking I'm 'cured'. I end up treating people around me like shit including my husband who is so so supportive. Manic episodes just make me so big headed. I rage and tell him he doesn't deserve me, I'm too good for him, he will never find someone like me.. meanwhile he literally did nothing except say hello (Haha ._.) Now I'm back on lamictal and have been for about a year, but I randomly keep overthinking my diagnosis. I keep thinking I was misdiagnosed because I'm not manic, don't have paranoia ect. It feels weird even telling ppl I have bipolar bc I feel like they dont believe me because so many people use the term so freely to explain frequent mood changes Reading posts here really helps me see that I am correctly diagnosed, I'm thinking this way because the meds are working and I'm level-headed, and "normal" people have bipolar. So anyways, just wanna say thx. I appreciate everyone's post. It makes me feel like I'm not alone Comment: This post mixes clinical and PR perspectives – clinical recovery as they talk about medication and the importance of formal diagnosis, but also PR as they talk about the importance of their relationship and how it is strained by BD issues – therefore it fulfils (5) and is included – generally, it is ok if a post mixes perspectives as long as it contains some PR-relevant aspects according to criteria 3-5 |  |
|  | Does not meet any of 3-5 |
|  | Im bipolar 2. I had a psychologist who always downplayed my symptoms. If I said "mania", she would say, "Ah ah, HYPOmania." When I ended up in the hospital after leaving my boyfriend to become a porn star, she was fired. Dumb bitch. The only difference between 1 and 2 is that 1 has psychosis so.... Comment: Complaints about treatment but does not indicate living a meaningful life |
| Borderline case, low confidence – could be decided either PR relevant = yes or no | |
| My last hypomanic I opened up to a friend who is going through a messy break up... I told her she was beautiful, an amazing person that I used to fancy. That my wife used to be very jealous of her (so I made sure I never organised to see her without other people present). If I'd stopped there it would have been awesome. I then proceeded to send massive amounts of increasingly odd messages her way until I realised I was hypomanic a few days later. Then I apologised for my behaviour and resisted messaging her for a few days until I thought I had broken the back of the episode... I hadn't. I used the dying throws of the episode to further embarrass myself by trying to explain my bipolar to her in a very hypomanic way. I then brought a pot of pinkest pink paint for her (because if she was a colour she would be pinkest pink... it made sense to my borderline manic mind at the time). As far as hypomanic episodes go it was mild and I didn't do myself any long term physical or financial damage. The only damage is I think my friend is now a bit creeped out by me and I've damaged that relationship in a possibly irreparable way. Thankfully my friend lives a reasonable distance away and isn't interested in me or I could be writing about a whole bigger regret. Comment: This seems to be an account of a relationship being messed up in a hypomanic episode – in telling this one could infer that the user sees the value of relationships (fulfilling 5) – maybe lean towards “include” | |


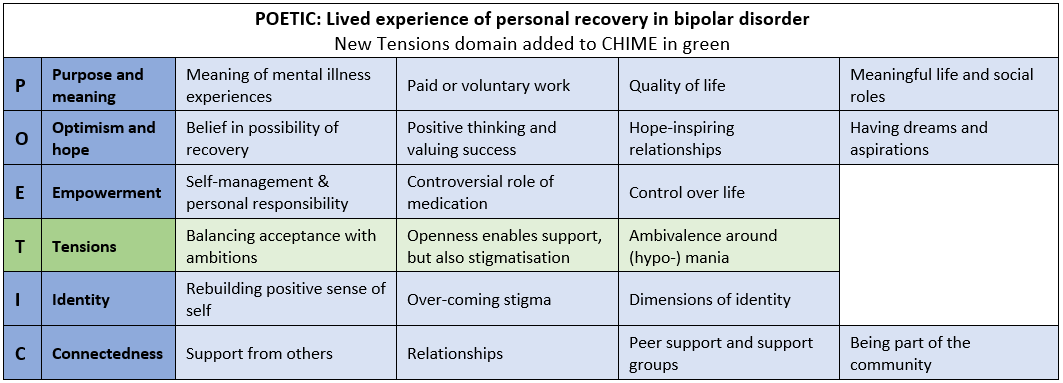


Figure S6 POETIC framework

Document S5: Quote selection and paraphrasing method

GJ selected the quotes and checked whether a Google search for the paraphrased quote (with and without the additional search term “reddit” added) would retrieve the original post in the top ten search results. If this was the case, the quote was shortened or paraphrased until the modified post no longer came up in the search results. Paraphrasing followed the method outline in Berry et al. [33]. CH checked the original quotes for suitability to illustrate the categories and compared the original quotes and the paraphrases to make sure the paraphrases were meaning and style preserving.

Document S6: Methodological considerations and details on generating the key lemmas

Baker [34] discusses some advantages and disadvantages of using lemmas instead of wordforms in keyword analyses. Analysing key lemmas could bring out aspects that might not show up in a key word analysis if individual wordforms of the same lemma (e.g., meditate, meditated, meditating) are too infrequent on their own. On the other hand, analysing key lemmas would mask diverging trends for individual wordforms of the same lemma. This would be the case, if, for example, meditate was significantly overused in one corpus, whereas meditating was overused in the other corpus because these differences would cancel out in the aggregated frequency for the lemma ‘meditate’. This study analysed key lemmas to emphasise analysing content and extend vocabulary coverage rather than focusing on more fine-grained linguistic differences.

Lemmas in #LancsBox 6.0 are pairs of a lemma and part of speech tag, e.g., need_v for need as a verb vs. need_n for need as a noun. Both lemmas and part of speech tags are automatically assigned via TreeTagger [35]. TreeTagger performs lemmatisation by a look-up in a manually created dictionary, therefore the lemmatisation accuracy should be nearly 100%^[[19]](#footnote-19)^. For wordforms that are not contained in the dictionary, the wordform is assigned as fall-back. TreeTagger achieved 86.6% part of speech tagging accuracy on a social media data test set consisting of Twitter messages and online chat dialogues [36], which might be comparable to the PR-BD corpus.

The 5% dispersion threshold for key lemmas was set in analogy to Hunt and Brookes [7] who required keywords to appear in 5% of their posts, however a per-post threshold was too restrictive for the PR-BD corpus.

There are no general recommendations in corpus linguistics for the relative size of the reference corpus to the target corpus. Brezina [37] argues that the larger the reference corpus the more reliable are the keywords, particularly since a larger reference corpus has a lower likelihood of containing zero instances of target corpus terms by chance. Rayson and colleagues [38] showed that at a log likelihood significance level of *P*<.0001 the chi-squared statistics for identifying keywords is reliable for expected word frequencies of 1/100000 or higher for a target corpus size of 1M words and a reference corpus size of 1M, 5M or 10M words.

Table S28 Key lemmas coded into POETIC categories (freq. = frequency, * = new key lemma, not a PR term)

| Domain/ category | Freq. | Key lemmas (percentage of key lemma coded into category) |
| --- | --- | --- |
| Purpose & meaning | 16469 |  |
| 4.1 Meaning of mental illness experiences | 1593 | childhood* 40%, sexual 37%, trauma 27%, insight 27%, grow* 23%, accept 20%, dad 17%, father_n 13%, environment* 13%, family 10%, deal_v 10%, positive 10%, dream_n 10%, guilt 10%, our* 10%, involve 7%, shame_n 7%, society 7%, challenge 7%, choose* 7%, raise_v* 7%, struggle_n* 7%, develop 3%, connect 3%, perspective 3%, open_adj 3%, belief 3%, kid 3%, child 3%, community 3%, motivation 3%, marry 3%, mother 3%, grateful 3%, relationship 3%, personal 3%, choice 3%, pursue 3%, baby* 3%, brother* 3%, deserve* 3%, provide* 3%, seek* 3% |
| 4.2 Spirituality | 1153 | god 70%, belief 37%, life 10%, teach* 10%, connect 7%, cure_v 7%, care_n 7%, quality* 7%, raise_v* 7%, develop 3%, trauma 3%, possibility 3%, close_adj 3%, activity 3%, insight 3%, positive 3%, mother 3%, capable 3%, study_v 3%, enjoy 3%, grateful 3%, personal 3%, guilt 3%, pursue 3%, baby* 3%, environment* 3%, future* 3%, yourself* 3% |
| 4.3 Quality of life | 2634 | hobby 70%, improve 40%, money 23%, parent_n 17%, marry 17%, enjoy 13%, social 10%, exercise_v 10%, activity 10%, husband 10%, manage 10%, job 10%, wife 10%, degree 10%, adult* 10%, quality* 10%, decision 7%, involve 7%, success 7%, girlfriend 7%, engage 7%, learn 7%, live 7%, close_adj 7%, kid 7%, life 7%, personal 7%, skill 7%, resource 7%, routine 7%, recover 7%, education 7%, responsible 7%, guilty 7%, active 7%, grow* 7%, maintain* 7%, develop 3%, family 3%, connect 3%, exercise_n 3%, offer_v 3%, progress 3%, partner_n 3%, community 3%, professional 3%, boyfriend 3%, stability 3%, mother 3%, capable 3%, cure_v 3%, relationship 3%, dad 3%, goal 3%, mom 3%, healthy 3%, dream_n 3%, lifestyle 3%, sexual 3%, pursue 3%, bear_v* 3%, brother*3%, career* 3%, educate* 3%, environment* 3%, forward_adv* 3%, marriage* 3%, our* 3%, provide* 3%, raise_v* 3%, teach* 3% |
| 4.4 Meaningful life and social goals | 687 | goal 27%, dream_n 10%, progress 7%, community 7%, money 7%, pursue 7%, step_n* 7%, yourself* 7%, decision 3%, involve 3%, success 3%, manage 3%, capable 3%, life 3%, hobby 3%, care_n 3%, degree 3%, lifestyle 3%, graduate 3%, education 3%, career* 3%, grow* 3% |
| 4.5 Meaningful life and social roles | 5769 | kid 57%, parent_n 53%, daughter 53%, baby* 53%, child 50%, son 47%, bear_v* 43%, raise_v* 37%, chance 30%, environment* 30%, decision 27%, mom 27%, grow* 23%, risk_n 20%, society 20%, father_n 20%, challenge 17%, develop 13%, perspective 13%, possibility 13%, capable 13%, life 13%, choice 13%, responsible 13%, childhood* 13%, our* 13%, provide* 13%, struggle_n* 13%, family 10%, care_n 10%, dad 10%, choose* 10%, future* 10%, opportunity 7%, deal_v 7%, open_adj 7%, cope 7%, partner_n 7%, husband 7%, stability 7%, marry 7%, mother 7%, support_v 7%, helpful 7%, healthy 7%, guilty 7%, adult* 7%, deserve* 7%, forgive* 7%, position* 7%, successful* 7%, yourself* 7%, involve 3%, connect 3%, girlfriend 3%, offer_v 3%, share_v 3%, boyfriend 3%, motivation 3%, study_v 3%, grateful 3%, relationship 3%, god 3%, wife 3%, support_n 3%, dream_n 3%, resource 3%, guilt 3%, sexual 3%, recover 3%, effort* 3%, marriage* 3%, quality* 3%, seek* 3% |
| 4.6 Rebuilding of life | 218 | deal_v 3%, capable 3%, life 3%, skill 3%, recover 3% |
| 4.7 Paid or voluntary work or work related activities or formal education | 4415 | graduate 77%, college 77%, job 70%, degree 63%, career* 60%, study_v 47%, education 47%, position* 43%, capable 27%, successful* 27%, teach* 27%, opportunity 23%, professional 20%, pursue 20%, community 17%, enjoy 17%, offer_v 13%, skill 13%, responsibility 13%, struggle_n* 13%, chance 10%, learn 10%, possibility 10%, progress 10%, support_v 10%, wife 10%, choice 10%, dream_n 10%, recover 10%, effort* 10%, quality* 10%, schedule* 10%, decision 7%, accept 7%, success 7%, engage 7%, improve 7%, risk_n 7%, motivation 7%, manage 7%, guilty 7%, adult* 7%, deserve* 7%, environment* 7%, provide* 7%, raise_v* 7%, open_adj 3%, stability 3%, recovery 3%, helpful 3%, grateful 3%, hope_n 3%, life 3%, relationship 3%, challenge 3%, dad 3%, money 3%, goal 3%, member 3%, routine 3%, sexual 3%, responsible 3%, active 3%, baby* 3%, brother* 3%, childhood* 3%, choose* 3%, forward_adv* 3%, future* 3%, maintain* 3%, yourself* 3% |
| Optimism & hope | 3525 |  |
| 2.1 Belief in possibility of recovery | 1346 | cure_v 27%, hope_n 23%, manage 20%, progress 17%, recover 17%, live 13%, recovery 13%, life 10%, deserve* 10%, risk_n 7%, career* 7%, forward_adv* 7%, accept 3%, success 3%, deal_v 3%, exercise_v 3%, strength 3%, community 3%, stability 3%, confidence 3%, society 3%, challenge 3%, adult* 3%, educate* 3%, future* 3%, quality* 3%, raise_v* 3%, struggle_n* 3% |
| 2.2 Hope-inspiring relationships | 671 | successful* 27%, strength 13%, dad 13%, father_n 13%, encourage* 13%, success 10%, share_v 10%, hope_n 10%, recovery 7%, mother 7%, brother* 7%, social 3%, involve 3%, chance 3%, connect 3%, shame_n 3%, perspective 3%, possibility 3%, live 3%, parent_n 3%, boyfriend 3%, cure_v 3%, grateful 3%, challenge 3%, mom 3%, daughter 3%, resource 3%, guilty 3%, childhood* 3%, raise_v* 3% |
| 2.3 Positive thinking and valuing success | 1202 | opportunity 37%, grateful 37%, strength 27%, progress 27%, forward_adv* 23%, success 17%, positive 17%, enjoy 17%, hope_n 13%, guilt 13%, learn 10%, challenge 10%, choose* 10%, effort* 10%, future* 10%, teach* 10%, connect 7%, encourage* 7%, our* 7%, successful* 7%, social 3%, accept 3%, shame_n 3%, perspective 3%, friendship 3%, trauma 3%, open_adj 3%, cope 3%, partner_n 3%, community 3%, capable 3%, helpful 3%, society 3%, god 3%, active 3%, pursue 3%, forgive* 3%, grow* 3%, maintain* 3%, quality* 3%, raise_v* 3%, seek* 3%, yourself* 3% |
| 2.4 Having dreams and aspirations | 306 | dream_n 27%, future* 23%, hope_n 7%, pursue 7%, forward_adv* 7%, successful* 7%, deal_v 3%, possibility 3%, open_adj 3%, stability 3%, active 3% |
| Empowerment | 8994 |  |
| 5.1 Self-management and personal responsibility | 6396 | diet_n 83%, lifestyle 80%, routine 80%, schedule* 73%, exercise_n 70%, exercise_v 67%, cope 60%, skill 53%, responsibility 53%, maintain* 47%, healthy 43%, goal 37%, learn 33%, develop 30%, effort* 30%, step_n* 30%, yourself* 30%, activity 27%, active 27%, positive 23%, choose* 23%, improve 20%, recovery 17%, care_n 17%, deal_v 17%, manage 17%, hobby 17%, environment* 17%, teach* 17%, success 13%, stability 13%, cure_v 13%, challenge 13%, social 10%, belief 10%, life 10%, choice 10%, guilt 10%, responsible 10%, pursue 10%, adult* 10%, forward_adv* 10%, decision 7%, accept 7%, connect 7%, perspective7%, trauma 7%, progress 7%, live 7%, child 7%, capable 7%, study_v 7%, helpful 7%, enjoy 7%, personal 7%, money 7%, resource 7%, sexual 7%, bear_v* 7%, our* 7%, provide* 7%, quality* 7%, struggle_n* 7%, successful* 7%, opportunity 3%, chance 3%, engage 3%, strength 3%, shame_n 3%, possibility 3%, open_adj 3%, close_adj 3%, husband 3%, boyfriend 3%, motivation 3%, dad 3%, wife 3%, education 3%, guilty 3%, career* 3%, encourage* 3%, forgive* 3%, group* 3%, position* 3%, seek* 3% |
| 5.2 Control over life | 1309 | educate* 47%, resource 33%, choice 30%, helpful 20%, education 20%, learn 13%, manage 13%, recovery 10%, effort* 10%, teach* 10%, develop 7%, decision 7%, provide* 7%, seek* 7%, involve 3%, connect 3%, deal_v 3%, strength 3%, perspective 3%, share_v 3%, progress 3%, improve 3%, close_adj 3%, activity 3%, risk_n 3%, community 3%, professional 3%, marry 3%, capable 3%, study_v 3%, job 3%, challenge 3%, care_n 3%, personal 3%, money 3%, active 3%, pursue 3%, adult* 3%, choose* 3%, encourage* 3%, forgive* 3%, forward_adv* 3%, step_n* 3%, yourself* 3% |
| 5.3 Controversial role of medication | 1289 | risk_n 23%, possibility 20%, quality* 20%, cure_v 17%, professional 13%, recovery 13%, baby* 13%, exercise_n 10%, close_adj 10%, dream_n 10%, develop 7%, accept 7%, success 7%, chance 7%, exercise_v 7%, offer_v 7%, cope 7%, improve 7%, stability 7%, lifestyle 7%, routine 7%, raise_v* 7%, involve 3%, connect 3%, diet_n 3%, open_adj 3%, positive 3%, community 3%, motivation 3%, confidence 3%, mother 3%, capable 3%, study_v 3%, job 3%, helpful 3%, grateful 3%, hope_n 3%, life 3%, relationship 3%, challenge 3%, care_n 3%, personal 3%, goal 3%, choice 3%, degree 3%, responsibility 3%, responsible 3%, pursue 3%, brother* 3%, choose* 3%, forward_adv* 3%, grow* 3%, maintain* 3%, our* 3%, position* 3%, provide* 3%, seek* 3%, step_n* 3% |
| Tensions | 1796 |  |
| 6.1 Balancing acceptance with ambitions | 395 | opportunity 7%, care_n 7%, schedule* 7%, develop 3%, accept 3%, success 3%, strength 3%, boyfriend 3%, stability 3%, study_v 3%, job 3%, hope_n 3%, challenge 3%, goal 3%, responsibility 3%, resource 3%, pursue 3%, our* 3%, struggle_n* 3%, yourself* 3% |
| 6.2 Openness enables support and stigmatisation | 1137 | open_adj 30%, close_adj 20%, stigma 17%, adult* 17%, share_v 13%, supportive 13%, accept 7%, friendship 7%, belief 7%, personal 7%, member 7%, choose* 7%, encourage* 7%, struggle_n* 7%, develop 3%, social 3%, opportunity 3%, success 3%, chance 3%, offer_v 3%, possibility 3%, live 3%, positive 3%, motivation 3%, manage 3%, job 3%, helpful 3%, grateful 3%, life 3%, society 3%, challenge 3%, care_n 3%, dad 3%, choice 3%, sexual 3%, baby* 3%, career* 3%, deserve* 3%, educate* 3%, future* 3%, group* 3%, position* 3%, provide* 3%, raise_v* 3% |
| 6.3 Ambivalence around (hypo-)mania | 264 | confidence 10%, progress 7%, motivation 7%, enjoy 7%, perspective 3%, possibility 3%, live 3%, husband 3%, manage 3%, god 3%, goal 3%, choice 3%, dream_n 3%, skill 3%, sexual 3%, encourage* 3% |
| Identity | 3405 |  |
| 3.1 Dimensions of identity | 528 | sexual 20%, belief 10%, accept 7%, strength 7%, life 7%, social 3%, chance 3%, girlfriend 3%, share_v 3%, supportive 3%, cure_v 3%, society 3%, adult* 3%, future* 3% |
| 3.2 Rebuilding or redefining positive sense of self | 1640 | confidence 40%, forgive* 37%, guilt 27%, deserve* 23%, shame_n 20%, yourself* 17%, strength 13%, learn 13%, grow* 13%, guilty 10%, successful* 10%, decision 7%, accept 7%, success 7%, capable 7%, responsibility 7%, childhood* 7%, effort* 7%, forward_adv* 7%, future* 7%, step_n* 7%, social 3%, family 3%, deal_v 3%, exercise_v 3%, perspective 3%, share_v 3%, improve 3%, live 3%, insight 3%, motivation 3%, manage 3%, marry 3%, support_v 3%, cure_v 3%, study_v 3%, job 3%, enjoy 3%, hope_n 3%, society 3%, care_n 3%, personal 3%, goal 3%, support_n 3%, healthy 3%, dream_n 3%, lifestyle 3%, sexual 3%, responsible 3%, active 3%, pursue 3%, adult* 3%, baby* 3%, bear_v* 3%, encourage* 3%, our* 3%, quality* 3% |
| 3.3 Over-coming stigma | 1237 | stigma 80%, shame_n 23%, society 23%, personal 13%, responsible 10%, bear_v* 10%, social 7%, open_adj 7%, share_v 7%, belief 7%, close_adj 7%, child 7%, community 7%, helpful 7%, money 7%, daughter 7%, education 7%, choose* 7%, educate* 7%, environment* 7%, group* 7%, decision 3%, involve 3%, accept 3%, family 3%, diet_n 3%, partner_n 3%, supportive 3%, positive 3%, risk_n 3%, professional 3%, mother 3%, capable 3%, grateful 3%, member 3%, responsibility 3%, graduate 3%, guilty 3%, active 3%, adult* 3%, baby* 3%, brother* 3%, career* 3%, deserve* 3%, forward_adv* 3%, future* 3%, raise_v* 3%, step_n* 3%, struggle_n* 3%, yourself* 3% |
| Connectedness | 15709 |  |
| 1.1 Peer support and support groups | 2711 | group* 67%, support_n 47%, share_v 40%, insight 33%, personal 30%, community 27%, member 23%, hope_n 23%, perspective 20%, helpful 20%, offer_v 17%, connect 13%, support_v 13%, resource 13%, friendship 7%, learn 7%, positive 7%, recovery 7%, cure_v 7%, grateful 7%, responsible 7%, encourage* 7%, position* 7%, provide* 7%, step_n* 7%, social 3%, decision 3%, opportunity 3%, involve 3%, family 3%, chance 3%, girlfriend 3%, strength 3%, trauma 3%, live 3%, supportive 3%, kid 3%, motivation 3%, enjoy 3%, life 3%, degree 3%, skill 3%, education 3%, effort* 3%, struggle_n* 3%, teach* 3% |
| 1.2 Relationships | 6884 | relationship 73%, friendship 53%, partner_n 50%, boyfriend 50%, engage 40%, forgive* 40%, marriage* 40%, girlfriend 37%, marry 33%, wife 30%, deserve* 30%, our* 30%, live 27%, father_n 27%, maintain* 27%, social 23%, connect 23%, open_adj 23%, mom 23%, family 20%, close_adj 20%, mother 20%, guilty 20%, sexual 17%, involve 17%, chance 17%, member 17%, choose* 17%, deal_v 13%, perspective 13%, improve 13%, supportive 13%, kid 13%, husband 13%, dad 13%, daughter 13%, responsibility 13%, struggle_n* 13%, accept 10%, offer_v 10%, progress 10%, challenge 10%, healthy 10%, son 10%, guilt 10%, responsible 10%, brother* 10%, effort* 10%, position* 10%, step_n* 10%, develop 7%, success 7%, trauma 7%, share_v 7%, activity 7%, capable 7%, support_v 7%, enjoy 7%, grateful 7%, life 7%, skill 7%, pursue 7%, childhood* 7%, forward_adv* 7%, quality* 7%, decision 3%, opportunity 3%, strength 3%, possibility 3%, parent_n 3%, child 3%, positive 3%, risk_n 3%, community 3%, stability 3%, motivation 3%, recovery 3%, confidence 3%, study_v 3%, college 3%, personal 3%, choice 3%, dream_n 3%, routine 3%, education 3%, active 3%, adult* 3%, baby* 3%, encourage* 3%, group* 3%, grow* 3%, raise_v* 3%, schedule* 3%, seek* 3%, yourself* 3% |
| 1.3 Support from others | 5955 | supportive 63%, seek* 60%, support_v 57%, husband 50%, professional 47%, support_n 47%, active 40%, provide* 37%, wife 33%, resource 30%, offer_v 30%, educate* 30%, family 27%, mother 27%, care_n 27%, member 27%, partner_n 23%, helpful 23%, mom 23%, son 23%, encourage* 23%, girlfriend 20%, trauma 20%, grateful 20%, step_n* 20%, teach* 20%, boyfriend 17%, engage 17%, shame_n 17%, insight 17%, responsible 17%, accept 13%, perspective 13%, belief 13%, marry 13%, study_v 13%, social 10%, connect 10%, possibility 10%, live 10%, child 10%, positive 10%, community 10%, manage 10%, dad 10%, daughter 10%, pursue 10%, adult* 10%, brother* 10%, effort* 10%, marriage* 10%, position* 10%, develop 7%, decision 7%, involve 7%, exercise_n 7%, cope 7%, share_v 7%, motivation 7%, capable 7%, skill 7%, recover 7%, environment* 7%, group* 7%, quality* 7%, struggle_n* 7%, opportunity 3%, success 3%, deal_v 3%, strength 3%, learn 3%, open_adj 3%, progress 3%, close_adj 3%, parent_n 3%, recovery 3%, life 3%, challenge 3%, god 3%, money 3%, father_n 3%, choice 3%, lifestyle 3%, education 3%, guilty 3%, bear_v* 3%, choose* 3%, deserve* 3%, grow* 3%, schedule* 3% |
| 1.4 Being part of the community | 159 | connect 7%, community 7%, society 7%, involve 3%, activity 3%, positive 3%, husband 3%, helpful 3%, money 3%, member 3%, active 3%, effort* 3%, maintain* 3% |
| Not POETIC | 9302 |  |
| X.1 Other MH issues | 5840 | motivation 50%, stability 43%, recover 43%, activity 37%, confidence 37%, money 37%, guilty 37%, involve 33%, brother* 33%, friendship 30%, trauma 30%, risk_n 30%, bear_v* 30%, marriage* 30%, engage 27%, deal_v 23%, shame_n 20%, society 20%, father_n 20%, guilt 20%, forward_adv* 20%, future* 20%, quality* 20%, struggle_n* 20%, possibility 17%, social 17%, cope 17%, child 17%, insight 17%, enjoy 17%, god 17%, goal 17%, healthy 17%, decision 13%, family 13%, chance 13%, live 13%, belief 13%, kid 13%, marry 13%, challenge 13%, personal 13%, degree 13%, dream_n 13%, encourage* 13%, our* 13%, success 10%, exercise_v 10%, strength 10%, close_adj 10%, recovery 10%, manage 10%, life 10%, hobby 10%, mom 10%, choice 10%, son 10%, member 10%, responsible 10%, pursue 10%, career* 10%, group* 10%, yourself* 10%, connect 7%, girlfriend 7%, diet_n 7%, perspective 7%, improve 7%, parent_n 7%, positive 7%, boyfriend 7%, mother 7%, cure_v 7%, study_v 7%, hope_n 7%, daughter 7%, childhood* 7%, choose* 7%, educate* 7%, forgive* 7%, grow* 7%, provide* 7%, seek* 7%, step_n* 7%, develop 3%, opportunity 3%, exercise_n 3%, offer_v 3%, open_adj 3%, progress 3%, partner_n 3%, husband 3%, professional 3%, capable 3%, support_v 3%, relationship 3%, dad 3%, responsibility 3%, education 3%, adult* 3%, baby* 3%, deserve* 3%, environment* 3%, position* 3%, successful* 3% |
| X.2 Storytelling | 2187 | girlfriend 20%, college 20%, brother* 20%, dad 17%, graduate 17%, childhood* 17%, close_adj 13%, mother 13%, adult* 13%, develop 10%, deal_v 10%, offer_v 10%, parent_n 10%, recover 10%, encourage* 10%, decision 7%, family 7%, possibility 7%, open_adj 7%, activity 7%, husband 7%, boyfriend 7%, recovery 7%, marry 7%, cure_v 7%, money 7%, wife 7%, healthy 7%, choice 7%, son 7%, member 7%, daughter 7%, responsible 7%, career* 7%, grow* 7%, position* 7%, raise_v* 7%, successful* 7%, involve 3%, accept 3%, chance 3%, diet_n 3%, learn 3%, share_v 3%, live 3%, kid 3%, child 3%, positive 3%, risk_n 3%, community 3%, motivation 3%, confidence 3%, study_v 3%, job 3%, enjoy 3%, life 3%, stigma 3%, mom 3%, dream_n 3%, guilt 3%, baby* 3%, deserve* 3%, effort* 3%, forward_adv* 3%, future* 3%, marriage* 3%, provide* 3%, seek* 3%, step_n* 3% |
| X.3 Forum interaction | 1074 | care_n 13%, stability 10%, healthy 10%, marriage* 10%, opportunity 7%, exercise_n 7%, strength 7%, professional 7%, recovery 7%, relationship 7%, environment* 7%, maintain* 7%, raise_v* 7%, yourself* 7%, chance 3%, shame_n 3%, perspective 3%, progress 3%, partner_n 3%, live 3%, parent_n 3%, activity 3%, positive 3%, boyfriend 3%, mother 3%, capable 3%, cure_v 3%, enjoy 3%, grateful 3%, challenge 3%, personal 3%, dad 3%, wife 3%, mom 3%, father_n 3%, degree 3%, son 3%, skill 3%, pursue 3%, baby* 3%, brother* 3%, childhood* 3%, deserve* 3%, encourage* 3%, future* 3%, grow* 3%, our* 3%, position* 3%, schedule* 3%, seek* 3% |
| X.4 General discussion | 201 | society 7%, social 3%, perspective 3%, partner_n 3%, hope_n 3%, care_n 3%, mom 3%, adult* 3%, environment* 3%, future* 3%, our* 3% |

Table S29 Categories assigned to key lemmas (based on 30 coded concordance lines each) (freq. = frequency, * = new key lemma, not a PR term)

|  | Key lemma | Freq. | Coded categories with percentage |
| --- | --- | --- | --- |
| 1 | life | 4732 | 4.5 13%, 2.1 10%, 4.2 10%, 5.1 10%, X.1 10%, 1.2 6%, 3.1 6%, 4.3 6%, 1.1 3%, 1.3 3%, 4.4 3%, 4.6 3%, 4.7 3%, 5.3 3%, 6.2 3%, X.2 3% |
| 2 | family | 1988 | 1.3 26%, 1.2 20%, X.1 13%, 4.1 10%, 4.5 10%, X.2 6%, 1.1 3%, 3.2 3%, 3.3 3%, 4.3 3% |
| 3 | job | 1831 | 4.7 70%, 4.3 10%, 3.2 3%, 5.2 3%, 5.3 3%, 6.1 3%, 6.2 3%, X.2 3% |
| 4 | relationship | 1777 | 1.2 73%, X.3 6%, 4.1 3%, 4.3 3%, 4.5 3%, 4.7 3%, 5.3 3%, X.1 3% |
| 5 | live | 1614 | 1.2 26%, 2.1 13%, X.1 13%, 1.3 10%, 4.3 6%, 5.1 6%, 1.1 3%, 2.2 3%, 3.2 3%, 6.2 3%, 6.3 3%, X.3 3%, X.2 3% |
| 6 | yourself* | 1450 | 5.1 30%, 3.2 16%, X.1 10%, 4.4 6%, 4.5 6%, X.3 6%, 1.2 3%, 2.3 3%, 3.3 3%, 4.2 3%, 4.7 3%, 5.2 3%, 6.1 3% |
| 7 | our* | 1416 | 1.2 30%, 4.5 13%, X.1 13%, 4.1 10%, 2.3 6%, 5.1 6%, 3.2 3%, 4.3 3%, 5.3 3%, 6.1 3%, X.3 3%, X.4 3% |
| 8 | learn | 1400 | 5.1 33%, 3.2 13%, 5.2 13%, 2.3 10%, 4.7 10%, 1.1 6%, 4.3 6%, 1.3 3%, X.2 3% |
| 9 | parent_n | 1275 | 4.5 53%, 4.3 16%, X.2 10%, X.1 6%, 1.2 3%, 1.3 3%, 2.2 3%, X.3 3% |
| 10 | child | 1243 | 4.5 50%, X.1 16%, 1.3 10%, 3.3 6%, 5.1 6%, 1.2 3%, 4.1 3%, X.2 3% |
| 11 | support_n | 1238 | 1.1 46%, 1.3 46%, 3.2 3%, 4.5 3% |
| 12 | Kid | 1146 | 4.5 56%, 1.2 13%, X.1 13%, 4.3 6%, 1.1 3%, 4.1 3%, X.2 3% |
| 13 | deal_v | 1141 | X.1 23%, 5.1 16%, 1.2 13%, 4.1 10%, X.2 10%, 4.5 6%, 1.3 3%, 2.1 3%, 2.4 3%, 3.2 3%, 4.6 3%, 5.2 3% |
| 14 | manage | 934 | 2.1 20%, 5.1 16%, 5.2 13%, 1.3 10%, 4.3 10%, X.1 10%, 4.7 6%, 3.2 3%, 4.4 3%, 6.2 3%, 6.3 3% |
| 15 | mom | 845 | 4.5 26%, 1.2 23%, 1.3 23%, X.1 10%, 2.2 3%, 4.3 3%, X.2 3%, X.3 3%, X.4 3% |
| 16 | care_n | 827 | 1.3 26%, 5.1 16%, X.3 13%, 4.5 10%, 4.2 6%, 6.1 6%, 3.2 3%, 4.4 3%, 5.2 3%, 5.3 3%, 6.2 3%, X.4 3% |
| 17 | healthy | 811 | 5.1 43%, X.1 16%, 1.2 10%, X.3 10%, X.2 6%, 4.5 6%, 3.2 3%, 4.3 3% |
| 18 | accept | 733 | 4.1 20%, 1.3 13%, 1.2 10%, 3.1 6%, 3.2 6%, 4.7 6%, 5.1 6%, 5.3 6%, 6.2 6%, 2.1 3%, 2.3 3%, 3.3 3%, 6.1 3%, X.2 3% |
| 19 | group* | 717 | 1.1 66%, X.1 10%, 1.3 6%, 3.3 6%, 1.2 3%, 5.1 3%, 6.2 3% |
| 20 | college | 715 | 4.7 76%, X.2 20%, 1.2 3% |
| 21 | cope | 665 | 5.1 60%, X.1 16%, 1.3 6%, 4.5 6%, 5.3 6%, 2.3 3% |
| 22 | husband | 649 | 1.3 50%, 1.2 13%, 4.3 10%, 4.5 6%, X.2 6%, 1.4 3%, 5.1 3%, 6.3 3%, X.1 3% |
| 23 | mother | 609 | 1.3 26%, 1.2 20%, X.2 13%, 2.2 6%, 4.5 6%, X.1 6%, 3.3 3%, 4.1 3%, 4.2 3%, 4.3 3%, 5.3 3%, X.3 3% |
| 24 | social | 599 | 1.2 23%, X.1 16%, 1.3 10%, 4.3 10%, 5.1 10%, 3.3 6%, 1.1 3%, 2.2 3%, 2.3 3%, 3.1 3%, 3.2 3%, 6.2 3%, X.4 3% |
| 25 | Dad | 574 | 4.1 16%, X.2 16%, 1.2 13%, 2.2 13%, 1.3 10%, 4.5 10%, 4.3 3%, 4.7 3%, 5.1 3%, 6.2 3%, X.1 3%, X.3 3% |
| 26 | partner_n | 572 | 1.2 50%, 1.3 23%, 4.5 6%, 2.3 3%, 3.3 3%, 4.3 3%, X.1 3%, X.3 3%, X.4 3% |
| 27 | decision | 566 | 4.5 26%, X.1 13%, 1.3 6%, 3.2 6%, 4.3 6%, 4.7 6%, 5.1 6%, 5.2 6%, X.2 6%, 1.1 3%, 1.2 3%, 3.3 3%, 4.4 3% |
| 28 | money | 565 | X.1 36%, 4.3 23%, 3.3 6%, 4.4 6%, 5.1 6%, X.2 6%, 1.3 3%, 1.4 3%, 4.7 3%, 5.2 3% |
| 29 | share_v | 499 | 1.1 40%, 6.2 13%, 2.2 10%, 1.2 6%, 1.3 6%, 3.3 6%, 3.1 3%, 3.2 3%, 4.5 3%, 5.2 3%, X.2 3% |
| 30 | Goal | 485 | 5.1 36%, 4.4 26%, X.1 16%, 3.2 3%, 4.3 3%, 4.7 3%, 5.3 3%, 6.1 3%, 6.3 3% |
| 31 | boyfriend | 469 | 1.2 50%, 1.3 16%, X.1 6%, X.2 6%, 2.2 3%, 4.3 3%, 4.5 3%, 5.1 3%, 6.1 3%, X.3 3% |
| 32 | support_v | 464 | 1.3 56%, 1.1 13%, 4.7 10%, 1.2 6%, 4.5 6%, 3.2 3%, X.1 3% |
| 33 | Wife | 464 | 1.3 33%, 1.2 30%, 4.3 10%, 4.7 10%, X.2 6%, 4.5 3%, 5.1 3%, X.3 3% |
| 34 | personal | 441 | 1.1 30%, 3.3 13%, X.1 13%, 4.3 6%, 5.1 6%, 6.2 6%, 1.2 3%, 3.2 3%, 4.1 3%, 4.2 3%, 5.2 3%, 5.3 3%, X.3 3% |
| 35 | father_n | 435 | 1.2 26%, 4.5 20%, X.1 20%, 2.2 13%, 4.1 13%, 1.3 3%, X.3 3% |
| 36 | helpful | 434 | 1.3 23%, 1.1 20%, 5.2 20%, 3.3 6%, 4.5 6%, 5.1 6%, 1.4 3%, 2.3 3%, 4.7 3%, 5.3 3%, 6.2 3% |
| 37 | choice | 433 | 5.2 30%, 4.5 13%, 4.7 10%, 5.1 10%, X.1 10%, X.2 6%, 1.2 3%, 1.3 3%, 4.1 3%, 5.3 3%, 6.2 3%, 6.3 3% |
| 38 | supportive | 421 | 1.3 63%, 1.2 13%, 6.2 13%, 1.1 3%, 3.1 3%, 3.3 3% |
| 39 | god | 416 | 4.2 70%, X.1 16%, 1.3 3%, 2.3 3%, 4.5 3%, 6.3 3% |
| 40 | positive | 414 | 5.1 23%, 2.3 16%, 1.3 10%, 4.1 10%, 1.1 6%, X.1 6%, 1.2 3%, 1.4 3%, 3.3 3%, 4.2 3%, 5.3 3%, 6.2 3%, X.2 3%, X.3 3% |
| 41 | close_adj | 412 | 1.2 20%, 6.2 20%, X.2 13%, 5.3 10%, X.1 10%, 3.3 6%, 4.3 6%, 1.3 3%, 4.2 3%, 5.1 3%, 5.2 3% |
| 42 | degree | 401 | 4.7 63%, X.1 13%, 4.3 10%, 1.1 3%, 4.4 3%, 5.3 3%, X.3 3% |
| 43 | hope_n | 390 | 1.1 23%, 2.1 23%, 2.3 13%, 2.2 10%, 2.4 6%, X.1 6%, 3.2 3%, 4.7 3%, 5.3 3%, 6.1 3%, X.4 3% |
| 44 | seek* | 389 | 1.3 60%, 5.2 6%, X.1 6%, 1.2 3%, 2.3 3%, 4.1 3%, 4.5 3%, 5.1 3%, 5.3 3%, X.2 3%, X.3 3% |
| 45 | son | 387 | 4.5 46%, 1.3 23%, 1.2 10%, X.1 10%, X.2 6%, X.3 3% |
| 46 | stability | 386 | X.1 43%, 5.1 13%, X.3 10%, 4.5 6%, 5.3 6%, 1.2 3%, 2.1 3%, 2.4 3%, 4.3 3%, 4.7 3%, 6.1 3% |
| 47 | dream_n | 376 | 2.4 26%, X.1 13%, 4.1 10%, 4.4 10%, 4.7 10%, 5.3 10%, 1.2 3%, 3.2 3%, 4.3 3%, 4.5 3%, 6.3 3%, X.2 3% |
| 48 | chance | 369 | 4.5 30%, 1.2 16%, X.1 13%, 4.7 10%, 5.3 6%, 1.1 3%, 2.2 3%, 3.1 3%, 5.1 3%, 6.2 3%, X.2 3%, X.3 3% |
| 49 | develop | 363 | 5.1 30%, 4.5 13%, X.2 10%, 1.2 6%, 1.3 6%, 5.2 6%, 5.3 6%, 4.1 3%, 4.2 3%, 4.3 3%, 6.1 3%, 6.2 3%, X.1 3% |
| 50 | grow* | 358 | 4.1 23%, 4.5 23%, 3.2 13%, 4.3 6%, X.1 6%, X.2 6%, 1.2 3%, 1.3 3%, 2.3 3%, 4.4 3%, 5.3 3%, X.3 3% |
| 51 | exercise_n | 347 | 5.1 70%, 5.3 10%, 1.3 6%, X.3 6%, 4.3 3%, X.1 3% |
| 52 | enjoy | 345 | 2.3 16%, 4.7 16%, X.1 16%, 4.3 13%, 1.2 6%, 5.1 6%, 6.3 6%, 1.1 3%, 3.2 3%, 4.2 3%, X.2 3%, X.3 3% |
| 53 | step_n* | 341 | 5.1 30%, 1.3 20%, 1.2 10%, 1.1 6%, 3.2 6%, 4.4 6%, X.1 6%, 3.3 3%, 5.2 3%, 5.3 3%, X.2 3% |
| 54 | skill | 335 | 5.1 53%, 4.7 13%, 1.2 6%, 1.3 6%, 4.3 6%, 1.1 3%, 4.6 3%, 6.3 3%, X.3 3% |
| 55 | struggle_n* | 334 | X.1 20%, 1.2 13%, 4.5 13%, 4.7 13%, 1.3 6%, 4.1 6%, 5.1 6%, 6.2 6%, 1.1 3%, 2.1 3%, 3.3 3%, 6.1 3% |
| 56 | open_adj | 332 | 6.2 30%, 1.2 23%, 3.3 6%, 4.5 6%, X.2 6%, 1.3 3%, 2.3 3%, 2.4 3%, 4.1 3%, 4.7 3%, 5.1 3%, 5.3 3%, X.1 3% |
| 57 | choose* | 330 | 5.1 23%, 1.2 16%, 2.3 10%, 4.5 10%, 3.3 6%, 4.1 6%, 6.2 6%, X.1 6%, 1.3 3%, 4.7 3%, 5.2 3%, 5.3 3% |
| 58 | community | 329 | 1.1 26%, 4.7 16%, 1.3 10%, 1.4 6%, 3.3 6%, 4.4 6%, 1.2 3%, 2.1 3%, 2.3 3%, 4.1 3%, 4.3 3%, 5.2 3%, 5.3 3%, X.2 3% |
| 59 | risk_n | 321 | X.1 30%, 5.3 23%, 4.5 20%, 2.1 6%, 4.7 6%, 1.2 3%, 3.3 3%, 5.2 3%, X.2 3% |
| 60 | diet_n | 320 | 5.1 83%, X.1 6%, 5.3 3%, X.2 3%, 3.3 3% |
| 61 | improve | 318 | 4.3 40%, 5.1 20%, 1.2 13%, 4.7 6%, 5.3 6%, X.1 6%, 3.2 3%, 5.2 3% |
| 62 | member | 307 | 1.3 26%, 1.1 23%, 1.2 16%, X.1 10%, 6.2 6%, X.2 6%, 1.4 3%, 3.3 3%, 4.7 3% |
| 63 | daughter | 297 | 4.5 53%, 1.2 13%, 1.3 10%, 3.3 6%, X.1 6%, X.2 6%, 2.2 3% |
| 64 | perspective | 295 | 1.1 20%, 1.2 13%, 1.3 13%, 4.5 13%, 5.1 6%, X.1 6%, 2.2 3%, 2.3 3%, 4.1 3%, 5.2 3%, 6.3 3%, 3.2 3%, X.4 3%, X.3 3% |
| 65 | stigma | 293 | 3.3 80%, 6.2 16%, X.2 3% |
| 66 | future* | 289 | 2.4 23%, X.1 20%, 2.3 10%, 4.5 10%, 3.2 6%, 2.1 3%, 3.1 3%, 3.3 3%, 4.2 3%, 4.7 3%, 6.2 3%, X.2 3%, X.3 3%, X.4 3% |
| 67 | responsibility | 284 | 5.1 53%, 1.2 13%, 4.7 13%, 3.2 6%, 3.3 3%, 5.3 3%, 6.1 3%, X.1 3% |
| 68 | resource | 283 | 5.2 33%, 1.3 30%, 1.1 13%, 4.3 6%, 5.1 6%, 2.2 3%, 4.5 3%, 6.1 3% |
| 69 | career* | 279 | 4.7 60%, X.1 10%, 2.1 6%, X.2 6%, 3.3 3%, 4.3 3%, 4.4 3%, 5.1 3%, 6.2 3% |
| 70 | lifestyle | 265 | 5.1 80%, 5.3 6%, 1.3 3%, 3.2 3%, 4.3 3%, 4.4 3% |
| 71 | forward_adv* | 262 | 2.3 23%, X.1 20%, 5.1 10%, 1.2 6%, 2.1 6%, 2.4 6%, 3.2 6%, 3.3 3%, 4.3 3%, 4.7 3%, 5.2 3%, 5.3 3%, X.2 3% |
| 72 | offer_v | 259 | 1.3 30%, 1.1 16%, 4.7 13%, 1.2 10%, X.2 10%, 5.3 6%, 4.3 3%, 4.5 3%, 6.2 3%, X.1 3% |
| 73 | success | 247 | 2.3 16%, 5.1 13%, 2.2 10%, X.1 10%, 1.2 6%, 3.2 6%, 4.3 6%, 4.7 6%, 5.3 6%, 1.3 3%, 2.1 3%, 4.4 3%, 6.1 3%, 6.2 3% |
| 74 | guilt | 241 | 3.2 26%, X.1 20%, 2.3 13%, 1.2 10%, 4.1 10%, 5.1 10%, 4.2 3%, 4.5 3%, X.2 3% |
| 75 | maintain* | 239 | 5.1 46%, 1.2 26%, 4.3 6%, X.3 6%, 1.4 3%, 2.3 3%, 4.7 3%, 5.3 3% |
| 76 | society | 237 | 3.3 23%, 4.5 20%, X.1 20%, 1.4 6%, 4.1 6%, X.4 6%, 2.1 3%, 2.3 3%, 3.1 3%, 3.2 3%, 6.2 3% |
| 77 | routine | 233 | 5.1 80%, 4.3 6%, 5.3 6%, 1.2 3%, 4.7 3% |
| 78 | involve | 232 | X.1 33%, 1.2 16%, 1.3 6%, 4.1 6%, 4.3 6%, 1.1 3%, 1.4 3%, 2.2 3%, 3.3 3%, 4.4 3%, 4.5 3%, 5.2 3%, 5.3 3%, X.2 3% |
| 79 | graduate | 228 | 4.7 76%, X.2 16%, 3.3 3%, 4.4 3% |
| 80 | recovery | 227 | 5.1 16%, 2.1 13%, 5.3 13%, 5.2 10%, X.1 10%, 1.1 6%, 2.2 6%, X.2 6%, X.3 6%, 1.2 3%, 1.3 3%, 4.7 3% |
| 81 | schedule* | 224 | 5.1 73%, 4.7 10%, 6.1 6%, 1.2 3%, 1.3 3%, X.3 3% |
| 82 | girlfriend | 220 | 1.2 36%, 1.3 20%, X.2 20%, 4.3 6%, X.1 6%, 1.1 3%, 3.1 3%, 4.5 3% |
| 83 | activity | 220 | X.1 36%, 5.1 26%, 4.3 10%, 1.2 6%, X.2 6%, 1.4 3%, 4.2 3%, 5.2 3%, X.3 3% |
| 84 | exercise_v | 218 | 5.1 66%, 4.3 10%, X.1 10%, 5.3 6%, 2.1 3%, 3.2 3% |
| 85 | marry | 213 | 1.2 33%, 4.3 16%, 1.3 13%, X.1 13%, 4.5 6%, X.2 6%, 3.2 3%, 4.1 3%, 5.2 3% |
| 86 | provide* | 210 | 1.3 36%, 4.5 13%, 1.1 6%, 4.7 6%, 5.1 6%, 5.2 6%, X.1 6%, 4.1 3%, 4.3 3%, 5.3 3%, 6.2 3%, X.2 3% |
| 87 | raise_v* | 209 | 4.5 36%, 4.1 6%, 4.2 6%, 4.7 6%, 5.3 6%, X.2 6%, X.3 6%, 2.1 3%, 2.2 3%, 2.3 3%, 3.3 3%, 4.3 3%, 6.2 3%, 1.2 3% |
| 88 | opportunity | 207 | 2.3 36%, 4.7 23%, 4.5 6%, 6.1 6%, X.3 6%, 1.1 3%, 1.2 3%, 1.3 3%, 5.1 3%, 6.2 3%, X.1 3% |
| 89 | brother* | 207 | X.1 33%, X.2 20%, 1.2 10%, 1.3 10%, 2.2 6%, 3.3 3%, 4.1 3%, 4.3 3%, 4.7 3%, 5.3 3%, X.3 3% |
| 90 | deserve* | 207 | 1.2 30%, 3.2 23%, 2.1 10%, 4.5 6%, 4.7 6%, 1.3 3%, 3.3 3%, 4.1 3%, 6.2 3%, X.1 3%, X.2 3%, X.3 3% |
| 91 | educate* | 204 | 5.2 46%, 1.3 30%, 3.3 6%, X.1 6%, 2.1 3%, 4.3 3%, 6.2 3% |
| 92 | successful* | 199 | 2.2 26%, 4.7 26%, 3.2 10%, 2.3 6%, 2.4 6%, 4.5 6%, 5.1 6%, X.2 6%, X.1 3% |
| 93 | professional | 191 | 1.3 46%, 4.7 20%, 5.3 13%, X.3 6%, 3.3 3%, 4.3 3%, 5.2 3%, X.1 3% |
| 94 | friendship | 190 | 1.2 53%, X.1 30%, 1.1 6%, 6.2 6%, 2.3 3% |
| 95 | progress | 190 | 2.3 26%, 2.1 16%, 1.2 10%, 4.7 10%, 4.4 6%, 5.1 6%, 6.3 6%, 1.3 3%, 4.3 3%, 5.2 3%, X.1 3%, X.3 3% |
| 96 | trauma | 188 | X.1 30%, 4.1 26%, 1.3 20%, 1.2 6%, 5.1 6%, 1.1 3%, 2.3 3%, 4.2 3% |
| 97 | teach* | 186 | 4.7 26%, 1.3 20%, 5.1 16%, 2.3 10%, 4.2 10%, 5.2 10%, 1.1 3%, 4.3 3% |
| 98 | sexual | 184 | 4.1 36%, 3.1 20%, 1.2 16%, 5.1 6%, 3.2 3%, 4.3 3%, 4.5 3%, 4.7 3%, 6.2 3%, 6.3 3% |
| 99 | study_v | 181 | 4.7 46%, 1.3 13%, 5.1 6%, X.1 6%, 1.2 3%, 3.2 3%, 4.2 3%, 4.5 3%, 5.2 3%, 5.3 3%, 6.1 3%, X.2 3% |
| 100 | recover | 180 | X.1 43%, 2.1 16%, 4.7 10%, X.2 10%, 1.3 6%, 4.3 6%, 4.5 3%, 4.6 3% |
| 101 | motivation | 178 | X.1 50%, 1.3 6%, 4.7 6%, 6.3 6%, 1.1 3%, 1.2 3%, 3.2 3%, 4.1 3%, 4.5 3%, 5.1 3%, 5.3 3%, 6.2 3%, X.2 3% |
| 102 | effort* | 178 | 5.1 30%, 1.2 10%, 1.3 10%, 2.3 10%, 4.7 10%, 5.2 10%, 3.2 6%, 1.1 3%, 1.4 3%, 4.5 3%, X.2 3% |
| 103 | belief | 177 | 4.2 36%, 1.3 13%, X.1 13%, 3.1 10%, 5.1 10%, 3.3 6%, 6.2 6%, 4.1 3% |
| 104 | hobby | 177 | 4.3 70%, 5.1 16%, X.1 10%, 4.4 3% |
| 105 | baby* | 174 | 4.5 53%, 5.3 13%, 1.2 3%, 3.2 3%, 3.3 3%, 4.1 3%, 4.2 3%, 4.7 3%, 6.2 3%, X.1 3%, X.2 3%, X.3 3% |
| 106 | strength | 170 | 2.3 26%, 2.2 13%, 3.2 13%, X.1 10%, 3.1 6%, X.3 6%, 1.1 3%, 1.2 3%, 1.3 3%, 2.1 3%, 5.1 3%, 5.2 3%, 6.1 3% |
| 107 | challenge | 168 | 4.5 16%, 5.1 13%, X.1 13%, 1.2 10%, 2.3 10%, 4.1 6%, 1.3 3%, 2.1 3%, 2.2 3%, 4.7 3%, 5.2 3%, 5.3 3%, 6.1 3%, 6.2 3%, X.3 3% |
| 108 | environment* | 168 | 4.5 30%, 5.1 16%, 4.1 13%, 1.3 6%, 3.3 6%, 4.7 6%, X.3 6%, 4.2 3%, 4.3 3%, X.1 3%, X.4 3% |
| 109 | possibility | 166 | 5.3 20%, X.1 16%, 4.5 13%, 1.3 10%, 4.7 10%, X.2 6%, 1.2 3%, 2.2 3%, 2.4 3%, 4.2 3%, 5.1 3%, 6.2 3%, 6.3 3% |
| 110 | adult* | 165 | 6.2 16%, X.2 13%, 1.3 10%, 4.3 10%, 5.1 10%, 4.5 6%, 4.7 6%, 1.2 3%, 2.1 3%, 3.1 3%, 3.2 3%, 3.3 3%, 5.2 3%, X.1 3%, X.4 3% |
| 111 | marriage* | 161 | 1.2 40%, X.1 30%, 1.3 10%, X.3 10%, 4.3 3%, 4.5 3%, X.2 3% |
| 112 | shame_n | 159 | 3.3 23%, 3.2 20%, X.1 20%, 1.3 16%, 4.1 6%, 2.2 3%, 2.3 3%, 5.1 3%, X.3 3% |
| 113 | capable | 158 | 4.7 26%, 4.5 13%, 1.2 6%, 1.3 6%, 3.2 6%, 5.1 6%, 2.3 3%, 3.3 3%, 4.2 3%, 4.3 3%, 4.4 3%, 4.6 3%, 5.2 3%, 5.3 3%, X.1 3%, X.3 3% |
| 114 | education | 157 | 4.7 46%, 5.2 20%, 3.3 6%, 4.3 6%, 1.1 3%, 1.2 3%, 1.3 3%, 4.4 3%, 5.1 3%, X.1 3% |
| 115 | responsible | 156 | 1.3 16%, 4.5 13%, 1.2 10%, 3.3 10%, 5.1 10%, X.1 10%, 1.1 6%, 4.3 6%, X.2 6%, 3.2 3%, 4.7 3%, 5.3 3% |
| 116 | engage | 155 | 1.2 40%, X.1 26%, 1.3 16%, 4.3 6%, 4.7 6%, 5.1 3% |
| 117 | bear_v* | 155 | 4.5 43%, X.1 30%, 3.3 10%, 5.1 6%, 1.3 3%, 3.2 3%, 4.3 3% |
| 118 | encourage* | 155 | 1.3 23%, 2.2 13%, X.1 13%, X.2 10%, 1.1 6%, 2.3 6%, 6.2 6%, 1.2 3%, 3.2 3%, 5.1 3%, 5.2 3%, 6.3 3%, X.3 3% |
| 119 | confidence | 153 | 3.2 40%, X.1 36%, 6.3 10%, 1.2 3%, 2.1 3%, 5.3 3%, X.2 3% |
| 120 | cure_v | 147 | 2.1 26%, 5.3 16%, 5.1 13%, 1.1 6%, 4.2 6%, X.1 6%, X.2 6%, 2.2 3%, 3.1 3%, 3.2 3%, 4.3 3%, X.3 3% |
| 121 | guilty | 147 | X.1 36%, 1.2 20%, 3.2 10%, 4.3 6%, 4.5 6%, 4.7 6%, 1.3 3%, 2.2 3%, 3.3 3%, 5.1 3% |
| 122 | childhood* | 147 | 4.1 40%, X.2 16%, 4.5 13%, 1.2 6%, 3.2 6%, X.1 6%, 2.2 3%, 4.7 3%, X.3 3% |
| 123 | forgive* | 147 | 1.2 40%, 3.2 36%, 4.5 6%, X.1 6%, 2.3 3%, 5.1 3%, 5.2 3% |
| 124 | active | 141 | 1.3 40%, 5.1 26%, 4.3 6%, 1.2 3%, 1.4 3%, 2.3 3%, 2.4 3%, 3.2 3%, 3.3 3%, 4.7 3%, 5.2 3% |
| 125 | insight | 140 | 1.1 33%, 4.1 26%, 1.3 16%, X.1 16%, 3.2 3%, 4.2 3% |
| 126 | pursue | 140 | 4.7 20%, 1.3 10%, 5.1 10%, X.1 10%, 1.2 6%, 2.4 6%, 4.4 6%, 2.3 3%, 3.2 3%, 4.1 3%, 4.2 3%, 4.3 3%, 5.2 3%, 5.3 3%, 6.1 3%, X.3 3% |
| 127 | grateful | 139 | 2.3 36%, 1.3 20%, 1.1 6%, 1.2 6%, 2.2 3%, 3.3 3%, 4.1 3%, 4.2 3%, 4.5 3%, 4.7 3%, 5.3 3%, 6.2 3%, X.3 3% |
| 128 | connect | 136 | 1.2 23%, 1.1 13%, 1.3 10%, 1.4 6%, 2.3 6%, 4.2 6%, 5.1 6%, X.1 6%, 2.2 3%, 4.1 3%, 4.3 3%, 4.5 3%, 5.2 3%, 5.3 3% |
| 129 | quality* | 136 | 5.3 20%, X.1 20%, 4.3 10%, 4.7 10%, 1.2 6%, 1.3 6%, 4.2 6%, 5.1 6%, 2.1 3%, 2.3 3%, 3.2 3%, 4.5 3% |
| 130 | position* | 134 | 4.7 43%, 1.2 10%, 1.3 10%, 1.1 6%, 4.5 6%, X.2 6%, 5.1 3%, 5.3 3%, 6.2 3%, X.1 3%, X.3 3% |

Table S30 POETIC framework descriptions and quotes

| **POETIC index, domain/ category** | *Description (reproduced from* [31, Appendix B & C]*, in italics)* and paraphrased illustrative quotes (see Document S5, normal font) |
| --- | --- |
| **4 Purpose and meaning** | *Broad subdomain of having the goal of living a meaningful and purposeful life whatever that means to the person, goal of recovery is to find framework that explains experience* |
| 4.1 Meaning of mental illness experiences | *Understanding or finding meaning in one’s mental illness experience, can range from spiritual or religious meanings through to adoption of a medical view of mental illness. Giving meaning normalises the experience and can have positive implications (e.g., increased acceptance and reduced self-stigma). Includes people trying to make sense of diagnosis/symptoms due to past life experiences (e.g., trauma).*  “Did you also find positive elements in negative symptoms of our illness? Before I received treatment I experienced a very strong critical voice during depression, but it actually drove me to improve myself. It would motivate me to put more effort into everything and make extreme changes. Now that I receive treatment, I can still identify areas in my life where I might improve, but I don't feel the same motivation. I seem to be happy to live a mediocre life. Does anyone else feel like this, too? Do you have any strategies to overcome this?”  “I've developed two explanations as to why I was chosen to have bipolar. 1. There are tons of people that are bipolar/depressed/etc., and it is a godsend to meet someone that not only understands, but has dealt with it and can help. 2. This is sort of a religious thing, but maybe you can get something out of it anyway. I'm a Buddhist practitioner, so karma comes into play here. With all if this terrible crap I've had to deal with, I'm at least thankful that I am expending so much negative karma in a single lifetime. Getting this out of the way now means that I'm getting it over with.”  “As a teacher, I find myself really good at spotting, helping understanding students with anxiety, low self esteem, depression and the ones being bullied. I have a much better feel with them, than colleagues, who haven't experienced mental health problems and often, these children seek me.” |
| 4.7 Paid or voluntary work or formal education | *Importance and impact of paid work or voluntary work. Paid work or voluntary work provides routine, connection to daily life, identity different from illness, but can also pose a risk factor due to causing stress. Symptoms can impair work performance.*  “I want medication to improve my concentration - is that selfish? When I'm normal or before my hypomania gets out of control, I am excellent at my job, but during depressive and mixed episode I only perform at the most basic levels. So I can hold down my job but I would like to excel. I feel guilty asking my doctor for medication to improve my concentration; on the other hand I think these issues are due to my bipolar, so it is alright wanting to treat it like anything else.”  “Bipolar folks at college: how do you cope with settling into college life after living at home?” |
| 4.3 Quality of life | *Broad subdomain, process and outcome, importance to have basic needs met (adequate housing and financial support). Includes generic mentions of “quality of life” as important for people. Also: people saying their general experience of living has improved, might be due to material changes (“getting a job meant I had my first secure housing”) or psychological/behavioural changes (“my life improved as I was able to accept, I need to ask for help when my mood worsens").*  “Besides, it's no measure of health to be well adjusted to a profoundly sick society.”  “Now the sleep thing is happening again. I also have a job. It's a great job in a pharmacy I like, but some days I just cannot get out of fucking bed. And when I say some days, I mean most. About 8 months ago my boss had to pull my health insurance because I wasn't netting 20 hours a week. [...] I have no health insurance so I cannot see a doctor. Well, I could, but it would cost quite a bit of money out-of-pocket, money which I don't have because I work so few hours. A vicious cycle is happening. I haven't been able to afford my meds so I stopped them a few months ago. At that point, I wasn't convinced they were doing anything anyway, but couldn't see a doctor to get adjusted or re-diagnosed.”  “Mental health professionals call me ‘high functioning’ because I’ve got a job and something looking like a social life. Others with chronic (mental) health issues I know would love to be as ‘put together’ as I am, so I agree on that. However, I need a lot of resources and faking to keep this up. On days when getting out of bed feels almost impossible, I do my best to paint on a smile and get through the day. I am grateful for everything I can do given my bipolar disorder, but no one sees how hard it is to keep going as if everything was okay, how ‘high functioning’ feels inside.” |
| 4.5 Meaningful life and social roles | *Activities and occupations that make life meaningful, essentially the same ones as for people without mental health problems. Life making more sense through re-framing of experience as meaningful, social role of mother/father being reengaged with, finding value in supporting others. Work (paid or voluntary formal employment) is coded at 4.3.3 instead.*  “I wouldn't wish bipolar disorder on my worst enemy, let alone could I bear to pass it on to anyone. I've got other reasons, too. I'm terrified of the thought of having to come off my medication for at least nine months, particularly with the hormonal changes and stress of parenting. I see my suicide risk going up and I'm scared I'd be so selfish and end my child's life as well. [...] I've considered it all and having kids just doesn't seem possible for me.”  “My parents had no idea how to handle my teenage depression and bipolar. [...] I feel like I'm better equipped to help my son deal with his emotions and negative behaviors than my parents ever were bc I've been though it and the therapy. [...] It's all a process and every day is a new challenge, bit that doesn't mean that you can't be a good parent.” |
| 4.2 Spirituality | *Connecting with a higher power and developing spirituality, often aids to develop an understanding of their experience and provides a source of support and encouragement. Includes organised religion or a specific faith as well as spirituality in abstract terms.*  “Spiritual or psychosis? During my highs, one thing that always happens to me is that I get highly euphoric and expansiveness. I experience moments of intense connectedness to the world around me, almost as if the surroundings and I merge into one being. [...] Talking to my new therapist, I told her how these moments were intensely spiritual to me. To my surprise, she told me that they were bordering on psychotic. Would you all say the same? I'm not sure when one is high enough to become psychotic. I've also heard occasional voices and see random patterns in my visual field. Should I tell my doctors? I don't know what to do, I don't want to lose this beautiful spiritual connection to nature.”  “I meditate and visualize angel wings enfolding me and being flooded with radiating loving light. I realise this sounds quite bipolar. To talk about this part of my inner world to a psychiatrist would require a lot of trust for me. I have always had this active imagination, as if there was another presence just out of reach. I think of it as something I do privately. As long as it does not negatively affect my everyday life, it should not concern anyone.” |
| 4.4 Meaningful social and life goals | *Goal setting in areas outside of mental health services, predominantly change of residence and getting a job or take up further education. Compared to 4.5 Meaningful life and social roles, which is about more long-term “roles”, contains more focused goals such as getting a promotion, a degree. 2.5 is more of a feeling (life worth living, for first time had dreams of a good future) and 4.4 more articulated goals (less affective, more cognitive).*  “There is no such thing as a life wasted. [...] The value of a life is up to the person who possesses that life and no amount of outside influence changes that. [...] Bipolar isn't going to cause me to waste my life bipolar is just a thing I have. I interpret my life as meaningful because I choose to do things that I value. Bipolar has had a tremendous negative impact on my life but because I spend my time doing what I value it can never be wasted.”  “I'm not a bad guy, but … I did so many horrible things, made bad choices for myself and my family, recklessly spent our money, so we ended up homeless and had to move in with my parents. No medication will ever enable me to forgive myself, I think. I just hope they can make me stable enough, so I can go to school, find a job, and make progress towards my dreams. Maybe one day I can be the man my kids and wife need. I want my family back.” |
| 4.6 Rebuilding of life | *Having a purpose or a reason to get up each day, can include volunteering.*  ”I might be capable of maintaining a kind of normal life, yet, I don't know how to do it. I'm not in crisis anymore, but there is no normal that I can return to. […] TLDR; now stable, but worried I've been unstable too long to change my lifestyle.”  “So the day program meant I went home at night and on the weekends and dealt with those daily living skills. If I was overwhelmed or confused etc. I'd have the day program therapists to help.” |
| **2 Optimism and hope** | *Having hope for recovery, can be in terms of various aspects: less symptoms, better social, occupational functioning, quality of life [own]. Includes experiencing a reawakening of hope after despair. Subdomains emphasise the idea that recovery is an active process, rather than something that is ‘done to’ the person. Illustrates that recovery requires self-determination.* |
| 2.1 Belief in possibility of recovery | *Belief (disbelief) that recovery is possible.*  “There's someone on there saying that even if things are good right now and I'm optimistic currently, one day I will realize that being optimistic just can't happen. That I can't overcome bipolar and can only manage it. Everyone faces set backs and people with bipolar are no different. The difference between me and people who think you'll never win is that I keep trying.”  “You can think you're cured, other people think they're an astronaut, or God or whatever makes them happy in whatever moment. From a medical perspective bipolar is a *chronic* illness, not an *acute* illness. So there's technically no 'cure', only lessening of episodes, or periods of no episodes. Now it is technically feasible that you're experiencing a very long period of remission, or symptoms so slight it actually seems that your bipolar is 'cured' as far as *you 're* concerned, and- that's great actually. Feeling better is feeling better, no matter how it comes about. You could be in the middle of long manic/hypomanic episode where you feel so wonderfully great that you're 100% convinced your cured, when you're just feeling wonderful due to the course of the illness itself.” |
| 2.3 Positive thinking and valuing success | *Having an optimistic view of life (bad things will get better), gratefulness for life, setting (small goals) and value progress, little successes.*  “I think the problem here is how you or I or OP measure success. You have to develop your own baseline and go from there. [...] My best days are when I choose life over death. That's a celebration for me and I grow from it. My bipolar and BPD make my life unbearable at times and hard at most. But it is what it is. [...] I just choose...albeit with the help of consistent effort, consistent support, an immense amount of education, exposure, and resources to view it positively.”  “I hate bipolar, yes I do, but you know what? Bipolar has taught me what real love looks like. It looks like loving the same person when I'm manic, depressed, stable, anything. Bipolar has taught me humility and how to swallow my pride when I fucked up a friendship because of an outburst. Bipolar has taught me to appreciate every single good moment I have. Bipolar taught me that I was a god damned fucking warrior every single day of my life fighting a battle I didn't even know existed.” |
| 2.2 Hope-inspiring relationships | *Presence of hope-inspiring relationships, could be with family, friends and professionals or with a higher power, engender a belief that recovery is possible. Also contains the opposite: evidence how professionals can reduce hope and belief in recovery. Includes role models that provide hope for future: hear about success of others, learn from other people's recovery stories.*  “Now im thinking about vlogging about my journey w/ Bipolar disorder. Getting personal on selfmanagement and not only the depressive stuff. But I hope to encourage others by showing them you can still live life to the fullest potential.”  “My grandmother had pretty bad bipolar disorder, so does my dad and at least two other cousins. My father is extremely successful and in a loving marriage with my mom. [...] my father is an amazing man who understands my illness like no one else. My dad is my inspiration! [...] I know my dad's experience is not universal but it gives me hope.” |
| 2.4 Having dreams and aspirations | *Aiming for more (better job, quality of life) than one has in the present [own]. Affective state (feeling of life worth living, having dreams of good future). Concrete cognitive goals coded in 4.4 "meaningful social and life goals".*  “Never let bipolar disorder determine who you are. It is a challenge to live with sometimes, but I believe you can still achieve your hopes and ambitions even if it takes some more effort. I plan to keep pursuing my dream, I hope you can find and follow your passions. feel free to PM me if you want to chat or have questions. Good luck on the road ahead, stay strong and never give up.”  “For example, this week I have been completely reorganizing and cleaning my house, car, and life, and I find it fun and engaging. I am not avoidant of tasks, phone calls, people. My mind is clear and liberated from the many burdens of depression. Most of my thoughts are richly positively and focus on the future and the present-- none of the wretched ruminating from before. I am able to talk myself down from intense emotions. I'm able to forgive myself.” |
| **5 Empower-ment** | *How individuals achieve empowerment, many subthemes focus on mental health services, but theme also includes becoming an empowered citizen.* |
| 5.1 Self-management & personal responsibility | *Individuals take responsibilities for their own recovery, professionals need to allow this; being able to get on with day-to-day life, even when symptoms are present, includes maintaining good physical and mental health, using self-management strategies, accessing self-help.*  “I can experience regular emotions now and it's incredible.”  “I've always been super interested in science and I'm not happy with the outputs of the pharma industry. They only care about profit and no one ever fully recovering. Without medication, I had to be my own scientist. I started tracking my mood, sleep, period, diet, anything that seemed relevant. I subtracted and added factors that seemed to have an effect on me. I've much improved, but it's still a work in progress.”  “I'm diagnosed with rapid-cycling bipolar and my sexual needs are pretty demanding. Now that I am taking medication and more stable, swinging and BDSM with my boyfriend basically solved my issues. I want to spend the rest of my life with my boyfriend, but we both agree that you can't always expect one other person to complete you in every way.” |
| 5.3 Controversial role of medication | *Role of medication in recovery, discussions of benefits, disadvantages, and experiences with coming off medication.*  “There is, of course, concerns with safety of mood stabilizer medication while pregnant. From what I understand, there is a risk of cleft lip with lamotrigine. I can tell you people with bipolar disorder do end up having babies. It comes down to weighing the risks of medications versus the pros. It can be more damaging to the baby to not take medication and have mood swings.”  “Care for your wellbeing my ass! How are we supposed to exercise, diet, sleep, when the meds fuck everything all up? Ioved keeping fit but ever since I'm on lithium, I feel exhausted when I exercise. It also reduced my sleep quality, I got even skinner because I eat less and I feel dumber. Still, my doc says lithium is the gold standard and advises against exploring other meds.”  “If you stop taking your medication, you will relapse. This isn’t possible, it’s certain. If your psychiatrist says you need the medication, you should take it. I’m unsure about the legal side, but I’m convinced we have an ethical obligation to take responsibility for our mental health. The most basic thing we can do to protect ourselves and our loved ones is to cooperate with the doctors.” |
| 5.2 Control over life | *Being involved in decision-making and having some say in one’s care and treatment, particularly where medication and hospitalisation are concerned; Positive risk taking in context of care planning and goal setting; helpful if supported by professionals. Feeling in control over life or symptoms, including, but not limited to, in relation to services. 5.1.2 is trying new things even when they might go wrong, 5.2 is feeling in control of life, experiencing agency.*  “Again and again, there comes a point in therapy where the therapist suggests incredibly inappropriate things based on their idealised idea of living life. Like getting a roommate when they do not understand how much I value my own space as an introvert. Of course I resist these suggestions, which they then interpret as me sabotaging my recovery. I welcome suggestions, but in the end, I have to make the decisions for myself.”  “Educate yourself, your family and friends on your diagnosis, and then educate yourself even more, because we'll never fully get it. Stay away from people who want to stigmatise you without understanding your condition. Seek a doctor, know your triggers and symptoms, and advocate for yourself.” |
| **6 Tensions** | *Participants need to negotiate tensions between personal recovery processes and in their dealing with (hypo-)mania.* |
| 6.1 Balancing acceptance with ambitions | *Tension between accepting limitations due to mental health issues to reduce stress and live a manageable life on the one hand and having ambitions or goals to work towards to and not giving up the hope of achieving things meaningful for oneself on the other hand. Negotiating the question: what can I expect of myself?*  “I was forced to come to terms with the fact that being an attorney would likely never be a sustainable, healthy career choice for me, and that was such an ego blow; however, being a paralegal sets me up with a more reliable and less intense schedule with far less client contact, and I still get to work in a field that values my skills (albeit with less remuneration than would be provided were I an attorney).”  “Journeys and progress are rarely linear. Expectations do little for us but lead to disappointment. Possibility is a different story. It means we are open to alternatives and are more adaptable in our thinking- which generally leads fewer instances of feeling stuck.” |
| 6.2 Openness enables support and stigmatisation | *Tension between having to disclose mental health issues to professionals or family/friends to get support with the risk of experiencing stigma. Informal level: Openly/naturally talking about ones BD enables more connectedness with others, more support and less need to hide a part of oneself but also makes one vulnerable to negative judgments, lack of comprehension. Formal level: Different experiences or evaluations of receiving the diagnosis. For some it is helpful explanation for experiences and enables access to treatment, for others it is unhelpful and adds stigma.*  “Having bipolar doesn't somehow delegitimize every emotion I now have. I usually don't respond well if someone tells me to "take my meds" in an emotional situation. People in my life know not to use that as a weapon. It's not an open topic for discussion and I've set that boundary with everyone in my life.”  “Nearly a year after my bipolar diagnosis, I'm finally open about it - it was the best decision. It has allowed me to find the people who are truly there for me and it has strengthened many relationships and widened my support network. It's awesome - I've turned something society considers isolating into an opportunity for support and connection. There are always going to be crappy people, but they would have been that way anyways, even if they didn't know about your bipolar.” |
| 6.3 Ambivalence around (hypo-) mania | *Perception and personal meaning of mania/elated mood varies between individuals: Some participants like and miss mania due to improved creativity and energy. Others dislike or fear elated states due to irritability, danger of losing control. Many are ambivalent about elated states due to these opposing experiences.*  “I miss my joyful hypomanias, I must admit, but I realise the positive experiences are rare and mostly prevent me from getting any real work done.”  “Mania is also part of my problem. There's no way back if I let it drag me. I know it doesn't last forever, I'll see the consequences of my delusional actions and then go downhill and get depressed. But I find it so hard to not engage. Mania is this really cool friend, I love hanging out with her, I feel good all the time, everything is beautiful, I love everyone and everyone loves me. This time, I want to stay on earth, I don't want to do it again. Can anyone relate? Has anyone successfully escaped mania before?”  “Some might discourage this, but I used my bipolar to my advantage in my studies. I found a way to focus my hypomania, getting almost obsessed with the subject. I managed to 'control' my hypomania as a study superpower and to shut it off after the test. I could also shut it off at night to sleep.” |
| **3 Identity** | *How individuals see themselves or think they are perceived by others.* |
| 3.2 Rebuilding or redefining positive sense of self | *Recovery involves redefining or regaining a positive sense of self. Includes viewing the process of recovery as involving some transformation of identity (discovering a new ‘me’) as well as the opposite: holding on to the aspiration of reclaiming one’s prior social identity and sense of self.*  “In the past year, I've learned to let go. I've been cleaning out my belongings and trying to get all of my responsibilities handled (I've been procrastinating, of course...). I've let go of friends and family who aren't supportive of me and are unhealthy for me and my illness. I've let go of some old dreams. I've learned that I'm stronger than I think, but that I tell people that I'm fine when I'm not. I've learned that leaning on loved ones for support is okay, and being vulnerable can lead to growth. I've stopped hiding so much, especially from myself. I try to accept the cycles of being bipolar, and I try to enjoy the moments that make everything worthwhile.”  “You feel guilty and you feel you deserve it. You should have acted differently and yet you haven't. That's only part of being human and especially of being bipolar... To think in terms of deserving is pointless - you deserve a healthy brain & fair change in life, but you will never have that. "Deserved" guilt will not prevent future mistakes. Only experience, rationality, reflection and insight will stop them. Practice mindfulness and radical self-acceptance. Be as kind to yourself as you would be to me for making such mistakes. You're handling this deadly disease as best as you can.”  “Personally, I've always encouraged self-care. Being overly critical about our decisions and disability can just make things worse. The fact is, this is a chronic illness. And it's not easy.” |
| 3.3 Over-coming stigma | *Regain a positive sense of self by overcoming stigma, society needs to support notion of recovery, too, and let go of stigma. Relates specifically to marginalised identities, including but not limited to mental health (so also includes, e.g., gender, sexuality, class).*  “I think a non-aggressive approach would be the best way to deal with this. It might be very tempting to argue with the person and say, "You have no idea what I'm going through!" or "You're stupid if you can't understand this!", but when you take a step back, acting like that will only further this person's belief that you are unstable and unable to fit into the normal fabric of society. I'm sorry if this sounds harsh, but although we are afflicted with bipolar disorder, we need to blend into a society that has certain expectations of behavior. Rebelling against those expectations might be satisfying in the short term, but could be devastating in the long term.”  “Being bipolar puts us at high risk of ending up in the meat grinder that is American Justice, where it's OK for the police to execute a homeless man with an M16 for "camping without a permit". With access to adequate treatment and an end to extreme law enforcement abuses, we would be a much smaller share of the homeless population. Our share of the homeless and incarcerated has more to do with the state of medical science, as well as how our society is structured and conducts itself than our abilities.”  “I don't use the label (yet). I use the words depression, anxiety and panic, and 'manic episode'… If the person is not bipolar and has no clue what a manic episode is (I.e. most everyone else) then they don't have the stigma attached and are just a bit more supportive and less scared and freaked out... Kind of like calling yourself a freethinker instead of atheist, or using 'plant based diet' instead of vegan. Just sidesteps stigma and stereotypes so you can have a real discussion.” |
| 3.1 Dimensions of identity | *Individuals see themselves as more than their diagnosis. Identity not unitary, multiple dimensions important (e.g., sexual, ethnic, cultural). Mental health services/professionals need to value diff. identity dimensions and treat person as individual, not label. Difference to 3.3: Maintain or develop positive sense of self/place in world. E.g., person with unlovability schema due to upbringing who identifies this being replaced with more positive sense of self coded here, not 3.3.*  “But really, bipolar is just... you. It's nice to be on drugs and more stable, but I'm not cured. Bipolar is just part of me. Not always a pleasant part, but it's not going to go away. You might as well embrace it.”  “Thanks so much for the tips on the bipolar part and for bringing up the LGBTQ aspect in detail. I'm very lucky that I am applying to a progressive program that is dedicated to educating students from a social justice-forward perspective in order to serve the very diverse schools here in San Francisco. So, I'm definitely looking forward to sharing my perspective as a lesbian (who cannot "pass" as straight), and continuing to advocate for others in my community whose experiences aren't necessarily like my own.” |
| **1 Connectedness** | *The sense of being connected to others, beyond individual's relationship, includes connections with society and feeling connected to others, subsumes "social inclusion".* |
| 1.3 Support from others | *Relationships indexed on the mental health issues, e.g., with workers (1.3.1) or supportive family (1.3.3) or friends / peers (1.3.4) which may be expressed through practical support (1.3.5).*  “Well, the first thing I might say is the best thing you can do when you decide you want to be there to support someone with a mental illness is to step up your self care regime. [...] Because, unfortunately, the world is not set up to support the supporters. And that shit's fucked. We need you guys and everyone is better off when you can be there for us, but if someone is struggling with regular crises you gotta put your own oxygen mask on before you can be there for someone else.”  “My girlfriend has been with me through my bipolar for about five years. She can be very therapeutic to talk to ...” |
| 1.2 Relationships | *Supportive and collaborative personal relationships seen as having a positive impact on recovery. Having a range of personal relationships with others is critical to the recovery of a life worth living. Different from 1.3, these relationships are not tightly indexed on the mental health issue (though they may emerge from it, e.g., friendships with other people using service), and relate to everyday social connectedness, feeling that you have friends and confidantes.*  “It may be difficult to find someone who is willing to cope with bipolar among 'normal' people pursuing 'normal' life goals. I had the best dating experiences with 'alternative' crowds (tabletop gamers, new age, artists, BDSM, the polyamorous community).”  “Will anyone be able to love me? Can I be in a relationship or will I be single forever?” |
| 1.1 Peer support and support groups | *Importance of peer support, received by attending organised groups, from contact with peer support workers, or informally, from friendships that developed among people with lived experience.*  “Psychotherapy taught me how to overcome my own trauma in a lifelong process and others with similar trauma offered so much solidarity to me.”  “Are there any other "high functioning" bipolar people here who feel like they don't belong anywhere? I can't really relate to others in my bipolar support group with similar symptoms, neither to my peers at school, although I perform at a similar level.”  “I didn’t feel supported in this subreddit. The validity of my bipolar diagnosis was questioned, and it was implied that it wasn’t bad, so I didn’t deserve to post here. I am strong enough to write this now because I hope it starts a discussion for a more supportive environment here. Our bipolar experiences are all different and that should be accepted.” |
| 1.4 Being part of the community | *Feeling of being part of the wider community, could include membership in different communities, e.g., religious, local community, service-related etc.*  “Never give up! If you're not able to go to school/study for one day, it's okay, try tomorrow and see if you can do better.  I totally feel you with the friend thing, what helped me here I set my goal lower. I no longer wanted to make a friend but engage in social activities with people and see where it leads.”  “I work as a representative in Social Security disability hearings, helping people with physical and mental disabilities obtain government benefits. Despite or because of that, I firmly believe that it's best for us to try our best to be productive members of society, even if only to give purpose and motivation for recovery and coping. This group has helped me stay stable.” |

Table S31 Content that did not fit into POETIC: new inductive category descriptions and quotes

| Category | *Description* *(in italics)* and illustrative quotes (normal font) |
| --- | --- |
| **X Not POETIC** | *Content not related to PR and/or the user’s own lived experience (talking about someone else’s experiences or in general terms)* |
| X.1 Other MH issues | *General discussions of MH issues (mostly but not always BD-specific, symptoms, genetics and heredity, treatment, diagnosis, societal issues, scientific research) that are not directly related to PR or users' own experiences*  “My father has bipolar and 2/3 of his offspring, too. His father, growing up at the beginning of the 20th century, was considered “crazy”. My father’s sister also had mental health issues, and I know of at least two cousins on his side that have bipolar.”  “The first step is a proper diagnosis. Based on the symptoms you describe and your family history, you might very well have bipolar, but only a mental health professional can confirm this.”  “Update and burnt bridges Yesterday I posted here about the realization that I've entered a manic episode. I drove 600 miles to see an online friend after breaking up with my boyfriend. I told no one where I was going. Aside from my family and this guy, I've destroyed all my relationship in the past days. I hate it - when I get this way, I become so impulsive and insensitive and I end up feeling super guilty afterwards. I would love to feel normal one day! Positive note: I am safe.” |
| X.2 Storytelling | *Storytelling of users’ own or others' past or present situation without PR relevance*  “This is a very long post, I have the need to tell everything here because I think you will understand me better than other people. I (F/29) had, like many of you I guess, a horrible childhood and teenage years. My family couldn't understand me at all. First time I went to therapy I was 5. I continued with therapy from 5-10, 13-20, 23 and still going.”  “Frankl This book really spoke to me as it was the first book I read after being diagnosed with Bipolar II. Frankl was a holocaust survivor who had studied neurology and psychiatry before being taken away from his family.”  “Within the past WEEK, my gf broke up with me, a cousin of mine passed away to cancer after some partial neglect of my unsupportive family, my grandmother is affected by her death because she feels responsible, I dislocated my shoulder after having a terrible fight on the phone with my gf, my close friend relapsed and overdosed on benzos (she's alive but I went to her house to help her out and she was a mess), and those who I've asked to be close questioned my intentions, and my job has reached an all time level of stress. I don't know what to do.” |
| X.3 Forum interaction | *Direct interactions between forum users (giving/asking for advice, providing example from own life without PR relevance, commenting on another user’s situation, congratulating, wishing well, criticising another user)*  “Your post itself seems more like a troll post rather than an actual post from a parent of a Bipolar child. If I had problems with the law, I sincerely doubt that my parents would seek help from random people on the internet.”  “Misdiagnosed with bipolar, but wanted to thank you guys. I've seen a different psychiatrist, and it's become clear to both him and me that my previous bipolar 2 diagnosis was incorrect, and what I actually have is cyclical depression. However, I wanted to thank you guys for being such a supportive community. I really felt like I had a place I belonged for a while, and I felt like there were people going through what I was at the time, making me feel less alone.”  “There's a lot to unpack here. First of all, she ain't your girlfriend. She's your ex-girlfriend. I ain't trying to rub it in, but her decision must be respected. It sounds like y'all's relationship was rough from the start. It would've been better if it had ended much sooner.” |
| X.4 General discussion | *General discussions of not directly MH-related issues (human behaviour, media, society)*  “Everyone hurts people... normal people hurt people. We subscribe to social conventions as women because we're told to.”  “The majority of the world rules, and runs all important aspects of survival in society. The minority rich, politicians and government are just that small and figureheads.” |

Table S32 Key lemmas that are not PR terms (*) contributing existing and new aspects of the PR experience

| Key lemma | Existing aspect(s) (similar PR terms) | New aspect(s) |
| --- | --- | --- |
| childhood* |  | coming to terms with (traumatic) childhood experiences, collocates: traumatic, trauma, abuse, ptsd - making sense of MH experiences |
| encourage* |  | encouraging others to do something (e.g., seek help) - giving advice / interaction |
| environment* |  | focus on structural/societal circumstances rather than individual (work environment, (un-)supportive environment, family environment) + reproductive decision making (environment for child to grow up) |
| **adult*** |  | life stage: people who are young adults talk about transition/differences between life as child vs. adult; being/behaving like an adult seen as providing for oneself, mature dealing with emotions, taking responsibility for own mental health |
| baby* |  | reproductive decision making |
| raise_v* |  | reproductive decision making |
| bear_v* |  | reproductive decision making, genetics |
| educate* | 112/204 instances are reflexive (educate yourself) | non-reflexive instances: educating others (family, friends, society) - relationships/stigma |
| grow* | personal growth (growth, growth process) - rebuilding/redefining positive sense of self | 221/358 instances are "grow up" - childhood experiences - meaning of MH issues + reproductive decision making |
| teach* | empowerment (learn), job/work (teaching as job/career) | educating others |
| **our*** | relationships (collocations: relationship, son, marriage, family, daughter, child) | group identity: people with BD (collocations: brain, life, ability, illness, emotion, mind, experience, disorder, gene, problem, condition, mood), society in general (collocations: society, community) |
| choose* | self-management & personal responsibility/Empowerment (choice, collocations: people, life, own, tell, behaviour, medication) | negotiating individual responsibility for BD (collocations: illness, bipolar, disorder) + reproductive decision making (collocations: kid, parent, child) |
| forgive* | 72/147 instances reflexive (forgive yourself) | non-reflexive instances: others forgive the user, user forgives others - relationships |
| provide* | others provide support/care/love | reproductive decision making (provide for a child) |
| struggle_n* | recovery as dynamic/active process (2/334 instances through struggle, make it through, get/work through) | symptoms |
| quality* | quality of life (65/136 instances), relationships (quality time), Identity (personal qualities), controversial role of medication (impact on quality of life) | symptoms: sleep quality |
| forward_adv* | active recovery process (move forward, push forward, step forward, active process) + relationships / meaningful life and social goals (look forward) |  |
| brother* | family relationship (mother, father, mom, dad, daughter, son, aunt, uncle, niece, nephew, grandchild, relative) |  |
| career* | meaningful life & social roles (work, job, occupation, promotion, degree) |  |
| position* | meaningful life & social roles (work, job, occupation, promotion, degree) |  |
| future* | optimism & hope (good future - this PR term does not appear, but 36% of future instances are coded in Optimism & Hope domain) |  |
| successful* | Optimism & hope (success) |  |
| group* | peer support (335/717 instance are 'support group') |  |
| deserve* | relationship (problems) (collocations: relationship, support), rebuilding/redefining positive sense of self ("deserving better") (feel good about yourself, collocations: happiness, good) |  |
| marriage* | relationship (relationship, spouse, partner, boyfriend, girlfriend, husband, wife, hubby, bf, gf) |  |
| step_n* | self-management (comfort zone, work through, change process, seek help, active process) |  |
| effort* | self-management (make it through, through struggles), work |  |
| schedule* | self-management (routine) |  |
| maintain* | self-management (routine, maintain health, maintain healthy) |  |
| seek* | support from others (178/389 instances are 'seek help'), remainder about seeking treatment, medication, second opinion |  |
| yourself* | self-management (take care of yourself - self-care) + rebuilding/redefining positive sense of self (forgive yourself) |  |

Table S33 PR terms that were underused in the PR-BD corpus compared to the reference corpus (freq. = frequency)

| Term | Freq. PR-BD | Freq. Reference | Disper-sion | LL | *P* | LR | Comment |
| --- | --- | --- | --- | --- | --- | --- | --- |
| doctor | 1246 | 6659 | 3.1 | 202.1 | <.001 | -0.6 | medical MH professional |
| high mood | 1 | 5 | 13.3 | 0.1 | 1 | -0.5 | symptom |
| mania | 948 | 4395 | 21.6 | 63.3 | <.001 | -0.4 | symptom |
| manic | 1512 | 6267 | 20.9 | 34.1 | <.001 | -0.2 | symptom |
| pdoc | 297 | 1737 | 2.1 | 73.9 | <.001 | -0.7 | medical MH professional |
| psychiatrist | 974 | 4548 | 0.1 | 68.6 | <.001 | -0.4 | medical MH professional |
| race | 93 | 344 | 0.4 | 0.2 | 1 | -0.1 | race_n (identity-aspect) overused, race_v (not PR-related) underused |
| sleep | 1279 | 4620 | 9.1 | 0.7 | 1 | 0.0 | symptom |

Table S34 PR terms that did not appear in the PR-BD corpus

| Group: Frequency | PR terms (lemmatised) |
| --- | --- |
| Content covered by other key lemmas: 30 | brush yourself off, come off antipsychotic, find your own recovery, find your own route, find something to hang on to, find the proper balance, find way of care, find way of live, make it up to yourself, stand on your own foot, access to service, crisis planning, service user, share decision-make, handle ambivalence, handle distress, hope-inspire, rebuild life, self-belief, self-educate, social role, social success, meaningful activity, meaningful for you, meaningful role, meaningful task, personally meaningful, pivotal moment, pivotal step, pivotal thing |
| Recovery journey characteristics: 12 | active process, change process, dynamic process, growth process, idiosyncratic process, individualized process, process of acceptance, process of recovery, subjective process, unique process, journey of recovery, recovery journey |
| Spelling mistake: 2 | progess, pyhsical activity |
| Islamic faith: 1 | Islamic  Other belief-oriented PR terms are all overused: Christian, Jewish, Buddhist, Catholic, Protestant, atheist, however Muslim and Hindu (not in the PR terms list but related to Islamic faith) are overused in the PR-BD corpus, so discussions of Islamic faith are not entirely absent |
| Collectivist identity: 1 | collectivist  It is unclear whether the absence of *collectivist* indicates the absence of collectivist notions/discussions about different societal norms from the PR-BD corpus or whether the term is just too formal for Reddit. The PR term *individual* is about equally frequent in the PR-BD and reference corpus. |

References

1. Lui M, Baldwin T. langid.py: An Off-the-shelf Language Identification Tool. AclwebOrg [Internet] 2012;(July):25–30. Available from: http://www.aclweb.org/anthology-new/P/P12/P12-3005.pdf

2. Bird S, Loper E, Klein E. Natural Language Processing with Python [Internet]. O’Reilly Media Inc; 2009. Available from: https://www.nltk.org/

3. Cohan A, Desmet B, Macavaney S, Yates A, Soldaini L, Macavaney S, Goharian N. SMHD: A Large-Scale Resource for Exploring Online Language Usage for Multiple Mental Health Conditions. Proc 27th Int Conf Comput Linguist [Internet] 2018. p. 1485–1497. Available from: https://www.aclweb.org/anthology/C18-1126

4. Rayson P, Archer D, Piao S, McEnery T. The UCREL semantic analysis system. Proc beyond named entity Recognit Semant Label NLP tasks Work [Internet] 2004. p. 7–12. Available from: http://eprints.lancs.ac.uk/1783/

5. Piao S, Archer D, Mudraya O, Rayson P, Garside R, McEnery T, Wilson A. A large semantic lexicon for corpus annotation. Proc from Corpus Linguist Conf Ser 2005.

6. Garside R, Smith N. A Hybrid Grammatical Tagger: CLAWS4. In: Garside R, Leech G, McEnery A, editors. Corpus Annot Linguist Inf from Comput Text Corpora London: Longman; 1997. p. 102–121.

7. Hunt D, Brookes G. Corpus, discourse and mental Health. Research i. Teubert W, Mahlberg M, editors. Discourse Ment Heal. Bloomsbury Academic; 2020. [doi: 10.4324/9780203701928]ISBN:9781350059177

8. Baker P. Sociolinguistics and corpus linguistics. Kerswill P, Swann J, editors. Edinburgh University Press; 2010.

9. Hsieh HF, Shannon SE. Three approaches to qualitative content analysis. Qual Health Res 2005;15(9):1277–1288. PMID:16204405

10. Van Mierlo T. The 1% rule in four digital health social networks: An observational study. J Med Internet Res 2014;16(2):1–9. PMID:24496109

11. Carron-Arthur B, Cunningham JA, Griffiths KM. Describing the distribution of engagement in an Internet support group by post frequency: A comparison of the 90-9-1 Principle and Zipf’s Law. Internet Interv 2014;1(4):165–168. [doi: 10.1016/j.invent.2014.09.003]

12. World Health Organization Regional Office for Europe. Mental health action plan for Europe: facing the challenges, building solutions. First WHO Eur Minist Conf Ment Heal [Internet] Helsinki: Copenhagen : WHO Regional Office for Europe; 2005. [doi: EUR/04/5047810/7]

13. Department of Health (UK). No health without A Call to Action [Internet]. 2011. Available from: www.dh.gov.uk/mentalhealthstrategy

14. Amering M, Schmolke M. Recovery: Das Ende der Unheilbarkeit. Psychiatrie Verlag, Imprint BALANCE buch+ medien verlag; 2011. ISBN:3884147838

15. Michalak EE, Hole R, Holmes C, Velyvis V, Austin J, Pesut B, Hou S. Implications for psychiatric care of the word “recovery” in people with bipolar disorder. Psychiatr Ann [Internet] Michalak, Erin E., Department of Psychiatry, University of British Columbia, 2255 Wesbrook Mall, Vancouver, BC, Canada, V6T2A1: SLACK; 2012 May;42(5):173–178. [doi: 10.3928/00485713-20120507-04]

16. McCabe R, Whittington R, Cramond L, Perkins E. Contested understandings of recovery in mental health. J Ment Heal [Internet] Taylor & Francis; 2018;27(5):475–481. [doi: 10.1080/09638237.2018.1466037]

17. Neil ST, Kilbride M, Pitt L, Nothard S, Welford M, Sellwood W, Morrison AP. The questionnaire about the process of recovery (QPR): A measurement tool developed in collaboration with service users. Psychosis 2009;1(2):145–155. [doi: 10.1080/17522430902913450]

18. Cohen J. A Coefficient of Agreement for Nominal Scales. Educ Psychol Meas 1960;20(1):37–46. [doi: 10.1177/001316446002000104]

19. Dettori JR, Norvell DC. Kappa and Beyond: Is There Agreement? Glob Spine J 2020;10(4):499–501. [doi: 10.1177/2192568220911648]

20. Gwet KL. Handbook of Inter-Rater Reliability: The Definitive Guide to Measuring the Extent of Agreement among Raters. Advanced Analytics, LLC; 2014.

21. Brezina V. Statistical choices in corpus-based discourse analysis. Corpus Approaches to Discourse A Crit Rev 2018;259–280. [doi: 10.4324/9781315179346]

22. Gwet KL. Constructing Agreement Coefficients: AC1 and Aickin’s alpha. Handb Inter-Rater Reliab Defin Guid to Meas Extent Agreem among Raters Advanced Analytics, LLC; 2014. p. 101–128.

23. Torgalsbøen AK. Full recovery from schizophrenia: The prognostic role of premorbid adjustment, symptoms at first admission, precipitating events and gender. Psychiatry Res 1999;88(2):143–152. [doi: 10.1016/S0165-1781(99)00077-3]

24. Liberman RP, Kopelowicz A. Recovery from schizophrenia: A challenge for the 21st century. Int Rev Psychiatry Taylor & Francis; 2002;14(4):245–255. PMID:19708804

25. Anthony WA. Recovery from mental illness: the guiding vision of the mental health system in the 1990s. Psychosoc Rehabil J 1993;16(4):11–23.

26. Goldman H, Skodol E, Lave R. Revising axis V for DSM-IV: A review of measures of social functioning. (diagnostic and statistical manual of mental disorders) (special article). Am J Psychiatry 1992;149(9). [doi: https://doi. org/10.1176/ajp.149.9.1148.]

27. Rayson P. From key words to key semantic domains. Int J Corpus Linguist 2008;13(4):519–549. [doi: 10.1075/ijcl.13.4.06ray]

28. Anthony L. AntConc: Design and development of a freeware corpus analysis toolkit for the technical writing classroom. IEEE Int Prof Commun Conf 2005;729–737. [doi: 10.1109/IPCC.2005.1494244]

29. Jurafsky D, Martin JH. Speech and Language Processing 3rd edition draft. 2018. PMID:19878769ISBN:0130950696

30. Honnibal M, Montani I, van Landeghem S, Boyd A. spaCy: Industrial-strength Natural Language Processing in Python. 2020; [doi: 10.5281/zenodo.1212303]

31. Jagfeld G, Lobban F, Marshall P, Jones SH. Personal recovery in bipolar disorder: Systematic review and “best fit” framework synthesis of qualitative evidence – a POETIC adaptation of CHIME. J Affect Disord [Internet] Elsevier B.V.; 2021;292:375–385. [doi: 10.1016/j.jad.2021.05.051]

32. Leamy M, Bird V, Le Boutillier C, Williams J, Slade M. Conceptual framework for personal recovery in mental health: Systematic review and narrative synthesis. Br J Psychiatry 2011;199(6):445–452. PMID:22130746

33. Berry N, Lobban F, Belousov M, Emsley R, Nenadic G, Bucci S. #WhyWeTweetMH: Understanding why people use Twitter to discuss mental health problems. J Med Internet Res 2017;19(4). [doi: 10.2196/jmir.6173]

34. Baker P. Querying Keywords: Questions of Difference, Frequency, and Sense in Keywords Analysis. J English Linguist 2004;32(4):346–359. [doi: 10.1177/0075424204269894]

35. Schmid H. Probabilistic Part-of-Speech Tagging Using Decision Trees. Int Conf New Methods Lang Process [Internet] Manchester; 1994. Available from: https://www.cis.lmu.de/~schmid/tools/TreeTagger/data/tree-tagger1.pdf

36. Horsmann T, Erbs N, Zesch T. Fast or Accurate? – A Comparative Evaluation of PoS Tagging Models. Proc Int Conf Ger Soc Comput Linguist Lang Technol [Internet] University of Duisburg-Essen; 2015. p. 22–30. Available from: https://www.ltl.uni-due.de/wp-content/uploads/posTaggerEvaluation.pdf

37. Brezina V. Statistics in Corpus Linguistics. Stat Corpus Linguist. 2018. [doi: 10.1017/9781316410899]ISBN:9781316410899

38. Rayson P, Berridge D, Francis B. Extending the Cochran rule for the comparison of word frequencies between corpora. JADT 2004 7es Journées Int d’Analyse Stat des Données Textuelles [Internet] 2004. p. 1--12. Available from: http://eprints.lancs.ac.uk/12424/

39. Rayson P, Garside R. Comparing corpora using frequency profiling. Proc Work Comp Corpora, held conjunction with 38th Annu Meet Assoc Comput Linguist 2000. p. 1–6. [doi: 10.3329/akmmcj.v8i1.31665]

40. Hardie A. Statistical identification of keywords, lockwords and collocations as a two-step procedure. Proc Annu Conf Int Comput Arch Mod Mediev English 2014. p. 49. PMID:25246403

41. Jagfeld G. Subreddit topics [Internet]. 2022 [cited 2023 Feb 23]. Available from: https://github.com/glorisonne/reddit_bd_mood_posting_mh/blob/main/data/subreddit_topics.csv

42. Jagfeld G, Lobban F, Davies R, Boyd RL, Rayson P, Jones SH. Posting patterns in peer online support forums and their associations with emotions and mood in bipolar disorder: exploratory analysis.

43. Troiano E, Padó S, Klinger R. Emotion Ratings: How Intensity, Annotation Confidence and Agreements are Entangled. Proc 11th Work Comput Approaches to Subj Sentim Soc Media Anal [Internet] 2021. p. 40–49. Available from: http://arxiv.org/abs/2103.01667

1. r/Bipolar, r/BipolarReddit, r/bipolar2, r/bipolarSOs, r/bipolarart, r/cyclothymia, r/manicdepression [↑](#footnote-ref-1)
2. The BD Subreddit Corpus includes 34,995 comments made in April and May 2019. The S-BiDD dataset contains only posts up until March 2019, to be consistent with the submissions (thread starts), which were only available up until this month when the dataset was created. [↑](#footnote-ref-2)
3. Log likelihood statistic [39], *P*<.0001 with Bonferroni correction for n=490,364 comparisons (number of combined unique terms, part of speech tags, and USAS domains in the BD Subreddit and SMHD Reference Corpus) [↑](#footnote-ref-3)
4. at least 1K occurrences and overused at least twice (log ratio [40] ≥ 1.0) in the BD Subreddit Corpus compared to the SMHD Reference Corpus [↑](#footnote-ref-4)
5. https://www.sketchengine.eu/ [↑](#footnote-ref-5)
6. https://github.com/glorisonne/reddit_bd_recovery/blob/main/results/BD%20Subreddit%20Corpus_key%20terms.csv [↑](#footnote-ref-6)
7. https://github.com/glorisonne/reddit_bd_recovery/blob/main/results/BD%20Subreddit%20Corpus_key%20domains.csv [↑](#footnote-ref-7)
8. https://github.com/glorisonne/reddit_bd_recovery/blob/main/data/BDSubredditCorpus_UntaggedTerms_ProposedTags.csv [↑](#footnote-ref-8)
9. according to automatic language identification via langid.py [1] [↑](#footnote-ref-9)
10. Note that urls in all posts in the S-BiDD dataset were automatically replaced with the placeholder subURLaddress (see https://github.com/glorisonne/reddit_bd_mood_posting_mh/blob/main/replace_urls.py) [↑](#footnote-ref-10)
11. https://github.com/glorisonne/reddit_bd_recovery/blob/main/exploratory_study_2/recover_content_terms.csv [↑](#footnote-ref-11)
12. terms listed in the Oxford dictionary (lexico.com) or their correctly inflected variants, e.g., recoveries [↑](#footnote-ref-12)
13. See also code release https://github.com/glorisonne/reddit_bd_recovery/blob/main/agreement.R [↑](#footnote-ref-13)
14. See Jagfeld [41] and Jagfeld et al. [42] for the lists of BD and MH-specific subreddits. [↑](#footnote-ref-14)
15. <https://github.com/UCREL/science_parse_py_api> [↑](#footnote-ref-15)
16. In this study, the vocabulary consists of the unique lemmas in all posts. The posts were pre-processed such that spaces in all phrases in the PR terms list were replaced by underscores, so they are represented as a single term in the posts (e.g. “believe in myself” 🡪 believe_in_myself). [↑](#footnote-ref-16)
17. https://github.com/glorisonne/reddit_bd_recovery/blob/main/post_ids/posts_PR_relevant_post_ids.csv [↑](#footnote-ref-17)
18. Guidelines for confidence adapted from coding emotionality of sentences [43]. [↑](#footnote-ref-18)
19. Personal e-mail communication with TreeTagger developer Helmut Schmid on 23^rd^ Feb 2022. [↑](#footnote-ref-19)
